# DNA/RNA-binding protein KIN17 supports esophageal cancer progression *via* resolving noncanonical STING activation induced by R-loop

**DOI:** 10.1038/s41392-025-02344-2

**Published:** 2025-08-15

**Authors:** Zichao Wei, Ning Zhao, Lu Kuang, Ji Cong, Sujuan Zheng, Yi Li, Zhihua Liu

**Affiliations:** 1 State Key Laboratory of Molecular Oncology, National Cancer Center/National Clinical Research Center for Cancer/Cancer Hospital, Chinese Academy of Medical Sciences and Peking Union Medical College, Beijing, PR China; 2 Institute of Cancer Research, Henan Academy of Innovations in Medical Sciences, Zhengzhou, Henan China

**Keywords:** Head and neck cancer, Head and neck cancer, Oncogenes, Genomic instability

## Abstract

Targeting the DNA damage response (DDR) exhibits potent efficacy in inducing immune activation and enhancing patient prognosis. However, the benefits of DDR regulation are not universally observed across all patients, owing to the intricate compensatory mechanisms operative in certain cancers. There still exists a gap in the function of activated DDR protein in esophageal squamous cell carcinoma (ESCC). Here, we demonstrate that increased expression of DDR genes contributes to the progression of esophageal squamous cell carcinoma and suppresses the tumor immune microenvironment. Notably, the abundant presence of the DDR protein KIN in ESCC tissues facilitates efficient DNA damage clearance and promotes escape from apoptosis. Depletion of KIN significantly inhibited proliferation and induced DNA damage accumulation in ESCC cells. Mechanistically, KIN functions to support the recruitment of the R-loop regulator DHX9 to R-loop sites, thereby addressing DNA damage associated R-loops. Intriguingly, the depletion of KIN activates the STING pathway *via* NFκB signaling, which is induced by the accumulation of R-loops, ultimately initiating an innate immune response. Depletion of KIN improved the immune microenvironment and the effect of immune therapy in mouse model. Collectively, our findings identify KIN as a novel R-loop binding protein that facilitates the recruitment of the R-loop resolution complex and suppresses tumor-intrinsic innate immunity.

## Introduction

The stability of DNA is crucial for maintaining proper cellular activity. Both endogenous replication errors and exogenous stressors such as radiation pose significant threats to DNA integrity, potentially leading to DNA lesions. To deal with these threats, cells have evolved a highly precise DNA damage response system.^1^ Dysregulation of genes related to DDR is implicated in cancer pathogenesis, as DDR deficiencies may increase the risk of activating oncogenes or losing tumor suppressor genes.^2^

Such existing inherent deficiency also makes tumor cells more reliant on alternative repair pathways, which are not essential under normal physiological conditions, thereby demonstrating the concept of synthetic lethality to induce more DNA damage in cancer cells. Accumulated DNA damage induces neoantigens and the expression of immune checkpoints, consequently activating the immune system to clear abnormal cells. A paradigmatic example is the treatment of BRCA1/2-deficient breast cancer with poly (ADP-ribose) polymerase (PARP) inhibitors, which is deficient in the homologous recombination (HR) DNA repair factor.^3,4^ However, not all patients benefit from these strategies because of the complexity of the DDR compensatory system in certain cancers. Esophageal cancer is of the sixth most common cause that kills cancer patients, and more than half cases of ESCC occur in China. To date, there is no precise targeting strategy for ESCC patients, and their prognosis remains unsatisfactory. Chemoresistance that resulting from innate or acquired DDR activation is a significant obstacle to improving the efficacy of therapy. The function of specific activated DDR genes in supporting tumor progression and their potential as novel therapeutic targets in ESCC warrants further investigation.

DDR deficiency is an important driving force for the R-loop formation, which is an abnormal structure formed by the cross-linking of DNA and RNA.^5^ Many DDR proteins have been found to function in R-loop processing.^6–9^ While some researchers argue that R-loops facilitate DNA repair,^10^ it is crucial for the proper recruitment of downstream factors that R-loops be resolved in time. Therefore, managing R-loops effectively is important for genomic stability.^5,11^ Prolonged accumulation of R-loops will induce the release of nucleic acids, which are subsequently captured by cyclic GMP-AMP synthase (c-GAS).^12–14^ Consequently, c-GAS activated stimulator of interferon genes (STING) triggers cell apoptosis. Excessive accumulation of R-loops has been proved across various syndromes, human neurological conditions, and cancerous cells.^15^ However, these accumulated R-loops have limited effects on improving the tumor microenvironment. The connection between R-loop and tumor microenvironment needs further investigation. KIN has been identified as a homolog of RecA, exhibiting a 47% sequence homology across a 40-residue segment within the C-terminal region of RecA.^16^ The KIN protein can bind to multiple types of nucleotides and is implicated in DNA replication processes.^17,18^ Furthermore, it has been reported that KIN is upregulated and concentrated in the nucleus after DNA damage, where it associates with replication protein A.^19^ KIN has been reported to be upregulated in many tumors and promotes the development of tumors, such as cervical cancer, breast cancer, and liver cancer.^20–22^ However, no research has been conducted in ESCC. Researchers have explored the role of KIN in the survival and metastasis of tumor cells and reported that KIN is closely related to the DDR, but the underlying mechanism has not been studied. Nevertheless, the precise role of KIN in the DNA damage response and ESCC has yet to be elucidated.

In this study, we evaluated the DNA damage response score using transcriptome data from HRA003107 and found a correlation between the DDR score and the immune microenvironment in ESCC tissues. Here, our research revealed that high expression of DNA damage response genes is associated with the tumor immune microenvironment and supports the progression of ESCC. Through analyzing differentially expressed proteins between ESCC tissues and normal tissues, we identified KIN as a pivotal DNA damage response protein that is enriched in ESCC tissues. To investigate the function of KIN, we established KIN knockdown ESCC cell lines and found that depletion of KIN significantly inhibited proliferation and induced apoptosis in ESCC cells. An interactome study of KIN revealed that KIN functions with RNA processing proteins but not classical DNA damage response proteins. Mechanistically, KIN participates in DNA damage response by supporting recruitment of DHX9 to R-loop. Depletion of KIN leads to abnormal accumulation of R-loops in ESCC cells and activates the STING-NFκB pathway. Further, KIN deficiency improved the efficacy of immune therapy in mouse model. Abundant KIN protects ESCC cells from innate immune activation induced by the interferon (IFN) response. In this study, we investigated the mechanism that KIN participates in the DNA damage response. Our findings highlight the importance of the STING pathway, which is activated through the phosphorylation of NFκB during R-loop-induced innate immune activation. Importantly, we identified KIN as a potential target for improving the innate immune response and provided new insight into the role of activated DNA damage response proteins in cancer progression.

## Results

### High expression of KIN in esophageal squamous cell carcinoma

**Fig. 1:**
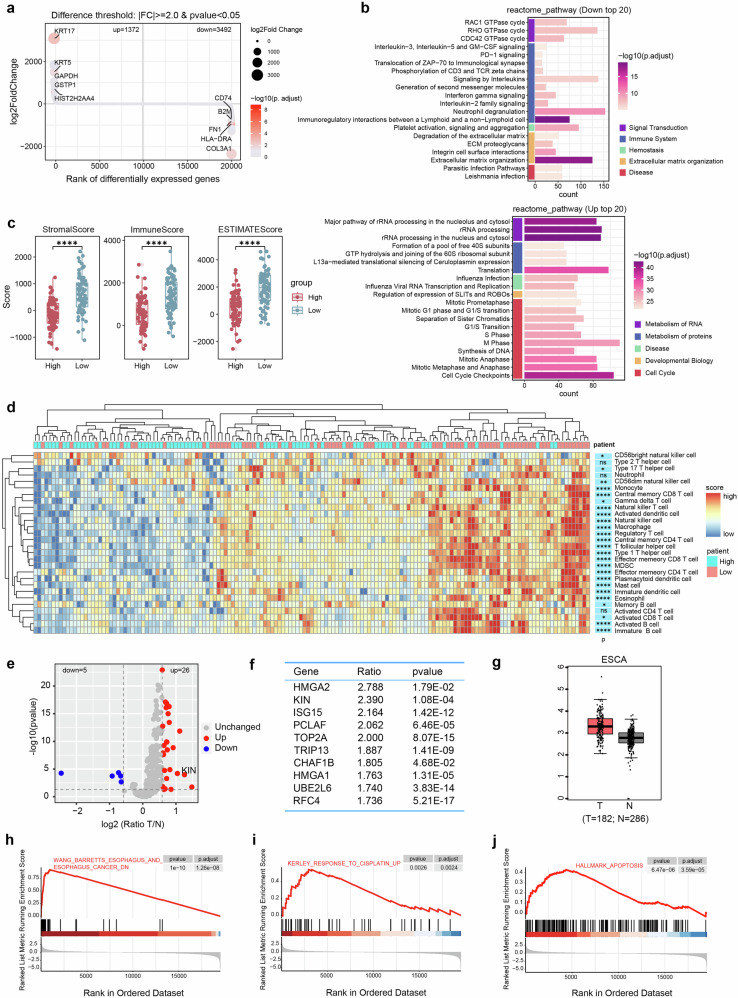
High expression of KIN in esophageal squamous cell carcinoma. **a** Log2FC of differentially expressed genes between DDR-high group and DDR-low group (DDR-high group versus DDR-low group) using data from HRA003107 dataset. **b** Reactome pathway analysis of differentially expressed genes between DDR-high group and DDR-low group using data from HRA003107 dataset. **c** ESTIMATE score in DDR dependent subgroup using data from HRA003107 dataset. Red represents DDR-high group and blue represents DDR-low group. **d** Heatmap shows distribution of subtypes of immune cells using data from HRA003107 dataset. **e**, **f** Differentially expressed DDR proteins between tumor and normal tissues (**e**). Top 10 proteins were showed (**f**). **g** KIN expression in TCGA dataset. **h**–**j** GSEA comparing the gene expression profiles between KIN-high group and KIN-low group using data from HRA003107 dataset. (KIN-low group versus KIN-high group). Statistical significance is indicated as ****P < 0.0001, ***P < 0.001, **P < 0.01, *P < 0.05

To investigate the biological significance of DDR enrichment in ESCC, we utilized the HRA003107 dataset^23^ and leveraged the expression profile of DDR genes to group all 155 patients on the basis of the median of DDR score. Subsequently, we analyzed the differentially expressed genes (DEGs) between DDR-high group and DDR-low group. We observed a set of downregulated immune-related genes (CD74, FN1, HLA-DRA, etc) enriched in the low DDR score group (Fig.1a). REACTOME enrichment analysis of DEGs between the DDR-high and the DDR-low groups revealed that the signaling pathways enriched with downregulated genes were predominantly related to the immune system (Fig. 1b). In addition, the DDR-low group presented increased ImmuneScore, StromalScore, and ESTIMATEScore values (Fig. 1c). We analyzed 28 immune-associated gene sets^24^ within the HRA003107 dataset to describe immune cell subpopulations, and we also noted that the DDR score was negatively correlated with immune infiltration (Fig. 1d). In addition, we observed comparable results in the ESCA dataset from the TCGA dataset (Supplementary Fig. 1). These results suggest that the DDR is involved in the regulation of the immune system and the progression of ESCC.

To identify proteins responsible for maintaining genome stability in ESCC, we incorporated proteomic data^25^ and identified 31 DDR proteins that were differentially expressed between tumor tissues and normal tissues (Fig. 1e, f). We found that the KIN protein was upregulated in ESCC. To further elucidate the role of KIN in ESCC, we analyzed KIN gene expression in ESCC and healthy nontumor tissues from the TCGA database and revealed increased expression of KIN in tumors compared with normal tissues (Fig. 1g). To determine the potential biological functions supported by KIN enrichment, we divided all patients into a KIN-high group and a KIN-low group on the basis of the median expression of KIN and performed gene set enrichment analysis (GSEA) on the DEGs between the KIN-high group and the KIN-low group. We noted that tissues with high levels of KIN presented greater gene enrichment in the malignant phenotypes (Fig. 1h). Additionally, low levels of KIN predicted a greater gene enrichment in response to cisplatin (DDP) treatment (Fig. 1i), suggesting that high levels of KIN confer tolerance to DNA damage. We also observed an enrichment of apoptotic signals in the KIN-low group (Fig. 1j). These results indicate that high levels of KIN facilitate proper processing of DNA damage and contribute to ESCC progression.

### Knockdown of KIN inhibits esophageal squamous cell carcinoma progression

**Fig. 2:**
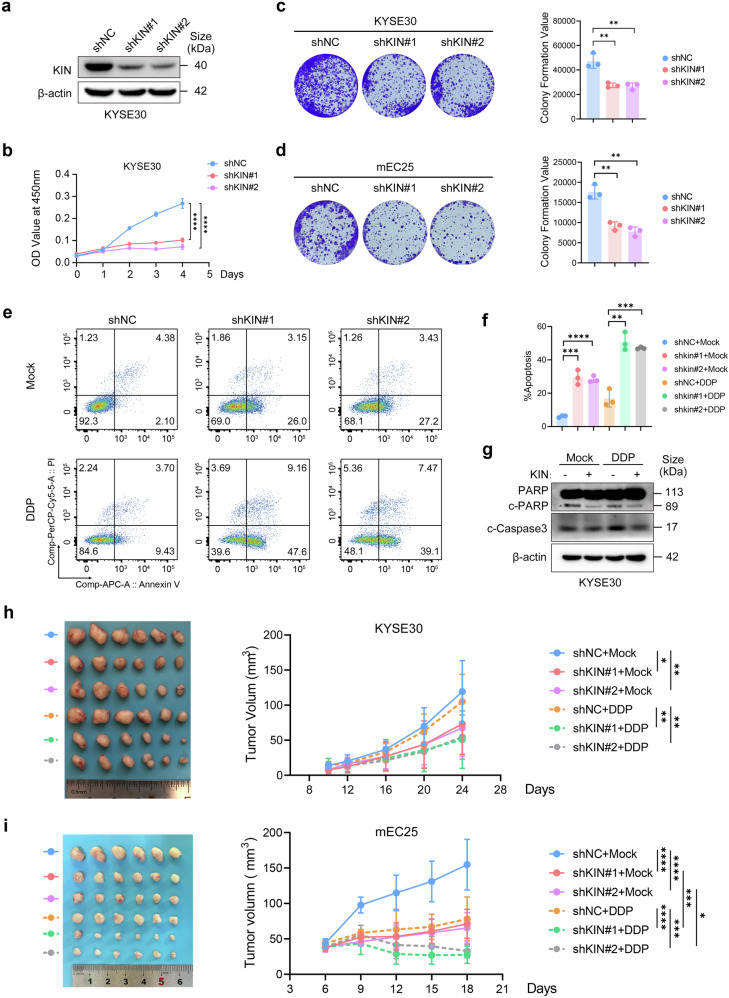
Knockdown of KIN inhibits esophageal squamous cell carcinoma progress. **a** KIN protein expression in KYSE30 cells transfected with shNC or shKIN virus. **b** Growth curves of KYSE30 cell line transfected with shNC or shKIN virus. **c** Representative images (left) and quantification (right) of the colony-forming ability of KYSE30 cell line transfected with shNC or shKIN virus (*n* = 3). **d** Representative images (left) and statistic (right) of the colony-forming ability of mEC25 cell line transfected with shNC or shKIN virus (*n* = 3). **e**, **f** Representative charts (**e**) and quantification (**f**) of flow cytometry results for propidium iodide and annexin V staining in the indicated cell lines after treatment of 5 μg/ml DDP for 12 h. Error bars represent SD obtained from three independent experiments (*n* = 3). **g** Immunoblotting analysis of apoptosis proteins in KIN overexpressed KYSE30 after treatment of 5 μg/ml DDP for 12 h. **h** Tumor growth curves and representative image of shNC and shKIN KYSE30 tumors treated with DDP or PBS (*n* = 6). **i** Tumor growth curves and representative image of shNC and shKIN mEC25 tumors treated with DDP or PBS (*n* = 6). Statistical significance is indicated as ****P < 0.0001, ***P < 0.001, **P < 0.01, *P < 0.05

To gain insight into the impact of KIN enrichment in cancer cells, we established KIN knockdown cell lines through short hairpin RNA (shRNA) (Fig. 2a and Supplementary Fig. 2a, b). Using the CCK8 assay as an indicator of cell viability, we observed significant inhibition of proliferation in ESCC cells following KIN depletion (Fig. 2b and Supplementary Fig. 2c). Similarly, a notable decrease in colony formation ability was observed after KIN knockdown (Fig. 2c, d). Subsequently, we employed flow cytometry with Annexin V and PI as markers for apoptosis detection to characterize the apoptosis profile associated with KIN. KIN-knockdown cells presented robust signals of early apoptosis (Fig. 2e). Following DDP treatment, an increase in both early and late apoptosis was observed in KIN-depleted cells, whereas control cells displayed only a modest increase in early apoptosis (Fig. 2e,f). Additionally, cells overexpressing KIN presented a reduced level of apoptosis-related protein expression (Fig. 2g). To further validate our findings, we injected tumor cells subcutaneously into nude mice after they were transfected with shKIN virus or control virus in nude mice and found that depletion of KIN also inhibited tumor growth in vivo (Fig. 2h and Supplementary Fig. 2d). Then, we established KIN-knockdown cell lines through shRNA in the mEC25 or HNM007 cell lines (Supplementary Fig. 2e, f) and constructed allograft tumors in C57BL/6 J mice. We observed decreased tumor growth in KIN knockdown tumors, and this decrease was enhanced after DDP treatment (Fig. 2i and Supplementary Fig. 2g–i). These results collectively indicate that enriched KIN promotes ESCC progression and facilitates escape from apoptosis.

### KIN is required for genome stability

**Fig. 3:**
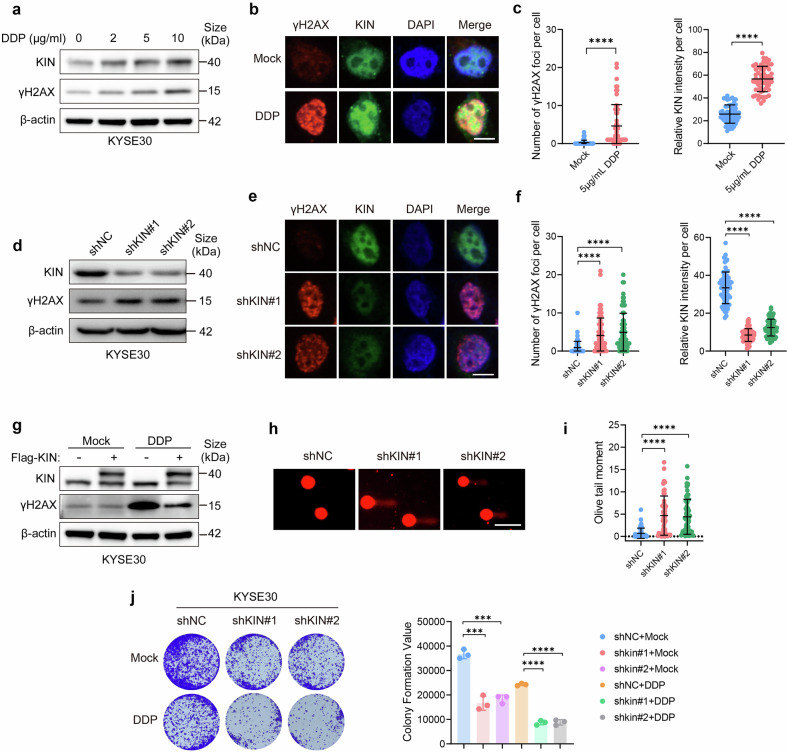
KIN is required for genome stability. **a** Protein expression in KYSE30 treated with different concentration DDP for 2 h. **b**, **c** Fluorescence image (**b**) and quantification (**c**) of γH2AX and KIN in KYSE30 cells treated with 5 μg/ml DDP for 2 h (*n* > 50). Scale bars, 10 μm. **d** Protein level of KIN and γH2AX in shNC and shKIN KYSE30 cells. **e**, **f** Fluorescence image (**e**) and quantification (**f**) of γH2AX and KIN in shNC and shKIN KYSE30 cells (*n* > 50). Scale bars, 10 μm. **g** Protein level of KIN and γH2AX in KYSE30 cells expressed KIN or empty vector treated with 5 μg/ml DDP for 2 h. **h**, **i** Analysis of damaged DNA accumulation by alkaline comet assay in shNC and shKIN KYSE30 cells (*n* = 50). Scale bars, 50 μm. **j** Representative images and quantification (*n* = 3) of the colony-forming ability of shNC and shKIN KYSE30 cells treated with 5 μg/mL DDP or PBS. Statistical significance is indicated as ****P < 0.0001, ***P < 0.001, **P < 0.01, *P < 0.05

Proteins involved in the DDR are frequently recruited to or in proximity to sites of DNA damage.^7^ To verify whether KIN is actually a DDR protein, we examined whether it accumulates within the nucleus when DNA is damaged. We induced DNA damage by treating cells with DDP and observed an enrichment of KIN at the protein level (Fig. 3a and supplementary Fig. 3a). Using phosphorylated histone H2AX at serine 139 (γH2AX) as a marker of DNA damage, we incubated cells treated with DDP or PBS with a specific antibody, followed by immunofluorescence (IF) detection. A more robust accumulation of KIN was observed in DDP treated cells, particularly at sites of DNA damage (Fig. 3b, c). These results suggest that KIN is required for the DNA damage response.

To further confirm the participation of this protein in maintaining chromosome stability, we performed DNA damage detection in a KIN depletion model. A stronger phosphorylation signal at the H2AX S139 site was observed in KIN knockdown ESCC cells than in control cells (Fig. 3d and Supplementary Fig. 3b). IF analysis also revealed that more γH2AX foci accumulated in KIN-deficient cells (Fig. 3e, f). Next, we expressed Flag-KIN or an empty vector in ESCC cells to determine whether KIN expression is sufficient to support DNA stability. Indeed, cells expressing KIN presented lower levels of H2AX phosphorylation induced by DDP (Fig. 3g). Additionally, KIN depletion increased the olive tail moment (Fig. 3h, i). On the other hand, KIN deficiency resulted in vulnerability to DDP treatment (Fig. 3j and Supplementary Fig. 3c). These results indicate that KIN supports genome stability in rapidly proliferating ESCC cells.

### KIN forms a complex with DHX9

**Fig. 4:**
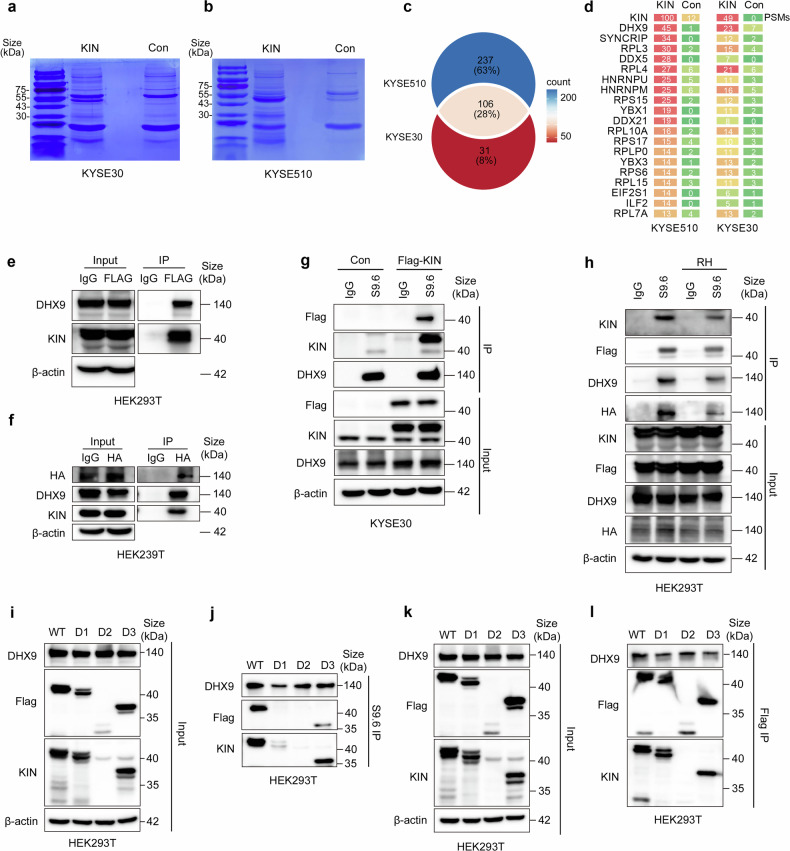
KIN forms a complex with DHX9. **a**, **b** Coomassie blue staining of proteins co-precipitated by anti-Flag beads from lysates of Flag-KIN or empty vector expressed KYSE30 (**a**) and KYSE510 (**b**). **c**, **d** Differential protein bands of the immune-precipitates from KYSE30 or KYSE510 cells extracts were analyzed by mass spectrometry (**c**). Intersected proteins were showed (**d**). **e** Protein expression in Flag co-precipitate effluent from Flag-KIN expressed HEK293T. **f** Protein expression in HA-DHX9 co-precipitate effluent from HA-DHX9 expressed HEK293T. **g** Proteins precipitated by S9.6 antibody. **h** Proteins precipitated by S9.6 antibody after addition of RNaseH. **i**, **j** S9.6 IP analysis of the association of DHX9 and KIN mutants with the R-loop in HEK293T cells. **k**, **l** Flag-IP analysis of the association of DHX9 with the indicated FLAG-tagged KIN mutants in HEK293T cells

Although KIN has been found to be related to DNA damage repair in terms of its structure, less is known about the mechanism involved. To investigate the underlying mechanism, we collected lysates from cells overexpressing Flag-KIN or an empty vector and subjected them to immunoprecipitation (IP) (Fig. 4a, b). An enrichment at ~40–50 kDa was identified as KIN, and we confirmed the enrichment of KIN in the IP effluent (Supplementary Fig. 4a). The gels were subsequently subjected to mass spectrometry (MS) to identify proteins that interact with KIN. A total of 343 KIN binding proteins were detected in effluent from KYSE510, and 137 were detected in effluent from KYSE30 (Fig. 4c). The 106 proteins that intersected in KIN binding between the two cell lines were used for further study. Most of these proteins were involved in ribosome construction or RNA processing (Fig. 4d). DHX9, the most abundant protein detected in addition to KIN, acts as an RNA helicase. The enrichment of DHX9 in the Flag-KIN IP effluent was confirmed with a specific antibody against DHX9 (Supplementary Fig. 4a). To further confirm the reliability of our MS data, we performed endogenous immunoprecipitation using samples extracted from KYSE30 cells. We detected legible signals of DHX9, DDX5, and RPS15 in the KIN-enriched samples (Supplementary Fig. 4b).

Recent studies revealed that DHX9 is an important regulator of the R-loop and controls key components of DNA damage repair, such as BRCA2 and RAD51 recruitment, after a DNA break.^7,12,26^ Thus, we hypothesized that KIN participates in the regulation of DNA damage response *via* interaction with DHX9. To validate the protein interactions identified by MS, we performed coimmunoprecipitation (co-IP) of FLAG from the lysate of HEK293T cells expressing Flag-KIN and detected its interaction with DHX9. Anti-FLAG antibodies were able to co-IP DHX9 in cells expressing Flag-KIN (Fig. 4e). Next, we performed reverse IP using HEK293T cells expressing an HA-DHX9 plasmid. Similarly, KIN was also efficiently coprecipitated with DHX9 (Fig. 4f). Taken together, these results indicate that KIN and DHX9 cooperate in the DDR.

To verify whether KIN plays a role in R-loop resolution, we performed immunoprecipitation using S9.6, a specific antibody for the R-loop. We found that KIN and DHX9 could be effectively immunoprecipitated by the R-loop structure (Fig. 4g). This interaction was reduced after the addition of RNaseH, a specific enzyme that targets the R-loop (Fig. 4h and Supplementary Fig. 4c). To further investigate the necessity of R-loops for the interaction between KIN and DHX9, we conducted immunoprecipitation experiments in the presence or absence of RNaseH in the reaction buffer. Indeed, neither KIN nor DHX9 could effectively coprecipitate with each other in the absence of R-loops (Supplementary Fig. 4d).

KIN is composed of three main domains: 1) the N-terminal zinc finger domain; 2) the middle wHTH domain; and 3) the C-terminal SH3-like domain. To investigate the underlying mechanism by which KIN binds to the R-loop, we expressed FLAG-tagged WT KIN or three deletion mutants in HEK293T cells (Fig. 4i). After DDP treatment, we harvested the cells for S9.6 immunoprecipitation. Helix domain depletion seems to affect KIN stability. The N-terminal zinc finger domain is a typical DNA-binding domain, the depletion of which disturbs the interaction of KIN with the R-loop (Fig. 4j). The C-terminal SH3-like domain is thought to be the RNA-binding domain, the depletion of which also disturbs the interaction of KIN with the R-loop (Fig. 4j). These findings indicate that KIN binds to the R-loop through the nucleus-binding domain, especially the N-terminal DNA binding domain. Next, we performed Co-IP using Flag tagged magnetic beads and observed that the DNA binding domain is the most important part of the interaction between KIN and DHX9 (Fig. 4k, l). Taken together, these data indicate that KIN plays a critical role in R-loop regulation, acting in concert with DHX9.

### KIN supports the recruitment of DHX9 to R-loop sites

**Fig. 5:**
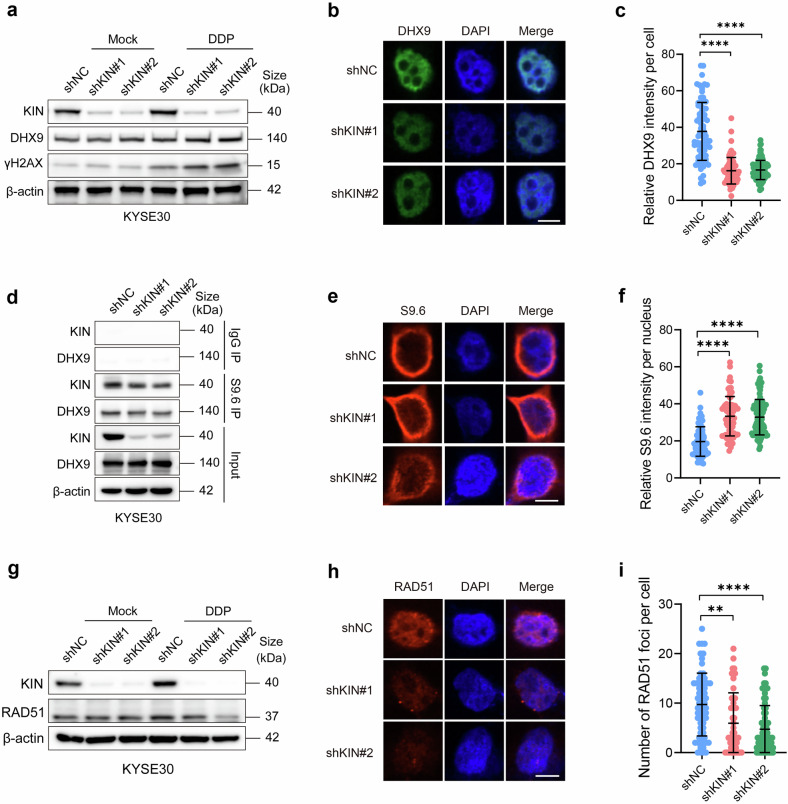
KIN recruits DHX9 to R-loop site. **a** Protein expression in shNC and shKIN KYSE30 cells treated with PBS or 5 μg/ml DDP for 2 h. **b**, **c** Fluorescence image (**b**) and quantification (**c**) of DHX9 in shNC and shKIN KYSE30 cells treated with 5 μg/ml DDP for 2 h (*n* > 50). Scale bars, 10 μm. **d** Proteins precipitated by S9.6 antibody in shNC and shKIN KYSE30. **e**, **f** Fluorescence image (**e**) and quantification (**f**) of R-loops in shNC and shKIN KYSE30 cells (*n* > 50). Scale bars, 10 μm. **g** Protein expression in shNC and shKIN KYSE30 cells treated with PBS or 5 μg/ml DDP for 2 h. **h**, **i** Fluorescence image (**h**) and quantification (**i**) of RAD51 in shNC and shKIN KYSE 30 cells treated with 5 μg/ml DDP for 2 h (*n* > 50). Scale bars, 10 μm. Statistical significance is indicated as ****P < 0.0001, ***P < 0.001, **P < 0.01, *P < 0.05

DNA damage is a driving force of R-loop formation (Supplementary Fig. 5a-b), R-loop resolution requires specific RNA helicases such as DHX9, and certain nuclear binding proteins appear to be essential for proper recruitment of these helicases.^7,27^ We hypothesized that KIN also functions as a recruiter of RNA helicases after DNA damage-induced R-loop formation. To validate our hypothesis, we characterized DHX9 in KIN-knockdown cells. We observed an accumulation of DHX9 in the nucleus after DDP-induced DNA damage (Supplementary Fig. 5c–e), suggesting that DHX9 is required for the DNA damage response in ESCC. While the total protein level was not significantly affected after KIN knockdown (Fig. 5a), the intranuclear intensity of DHX9 decreased in KIN-depleted cells (Fig. 5b, c). Next, we performed S9.6 IP in KIN-knockdown or control cells and detected a decrease in R-loop binding to DHX9 (Fig. 5d). Additionally, we observed an increase in S9.6 intranuclear immunofluorescence signal in KIN-depletion cells (Fig. 5e, f). To further detect R-loop accumulation in cells, we applied DRIP^14^ to shNC and shKIN KYSE30 cells and an observed increased fluorescence signal in KIN depleted cells, and these signals decreased after RNase H treatment (Supplementary Fig. 6). These results indicate that KIN is important for DHX9 recruitment to R-loop sites and that depletion of KIN results in R-loop accumulation.

To repair damaged DNA, the repair factor RAD51 accumulates at the damage site and recruits additional repair factors^7^(Supplementary Fig. 5f–h). Given that abnormal R-loop resolution is crucial for downstream DNA repair complex recruitment,^7^ we speculated that genome instability associated with KIN depletion may be attributed to a failure in the recruitment of these repair complexes. Indeed, RAD51 was not enriched in cells after KIN depletion in response to DNA damage (Fig. 5g). Similarly, IF detection revealed decreased intranuclear accumulation of RAD51in KIN-depleted cells after DNA damage (Fig. 5h, i). Collectively, these results indicate that KIN supports the resolution of the R-loop and triggers DNA repair.

### Defect of KIN induces an interferon response *via* NFκB

**Fig. 6:**
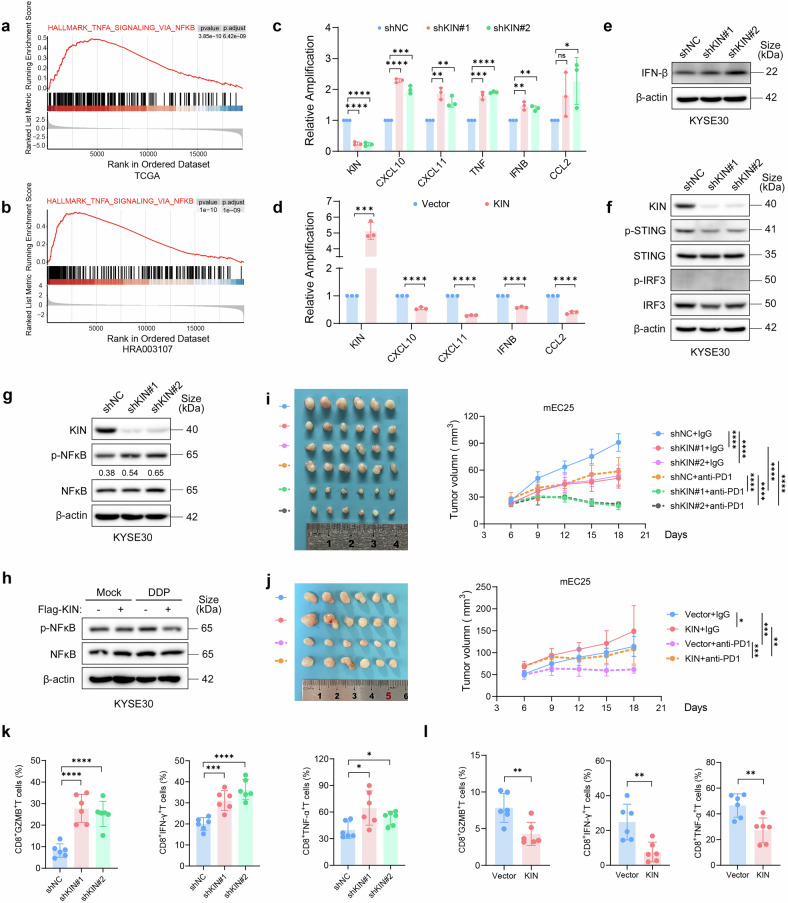
Defect of KIN induces interferon response *via* NFκB. **a**, **b** GSEA comparing the gene expression profiles between KIN high and low group, data from TCGA (**a**) and data from HRA003107 (**b**). **c** Differentially expressed cytokine/chemokine in shNC and shKIN KYSE30 cells (*n* = 3). **d** Differentially expressed cytokine/chemokine in KYSE30 cells expressed KIN or empty vector (*n* = 3). **e** IFN-β protein expression in shNC and shKIN KYSE30 cells. **f** STING activation *via* IRF3 detected by WB in shNC and shKIN KYSE30 cells. **g** STING activation *via* NFκB detected by WB in shNC and shKIN KYSE30 cells. Grey values of p-NFκB were showed under the band of p-NFκB. **h** STING activation *via* NFκB detected by WB in KYSE30 cells expressed KIN or empty vector treated with 5 μg/ml DDP for 2 h. **i** Tumor growth curves and representative image of shNC and shKIN mEC25 tumors treated with anti-PD-1 antibody or isotype (*n* = 6). **j** Tumor growth curves and representative image of mEC25 tumors expressing KIN or empty vector treated with anti-PD-1 antibody or isotype (*n* = 6). **k** Percentages of infiltrating CD8^+^ GZMB^+^T cells, CD8^+^ IFN-γ^+^ T cells and CD8^+^TNF-α^+^ T cells in shNC and shKIN mEC25 tumors (*n* = 6) were analyzed by flow cytometry. **l** Percentages of infiltrating CD8^+^GZMB^+^T cells, CD8^+^IFN-γ^+^T cells and CD8^+^TNF-α^+^T cells in mEC25 tumors expressing KIN or empty vector (*n* = 6) were analyzed by flow cytometry. Statistical significance is indicated as ****P < 0.0001, ***P < 0.001, **P < 0.01, *P < 0.05

**Fig. 7:**
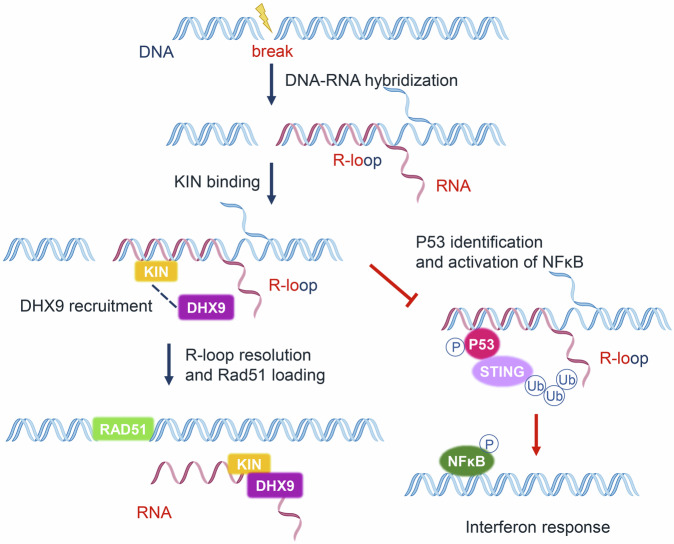
Schematic of KIN mediated R-loop regulation and inhibition of STING activation

To investigate the biological impact of enriched KIN in ESCC, we performed GSEA of DEGs between the KIN-high group and the KIN-low group in both the TCGA ESCA dataset and the HRA003107 dataset, which revealed enrichment of TNFA signaling *via* NFKB (Fig. 6a, b). To validate the stimulation of innate immune factors, we performed qRT-PCR on samples extracted from KIN-knockdown cells or control cells. Multiple interferon-stimulated genes (ISGs), including CXCL10, CCL2, CXCL11, and IFNB, as well as the nuclear factor kappa B (NF-κB)-responsive gene (TNF), were induced after KIN knockdown (Fig. 6c). In contrast, these genes presented decreased expression in KIN-overexpressed cells (Fig. 6d). We also detected an increased protein levels of IFN-β in KIN knockdown cells after DNA damage (Fig. 6e and supplemantary Fig. 7a). These results indicate that a low level of KIN in ESCC is related to the activation of the innate immune system.

Given that KIN also plays an important role in R-loop regulation, we propose that KIN deficiency-induced R-loop aberration and DNA damage are correlated with activated TNF signaling. Unlike previous studies, depletion of KIN had a weak effect on the phosphorylation of STING and IRF3, the canonical factors activated in the cGAS-STING pathway (Fig. 6f). Even so, a recent study revealed noncanonical activation of STING by abnormal nucleic acids within the nucleus, which typically ubiquitinates STING and activates NFκB rather than IRF3.^28^ Unlike the canonical activation of STING, the depletion of KIN induced a significant increase in the phosphorylation level of NFκB (Fig. 6g). Corresponding manifestations also appeared in KIN-overexpressing cells (Fig. 6h and supplementary Fig. 7b). These results indicate that KIN deficiency-induced R-loops contribut to the activation of the innate immune response *via* NFκB in ESCC.

To elucidate the effect of the innate immune response in vivo, we constructed an allograft tumor model in immune competent mice *via* KIN-knockdown mEC25 cells and control mEC25 cells. We observed a decreased tumor growth in KIN-knockdown tumors, and depletion of KIN improved the therapeutic effects of the anti-PD-1 antibody (Fig. 6i and Supplementary Fig. 7g). In contrast, the overexpression of KIN (Supplementary Fig. 7c, d) improved tumor growth and inhibited the therapeutic effects of the anti-PD-1 antibody (Fig. 6j, Supplementary Fig. 7e, f and Supplementary Fig. 7h, i). Then, we applied flow cytometry to extracted tumor tissues and observed increased cytokine expression in CD8^+^ T cells from KIN knockdown tumors (Fig. 6k and Supplementary Fig. 7j). In addition, cytokine expression in CD8^+^ T cells decreased in KIN-overexpressed tumors (Fig. 6l). Collectively, these results indicate that KIN loss in ESCC tumors induces a robust antitumor immunity, which may ultimately improve ICB treatment outcomes.

## Discussion

In this work, we demonstrated that enriched DDR genes support ESCC progression and affect the tumor immune microenvironment. KIN, a key DDR gene, was upregulated in ESCC. This high level of KIN supports ESCC genome stability and facilitates escape from apoptosis. We found that KIN, a new R-loop binding protein, interacts with the RNA helicase DHX9 and demonstrated that this interaction plays an important role in addressing the R-loop associated with DNA damage. Our findings suggest that KIN is part of the R-loop resolution complex and that it supports the recruitment of DHX9. This regulatory effect on the R-loop supports the escape of ESCC cells from the IFN response induced by DDP. (Fig. 7).

Genome instability is one of the hallmarks of cancer, and is characterized by a highly potent of gene mutations that support tumorigenesis.^2^ This deficiency is thought to be one of the factors leading to gene mutations in tumors. Cells with deficiencies in certain DDR genes tend to rely on alternative compensatory DDR pathways, leading to the activation of specific DDR genes in certain types of cancer. DDR genes are important for the antitumor immune response and immunoreactivity of certain cancers.^29^ DDR genes have been used to predict the immune status of patients with cancer.^30^ However, the value of highly expressed DDR genes in ESCC remains to be investigated. We divided patients into two groups on the basis of their DDR scores and compared the immune profiles between the two groups. Samples with low DDR scores are vulnerable to DNA damage induced by endogenous or external factors, which leads to the accumulation of abnormal nucleic acids and genomic instability. These abnormal nucleic acids are important sources of neoantigens and induce the upregulation of immune checkpoint expression, thereby altering the immune balance in the TME. Therefore, investigating the contribution of DDR genes to tumor immune efficacy and the role of activated DDR genes in ESCC is crucial. We identified a key differentially expressed protein, KIN, between ESCC tumor tissues and normal tissues that may play an important role in ESCC progression. KIN has long been predicted to be correlated with DNA damage repair.^31,32^ However, the mechanism by which KIN participates in the DDR remains unclear. Previous interactome studies failed to identify any classical DDR complex but did identify many RNA-processing proteins.^33^ Consistently, our KIN interactome study also revealed many RNA processing proteins but not classical DDR proteins. Nevertheless, our study revealed an interaction between KIN and the R-loop processing protein DHX9, and this interaction inspired us to investigate the nonclassical role of KIN in the DDR.^34^ Recent studies have demonstrated that R-loops formed at DNA damage sites can impede the binding of DNA damage repair proteins to damaged nucleic acids.^35^ The RNA helicase DHX9 participates in the DNA damage response by eliminating R-loops. Additionally, nucleic acid-binding proteins such as ARID1A play crucial roles in the process of R-loop clearance mediated by RNA helicases.^8^ Deficient KIN diminishes DHX9 recruitment to the R-loop. Recently, RNA helicases have been found to act as R-loop resolvers, contributing to DNA damage repair.^36,37^ We hypothesize that KIN supports the recruitment of the R-loop resolution complex. Importantly, the accumulation of R-loops resulting from KIN deficiency can activate the innate immune response in ESCC cells and improve the tumor immune microenvironment. Depletion of KIN enhances the efficacy of both chemotherapy and immunotherapy in a mouse model. Our study provides favorable evidence and a mechanistic foundation for further clinical applications.

We also note that some limitations still exist in our research. Although we systematically investigated the function of KIN in supporting R-loop clearance by DHX9, we did not identify the specific site of KIN that binds to DHX9. Our research focused on the molecular mechanism by which activated DNA damage response proteins support ESCC progression, and most results are derived from cell lines and mouse models but lack insight into clinically relevant data from patient-derived systems. To investigate the role of KIN in affecting the immune system, we applied flow cytometry to assess cytokine expression in CD8^+^ T cells, but we did not describe the comprehensive tumor microenvironment profile.

Recently, aberrant accumulation of R-loops has been linked to STING pathway activation and the interferon response.^14,38^ Researchers have demonstrated that deficiencies in R-loop resolution cause the release of R-loops into the cytoplasm. These abnormally accumulated nucleic acids in the nucleus trigger the activation of interferon-induced apoptosis, which may exacerbate inflammation but also increase antitumor immune activity. Instead of IRF3 activation through c-GAS *via* phosphorylated STING, we established the activation of noncanonical STING activation *via* NFκB in an R-loop processing-deficient manner. Before excessive R-loops are released into the cytoplasm, aberrant nucleic acid accumulation in the nucleus is sufficient to activate sensors within the nucleus and stimulate the STING pathway *via* NFκB. These findings may provide new insights into DNA damage related innate immune activation. We first connected KIN, a ubiquitous protein in proliferating cells, with this innate immune activation, thereby expanding the potential functions of KIN. These results indicate that KIN is a promising target for ESCC treatment, and further studies are needed to determine the actual effect of immunotherapy with a KIN-targeting strategy.

In summary, our study demonstrates the value of DDR genes in ESCC. High levels of DDR gene expression endow ESCC with the ability to resist the threat of genomic damage caused by rapid proliferation, thereby maintaining genomic stability. By comparing the proteomic differences between tumor tissues and normal tissues, we identified the key DDR protein KIN in ESCC, which has not been adequately studied. Our interactome data indicate that KIN is not a component of classical DNA damage response proteins but functions with DHX9. We first revealed that KIN is an R-loop-binding protein that participates in genome maintenance through supporting the recruitment of the RNA helicase DHX9 to R-loop sites. We show that KIN is a crucial suppressor of R-loop accumulation and that genetic perturbation of KIN leads to DNA damage as well as activation of the innate immune response. Furthermore, we found that DNA-damage induced R-loops in the nucleus can activate noncanonical STING *via* NFκB rather than IRF3, and we highlight KIN as a potential novel target for enhancing cancer immunotherapy in ESCC.

## Materials and methods

### Calculation of the DDR score, immune Score, and gene set enrichment analysis

The DNA repair gene set originated from the Reactome Pathway database (www.reactome.org, ID: R-HSA-73894). GSVA-derived DDR scores in 155 ESCC RNA datasets (HRA003107) were calculated *via* R. Based on median DDR score values, 155 patient samples were divided into DDR-high and DDR-low groups for subsequent analysis. Differentially expressed genes (DEGs) between the DDR-high and DDR-low groups were identified *via* the limma package in R (Supplementary Table 2). The limma package in R was used to DEGs between the DDR-high and DDR-low groups (adjusted *p*-value < 0.05 and cutoff |log2(FC) | = 1.0). GSEA analysis was further carried out for hallmark gene sets and other gene sets from canonical pathways curated in the mSigDB database. Reactome pathway enrichment was further carried out *via* the clusterProfiler package in R. The ImmuneScore, StromalScore, and ESTIMATEScore were outputted *via* ESTIMATE algorithm.^39^ We performed gene-set enrichment analysis of the HRA003107 ESCC dataset^23^ and TCGA dataset *via* GSEA (R implementation). 28 immune cell subpopulations gene sets^24^ were included in the immune infiltration analysis. Statistical significance was determined *via* Wilcoxon signed rank test.

### Antibodies and reagents

The sources of antibodies against the following proteins or post-translational modifications were used: KIN (12313-1-AP), DHX9 (17721-1-AP), RAD51 (14961-1-AP), HA-tag (81290-1-RR), and Flag-tag (80010-1-RR) from Proteintech. p-γH2AX (#97148), p-STING (#50907), STING (#13647), p-NFκB (#3033), NFκB (#8242), p-IRF3 (#29047), and IRF3 (#11904) from CST, S9.6 (ENH001) from Kerafast.

### Plasmids

KIN cDNAs were chemically synthesized by You Bio and cloned into the pLenti6B-3 × FLAG-Puro vector. DHX9 cDNA clones were subcloned into the pLVX-puro plasmid. Nontargeting oligo control or shRNA oligos targeting KIN were engineered into pSIH1-puro plasmid.

We used pMD2.G and pSPAX2 for lentivirus packaging.

### Cell culture

The human ESCC cell lines KYSE510 and KYSE30 were generously provided by Dr. Y. Shimada (Kyoto University, Kyoto, Japan). The human ESCC cell lines were maintained in RPMI 1640 supplemented with 10% fetal bovine serum (FBS) (HyClone, South Logan, UT, USA).

The mouse ESCC cell line HNM007 was kindly provided by Dr. Anil Rustgi from Columbia University. The mouse ESCC cell line mEC25 was kindly provided by Dr. Li Fu from Shenzhen University School of Medicine. Both of them were cultured in DMEM supplemented with 10% FBS.

The HEK293T cell line was purchased from the American Type Culture Collection (ATCC) (Manassas, VA, USA). HEK293T cells were maintained in DMEM supplemented with 10% FBS (HyClone, South Logan, UT, USA).

All cells were routinely subjected to short tandem repeat analysis and regularly tested for mycoplasma contamination.

### Animal study

All procedures and experimental protocols were approved by the Institutional Animal Care and Use Committee of Chinese Academy of Medical Sciences Cancer Hospital. KYSE30, mEC25, and HNM007 cells transfected with indicated virus were subcutaneously implanted in mice. Mice were assigned to different groups randomly for further treatment when most tumors grew approximately 5 mm in diameter. For DDP treatment, mice were intraperitoneally injected with 5 mg/kg DDP or PBS every three days. For anti-PD-1 antibody treatment, mice were intraperitoneally injected with 5 mg/kg anti-PD-1 antibody (clone RMP1-14, Bio X Cell, BE0146) or isotype (rat IgG2a, clone 2A3, Bio X Cell, BE0089) every three days. Mice cages were placed next to each other. Mice were asphyxiated by carbon dioxide while the study reached the endpoint.

### Western blot

Western blot analysis was conducted following the standard protocol. In brief, cells were collected and lysed in RIPA buffer (1× proteinase inhibitor cocktail (Roche), 0.5% sodium deoxycholate, 50 mM Tris–HCl (pH 7.4), 150 mM NaCl, 1 mM EDTA, 0.1% sodium dodecyl sulfate (SDS), and 1% NP-40). The lysis process was carried out for 30 min on ice.

The samples were separated by SDS-PAGE followed by transferring onto PVDF membranes (Millipore). The membranes were incubated with a 5% milk powder solution for block. The blocked membranes were incubated with specific primary antibodies overnight at 4 °C. The membranes were incubated with corresponding secondary antibodies. The ImageQuant LAS - 4000 System (GE) is used to visualized immunoblots.

### RNA extraction and qRT-PCR

Total RNA was extracted using TRIzol reagent (Thermo Fisher Scientific). cDNA synthesis was carried out with the Quantscript RT Kit (Tiangen, Beijing, China), in accordance with the manufacturer’s instructions. Real-time reverse transcription-polymerase chain reaction (RT-PCR) was performed on a StepOnePlus Real-Time PCR System (Applied Biosystems, Foster City, CA, USA) using SYBR Premix Ex Taq™ II (TaKaRa, Japan), in accordance with the manufacturer’s instructions. The primers used in this study are listed in Supplementary Table 1.

### Cell proliferation assay

Cell proliferation was assessed using the Cell Counting Kit-8 (CCK-8) assay. 2 × 10^3^ cells were seeded into each well of 96-well plates. After incubation, remove the supernatant and add CCK-8 solution. Following a 1 h incubation period at 37 °C, the absorbance was measured using a microplate reader (BioTek) at 450 nm.

### Colony-forming unit assay

1 × 10^3^ cells were seeded into each well of 6-well plates. Cells were incubated for 10–14 days to allow colony formation.

### Co-immunoprecipitation

For immunoprecipitation (IP), 1500 μg of total protein was incubated with 50 μL of protein A/G magnetic beads that had been pre-incubated with the primary antibody or Flag-tag magnetic beads (MCE) overnight at 4 °C. Subsequently, the beads were washed five times using TBST buffer. The beads were collected at 4 °C using a magnetic stand (MCE). The samples were incubated in 2× SDS-PAGE loading buffer and boiled for 10 min. The boiled samples were then separated by SDS-PAGE followed by transferring onto PVDF membranes (Millipore). The MS analysis was performed by PTM Biolab.

### Immunofluorescence

Cells were seeded onto glass coverslips (BD Biosciences). After incubation, cells were fixed with 4% paraformaldehyde, followed by permeabilizing in 0.2% Triton X-100 in PBS. The samples were then blocked in 5% bovine serum albumin (BSA) and 0.1% Triton X-100 in PBS. Cells were stained with the appropriate primary and secondary antibodies conjugated to Alexa Fluor 488 or 594 (Invitrogen). Images of both regular scan areas and broader regions were captured using a laser confocal microscope.

### Comet assay

The Comet Assay kit (R&D Systems) was utilized to monitor DNA damage according to the manufacturer’s instructions. In brief, cells were resuspended at a concentration of 1 × 10⁶ cells/mL in pre-chilled PBS. Mix 50 μL of warm low-melting agarose with 5 μL samples and spread the mixture onto comet slides evenly. Place slides in the dark for 10 min at 4 °C and incubate in a pre-chilled lysis solution at 4 °C for 60 min. The slides were incubated at room temperature in Alkaline Unwinding Solution (pH > 13) for 20 min. Electrophoresis was performed at 1 volt/cm and 300 mA at 4 °C. Immerse the slide in deionized water 5 min two times and washed in 70% ethanol 5 min. The samples were incubated with 100 μL of propidium iodide for 20 min in the dark and dried. Prepared samples were visualized using an inverted fluorescence microscope. The Olive tail moment was calculated using the formula: (Tail Mean − Head Mean) × (% of DNA in the Tail).

### DRIP assay

Genomic DNA was incubated with or without RNase H at 4 °C overnight following sonication. For immunoprecipitation, 16 μg of the S9.6 antibody was incubated with magnetic protein A/G beads (MCE) in 1× binding buffer (1% Triton X-100; 150 mM NaCl; 20 mM Tris-HCl, pH 8.0; 0.5% sodium deoxycholate; 2 mM EDTA) at 4 °C for 4 h. Samples were incubated with prepared S9.6 coated beads at 4 °C overnight. After incubation, the beads were washed using TSE buffer (1% Triton X-100; 20 mM Tris-HCl, pH 8.0; 2 mM EDTA; 150 mM NaCl; 0.1% SDS) follow by TE buffer. Samples were eluted using elution buffer (0.5% SDS; 10 mM EDTA; 8 μL of proteinase K at 20 mg/mL; 50 mM Tris, pH 8.0) at 50 °C for 50 min. For fluorescence detection, the eluted samples were mixed with loading buffer and separated on 6% TBE gels. The images were captured using a gel imager with UV illumination.

### Flow cytometry analysis

To evaluate the status of immune cell infiltration in mouse tumors, single-cell suspensions were prepared from fresh harvested tumor tissue. Tumor tissues were dissected, followed by digestion in 5 mg/mL collagenase Type I (17100017; Thermo Fisher) at 37 °C for 30 min. This was followed by a second digestion step using 1 mg/mL collagenase Type IV with 20 μg/mL DNase. To remove clumps and maintain single-cell integrity, the cell suspension was passed through a 70 µm filter. The single-cell suspensions were incubated with anti-CD16/CD32 antibodies (Tonbo, 70-0161-M001) for 15 min at room temperature for block. Next, the samples were incubated with BD Horizon™ Fixable Viability Stain 700 (BD, 564997) for 15 min. Subsequently, the samples were incubated with anti-CD45 (BioLegend, 103137), anti-CD3 (BioLegend, 981002), anti-CD4 (BioLegend, 100422), anti-CD8 (BioLegend, 100734), and anti-CD19 (BioLegend, 115574) antibodies at 4 °C for 20 min. The samples were then fixed in fixation buffer and incubated with anti-GZMB (BioLegend, 372216), anti-IFN-γ (BioLegend, 505810), and anti-TNF-α (BioLegend, 502909) antibodies at 4 °C for 30 min.

All samples were run on the Cytek NL-CLC3000 full-spectrum flow cytometry system, and the data were analyzed using FlowJo (v.10).

### Statistical analysis

Data analysis was conducted using R project. Statistical significance was determined by using Wilcoxon signed rank test or two-sided Student’s *t*-test. ANOVA test was performed in multiple experimental groups. For data analysis in TCGA and HRA003107 datasets, the mRNA expression of indicated genes in normal esophageal mucosa tissues is from the GTEx dataset. For the functional experiment in vitro, experiments were performed independently at least three times. The data were reported as mean ± S.D. *P*-value < 0.05 was considered statistically significant.

## Supplementary information

Supplementary_Materials

The raw data of Western blot

The raw data of colony

Supplementary table 1

Supplementary table 2

## Data Availability

The ESCC RNA-seq data used in this study are publicly available from the Genome Sequence Archive (GSA) database in the BIG Data Center (http://bigd.big.ac.cn/gsa) with the BioProject number PRJCA004501. The proteome data is derived from the integrated proteome resource iProX (https://iprox.cn) with the Project accession IPX0002466000. Data supporting this study are within the manuscript and supplementary materials.
