# Supplementary material for: DNA/RNA-binding protein KIN17 supports esophageal cancer progression via resolving noncanonical STING activation induced by R-loop
Source: Signal Transduct Target Ther. 2025 Aug 15;10:256. doi: 10.1038/s41392-025-02344-2 (PMC12354822; doi:10.1038/s41392-025-02344-2)
Supplement: Supplementary file 1 — Supplementary_Materials [file 41392_2025_2344_MOESM1_ESM.docx]

Supplementary Materials for

**DNA/RNA-binding protein KIN17 supports esophageal cancer progression *via* resolving noncanonical STING activation induced by R-loop**

Zichao Wei^1#^, Ning Zhao^1#^, Lu Kuang^1^, Ji Cong^1^, Sujuan Zheng^1^, Yi Li^1*^, Zhihua Liu^12*^

* Correspondence should be addressed to

Zhihua Liu, liuzh@cicams.ac.cn; Yi Li, liyi@cicams.ac.cn

**This PDF file includes:**

Figures. S1 to S7

**
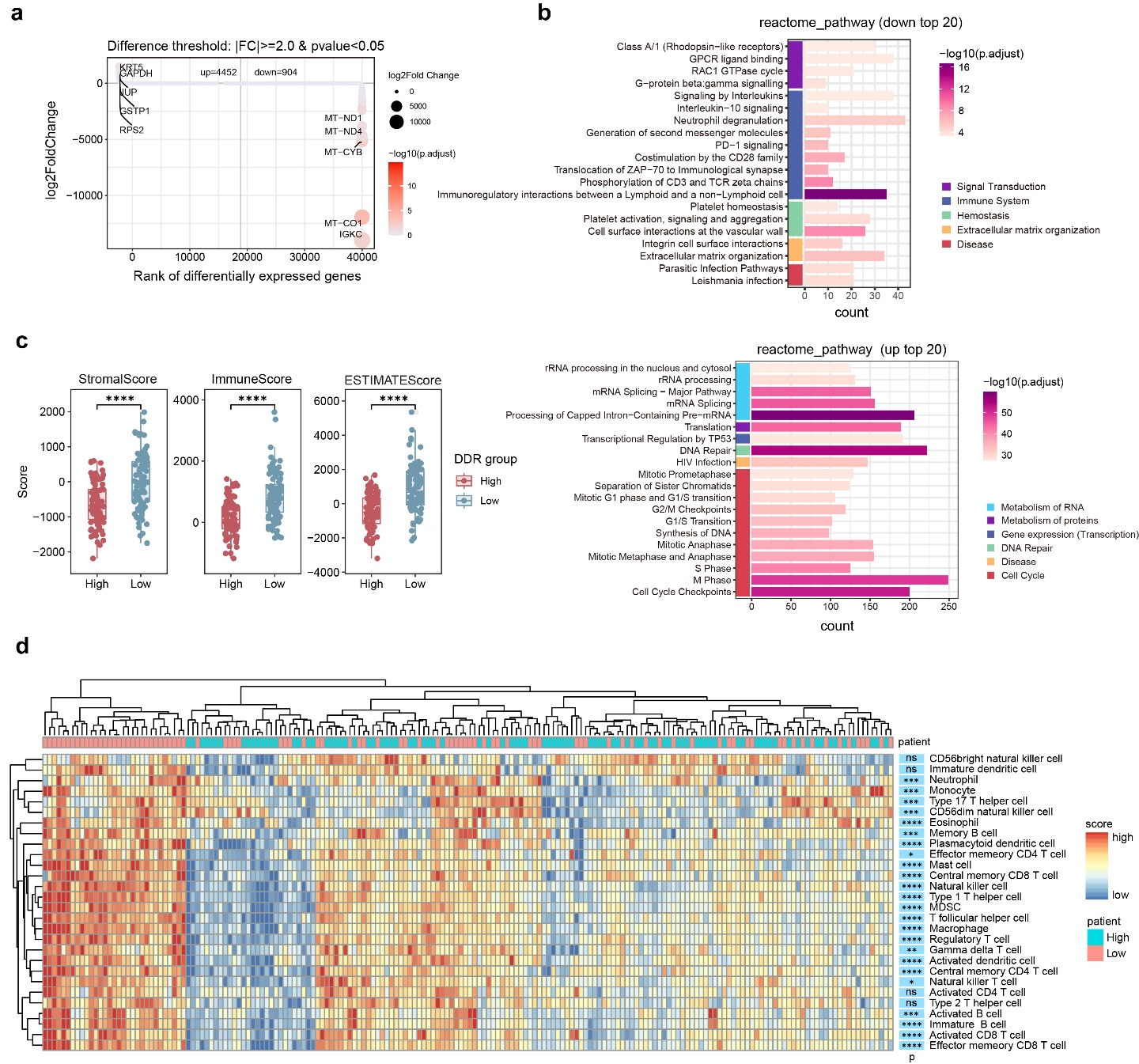
**

Figure. S1. High expression of KIN in esophageal squamous cell carcinoma. (a) Log2FC of differentially expressed genes between DDR-high group and DDR-low group using ESCA data from TCGA dataset (DDR-high group verses DDR-low group). (b) Reactome pathway analysis of differentially expressed genes between DDR-high group and DDR-low group using ESCA data from TCGA dataset. (c) ESTIMATE score in DDR dependent subgroup. Red represents DDR-high group and blue represents DDR-low group using ESCA data from TCGA dataset. (d) Heatmap shows distribution of subtypes of immune cells using ESCA data from TCGA dataset. Statistical significance is indicated as ****P < 0.0001, ***P < 0.001, **P < 0.01, *P < 0.05.


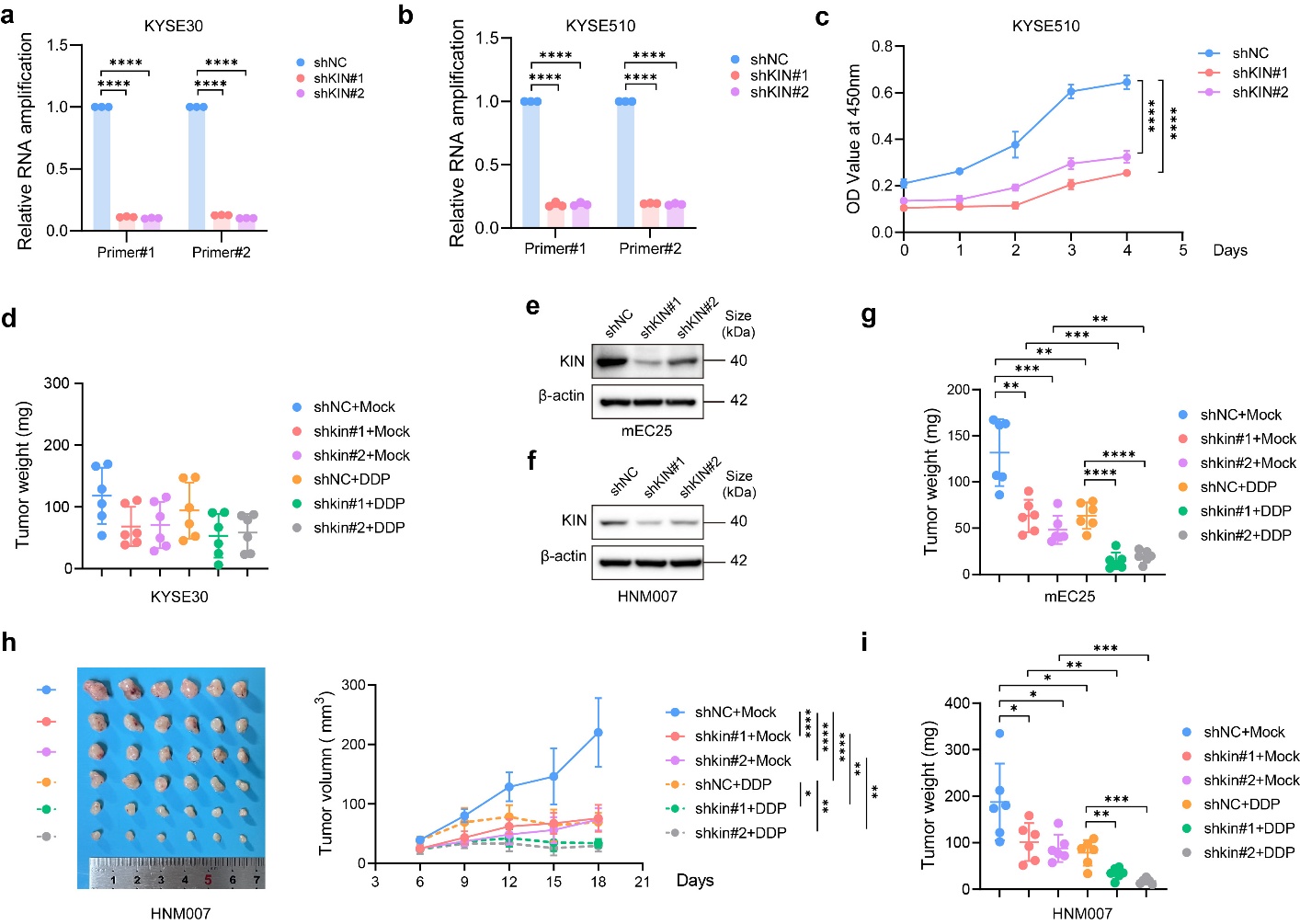


Figure. S2. KIN supports esophageal squamous cell carcinoma progression. (a-b) RNA expression of KIN in shNC and shKIN KYSE30 cells (a) or KYSE510 cells (b). (c) Growth curves of KYSE510 cell line transfected with shNC or shKIN virus. (d) Tumor weight of shNC and shKIN KYSE30 tumors with indicated treatment (n=6). (e) KIN protein expression in mEC25 cells transfected with shNC or shKIN virus. (f) KIN protein expression in HNM007 cells transfected with shNC or shKIN virus. (g) Tumor weight of shNC and shKIN mEC25 tumors with indicated treatment (n=6). (h) Tumor growth curves and representative image of shNC and shKIN HNM007 tumors treated with 5mg/kg/3Days DDP or PBS (n=6). (i) Tumor weight of shNC and shKIN HNM007 tumors with indicated treatment (n=6). Statistical significance is indicated as ****P < 0.0001, ***P < 0.001, **P < 0.01, *P < 0.05.


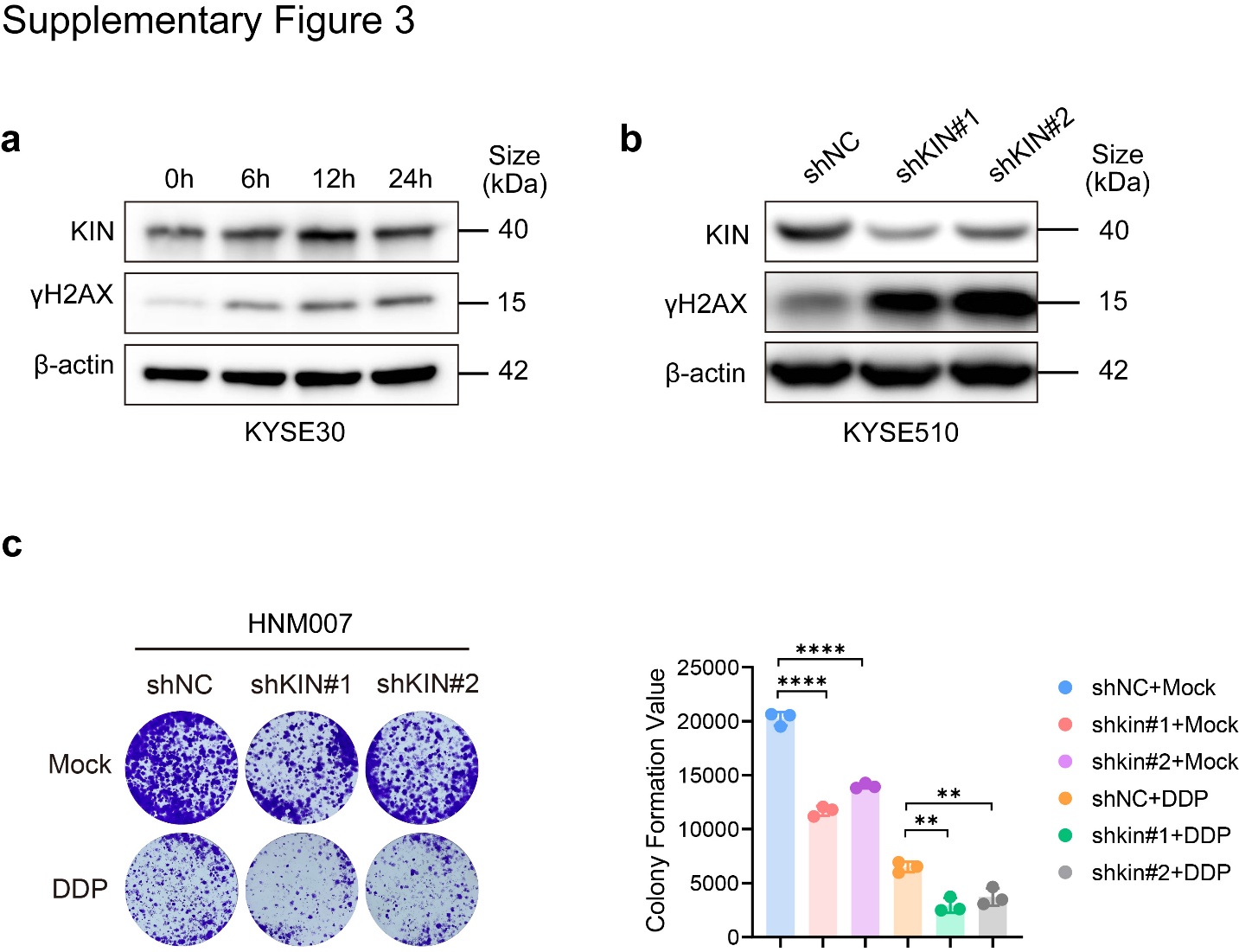


Figure. S3. KIN profile under treatment of cisplatin. (a) Protein expression in KYSE30 cells treated with 5μg/mL DDP. (b) Protein expression in shNC and shKIN KYSE510 cells. (c) Representative images (left) and quantification (right) of the colony-forming ability of HNM007 cells transfected with shNC or shKIN virus (n=3). Statistical significance is indicated as ****P < 0.0001, ***P < 0.001, **P < 0.01, *P < 0.05.


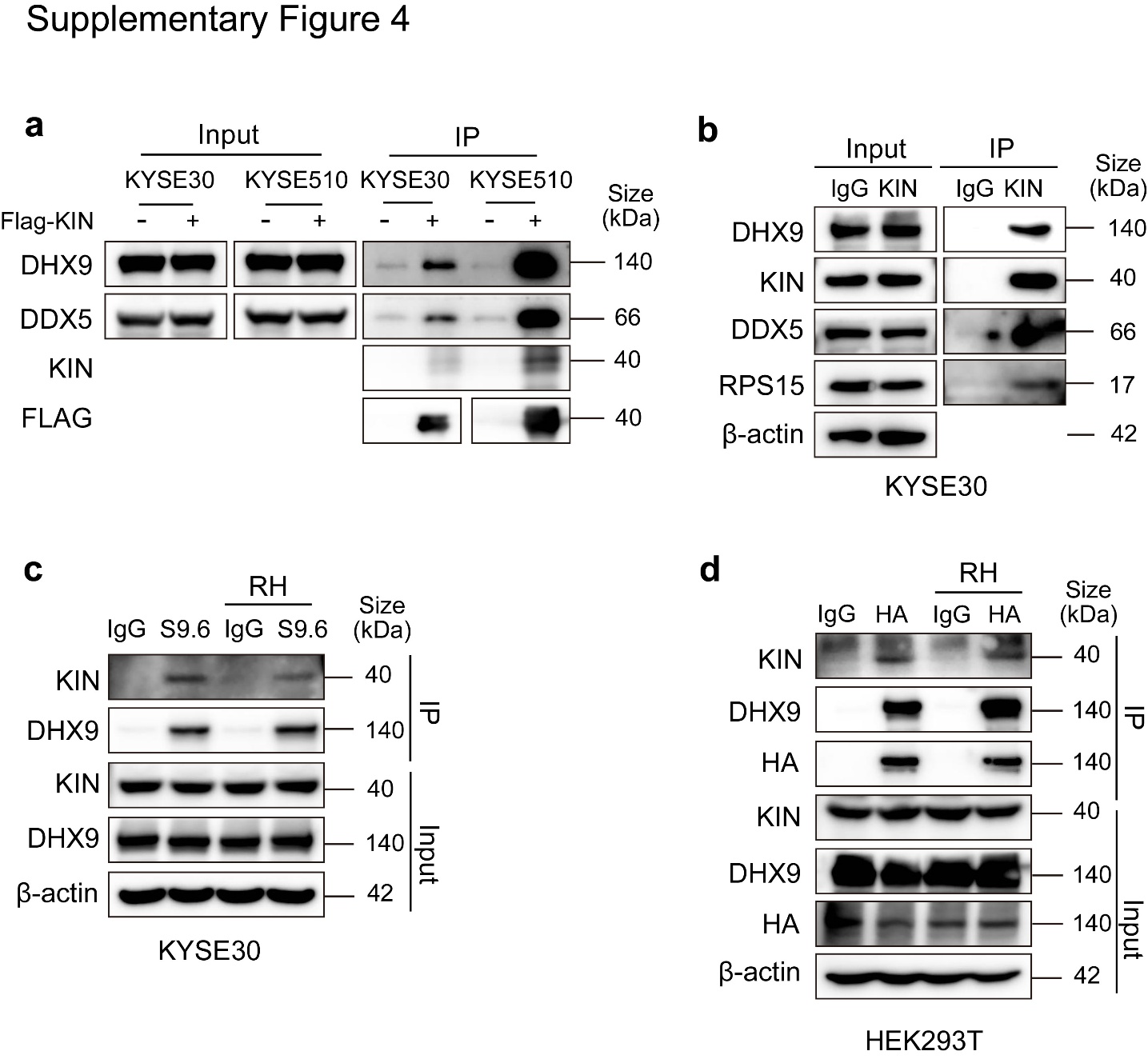


Figure. S4. Interaction between KIN and DHX9 requires R-loop. (a) Protein expression in co-precipitate effluent from Flag-KIN expressed KYSE30 or KYSE510. (b) Protein expression in co-precipitate using IgG or KIN coated beads effluent from KYSE30 cells. (c) Protein expression in S9.6 co-precipitate effluent from KYSE30. (d) Protein expression in HA co-precipitate effluent from HA-DHX9 expressed HEK293T after addition of RNaseH.


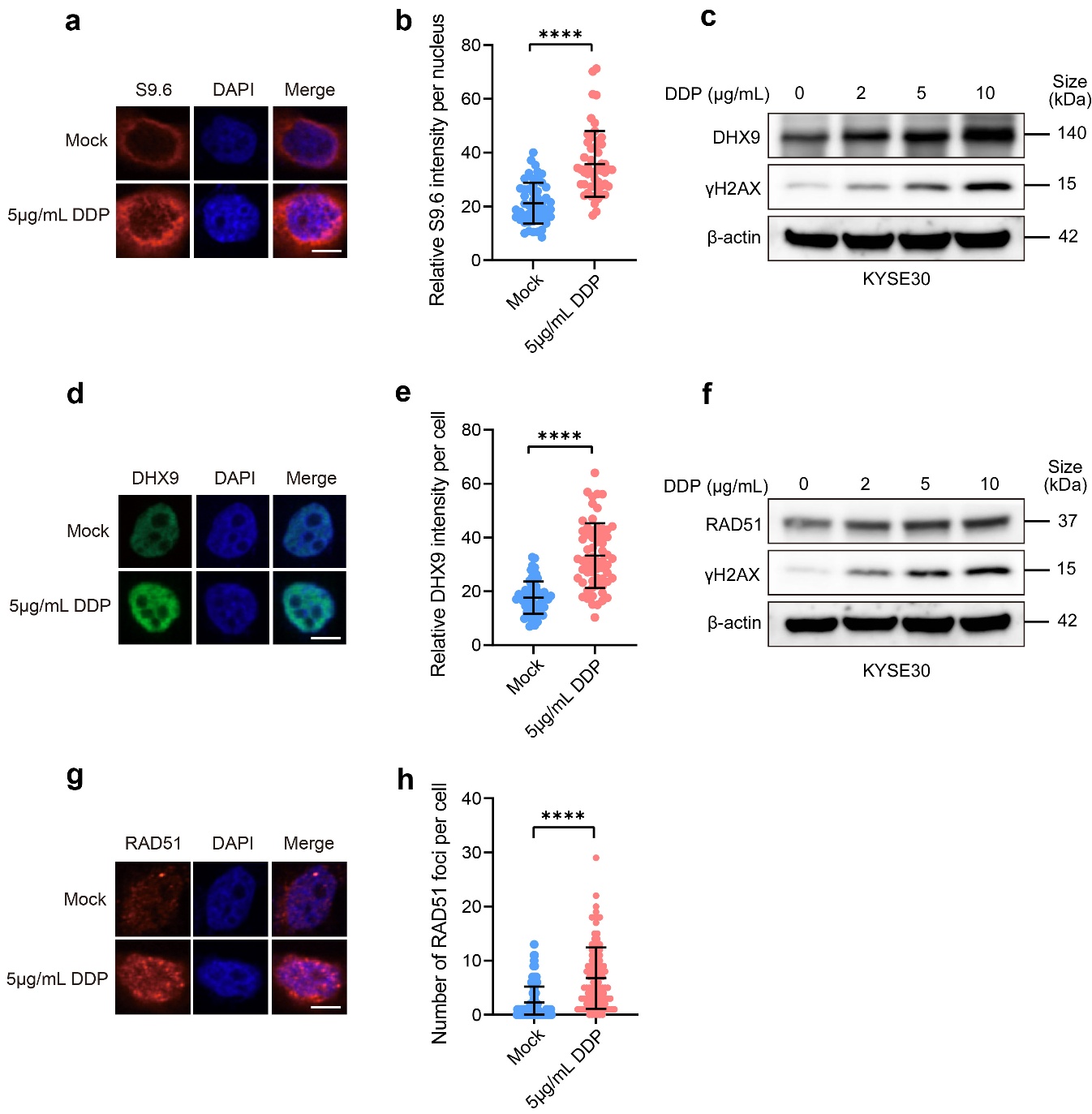


Figure. S5. Protein profile under treatment of cisplatin. (a-b) Fluorescence image (a) and quantification (b) of S9.6 in KYSE30 cells treated with 5μg/ml DDP for 2h (n>50). Scale bars, 10 μm. (c) DHX9 Protein expression in KYSE30 cells treated with different concentration of DDP for 2h. (d-e) Fluorescence image (d) and quantification (e) of DHX9 in KYSE30 cells treated with 5μg/ml DDP for 2h (n>50). Scale bars, 10 μm. (f) RAD51 Protein expression in KYSE30 cells treated with different concentration of DDP for 2h. (g-h) Fluorescence image (g) and quantification (h) of RAD51 in KYSE30 cells treated with 5μg/ml DDP for 2h (n>50). Scale bars, 10 μm. Statistical significance is indicated as ****P < 0.0001, ***P < 0.001, **P < 0.01, *P < 0.05.

**
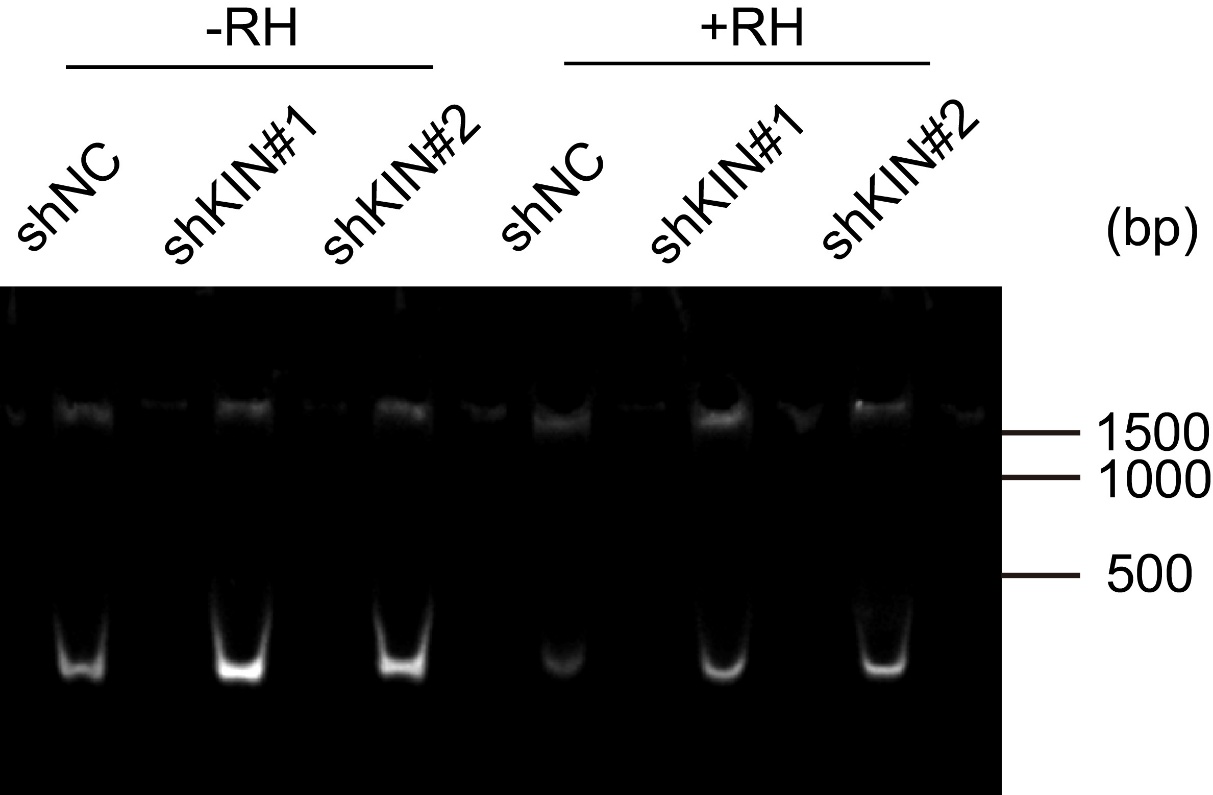
**

Figure. S6. Fluorescence image of R-loop from DRIP. RNA-DNA hybrids extracted from shNC and shKIN KYSE30 cells and purified using S9.6 coated beads, with mock and RNase H treatment before pull-down.


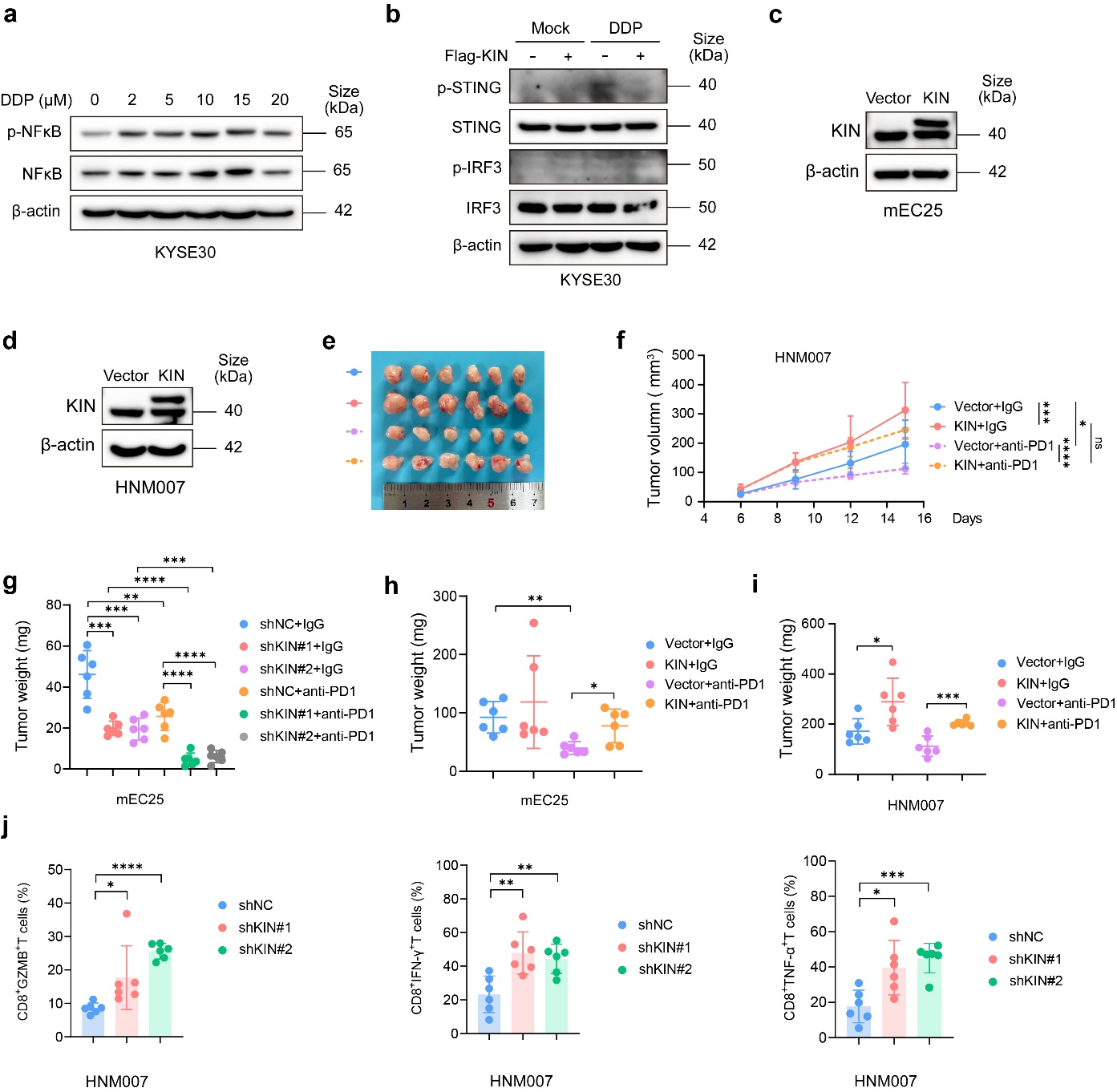


Figure. S7. NFκB is activated under treatment of cisplatin. (a) STING activation *via* NFκB detected by WB in KYSE30 treated with different concentration of DDP for 2h. (b) STING activation *via* IRF3 detected by WB in KYSE30 expressed KIN or empty vector treated with 5μg/ml DDP for 2h. (c) KIN protein expression in mEC25 cells expressing KIN or empty vector. (d) KIN protein expression in HNM007 cells expressing KIN or empty vector. (e-f) Representative image and tumor growth curves of HNM007 tumors expressing KIN or empty vector treated with anti-PD-1 antibody or isotype (n=6). (g) Tumor weight of shNC and shKIN mEC25 tumors treated with anti-PD-1 antibody (n=6). (h) Tumor weight of mEC25 tumors expressing KIN or empty vector treated with anti-PD-1 antibody (n=6). (i) Tumor weight of HNM007 tumors expressing KIN or empty vector treated with anti-PD-1 antibody (n=6). (j) Percentages of infiltrating CD8^+^GZMB^+^T cells, CD8^+^IFN-γ^+^T cells and CD8^+^TNF-α^+^T cells in shNC and shKIN HNM007 tumors (n=6) were analyzed by flow cytometry. Statistical significance is indicated as ****P < 0.0001, ***P < 0.001, **P < 0.01, *P < 0.05.
