# Supplementary material for: DNA/RNA-binding protein KIN17 supports esophageal cancer progression via resolving noncanonical STING activation induced by R-loop
Source: Signal Transduct Target Ther. 2025 Aug 15;10:256. doi: 10.1038/s41392-025-02344-2 (PMC12354822; doi:10.1038/s41392-025-02344-2)
Supplement: Supplementary file 2 — The raw data of Western blot [file 41392_2025_2344_MOESM2_ESM.pptx]

## Slide 1
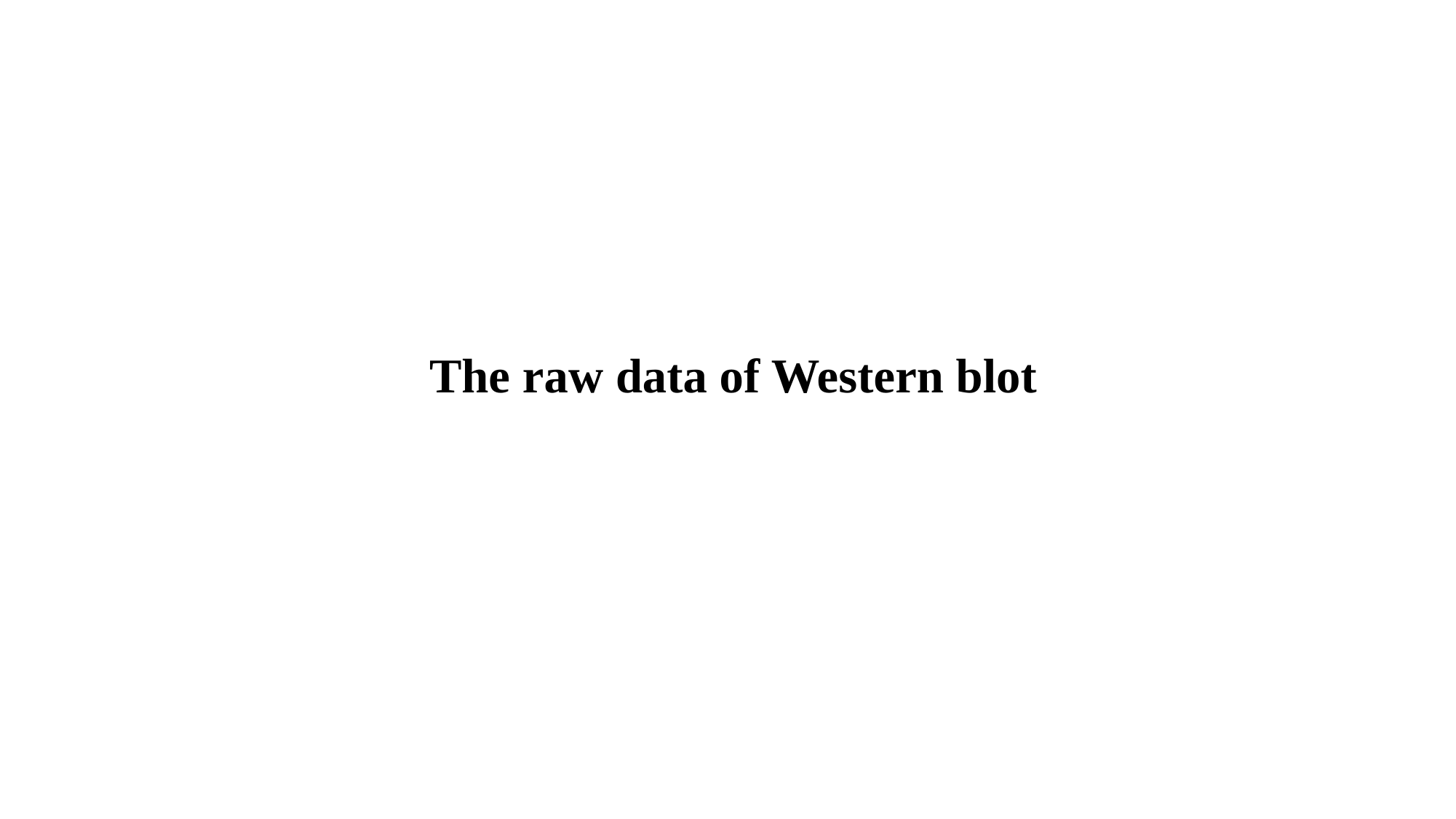

The raw data of Western blot

## Slide 2
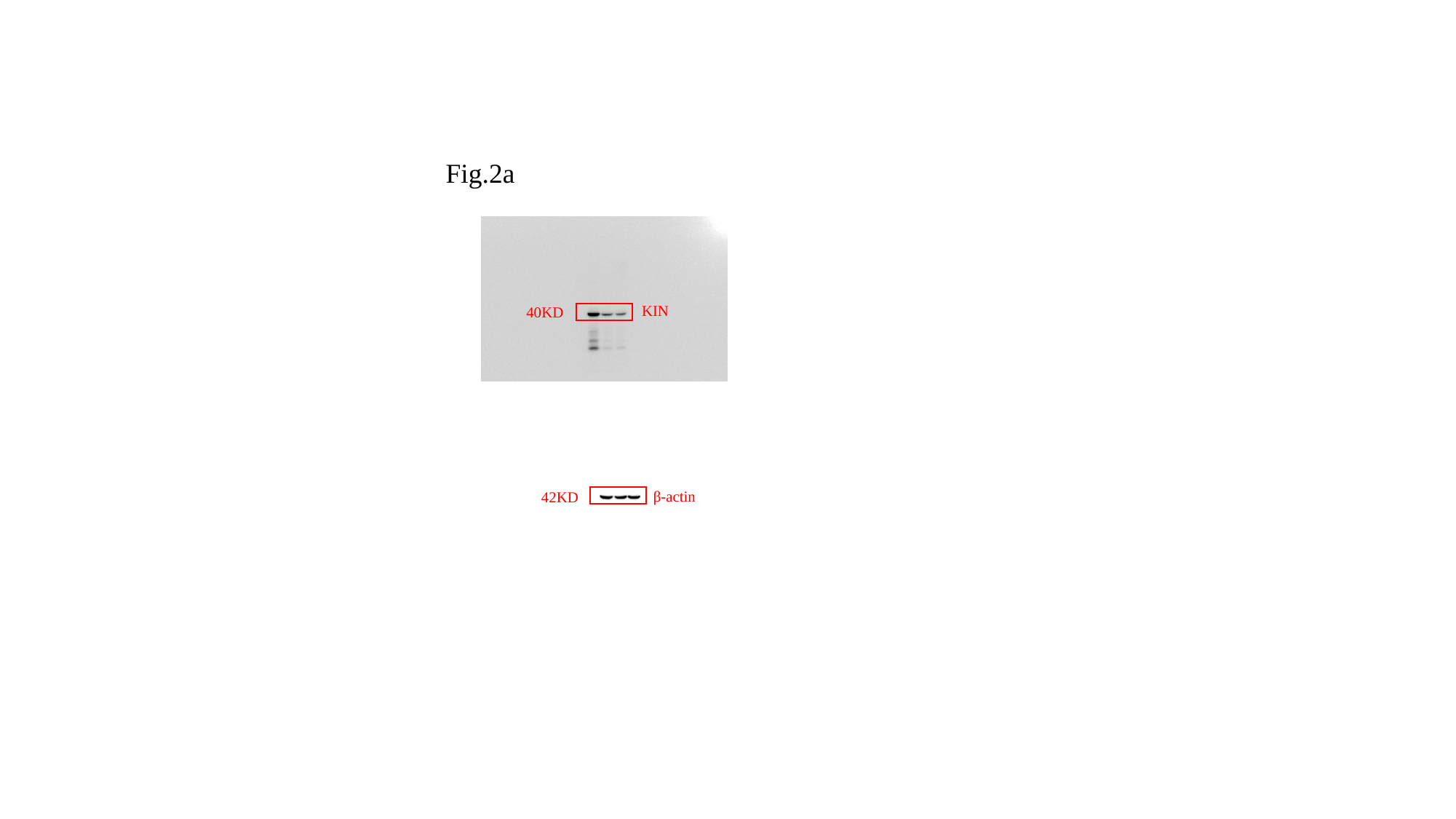

Fig.2a
KIN
40KD
β-actin
42KD

## Slide 3
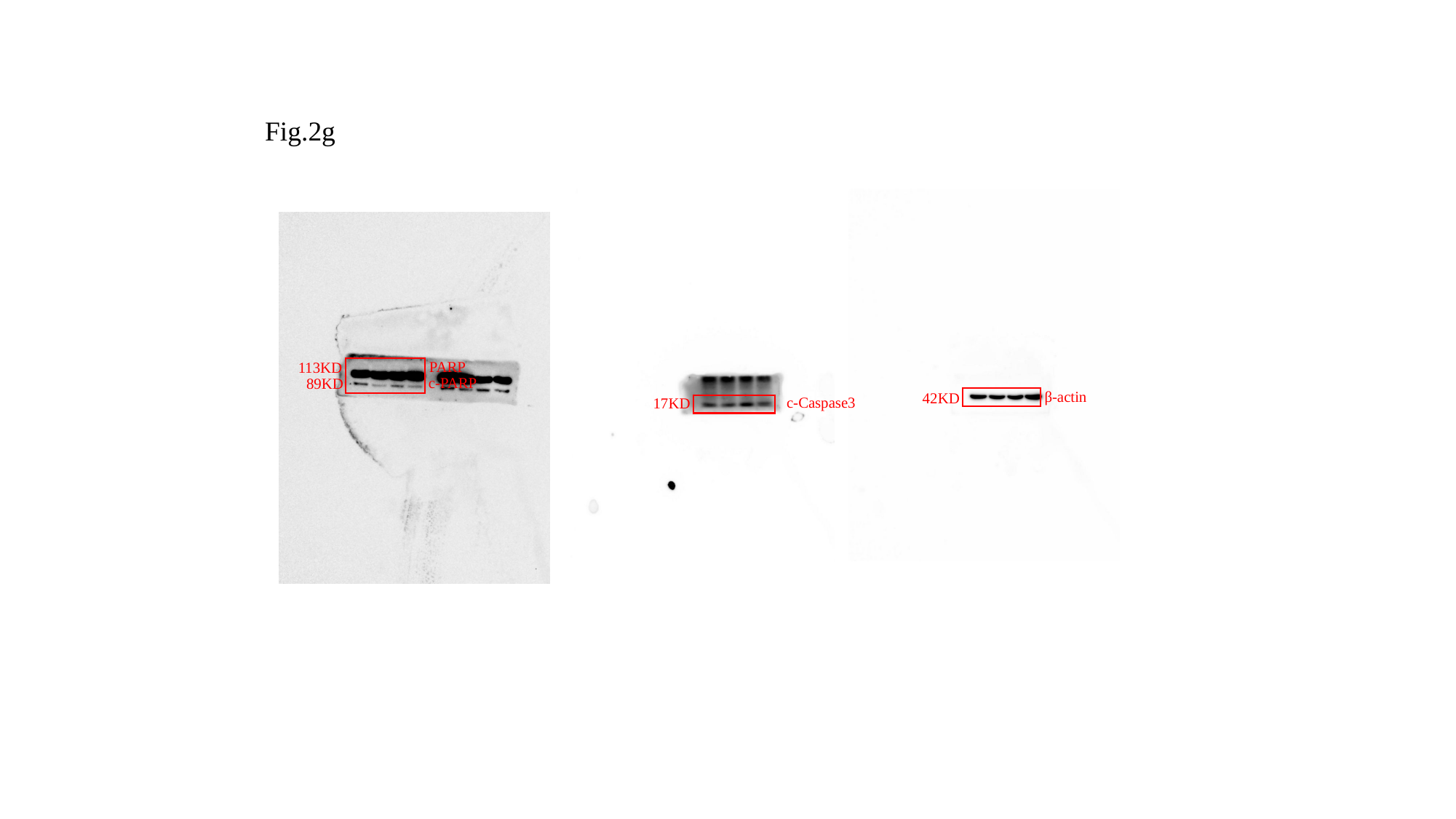

Fig.2g
PARP
113KD
c-PARP
89KD
β-actin
42KD
c-Caspase3
17KD

## Slide 4
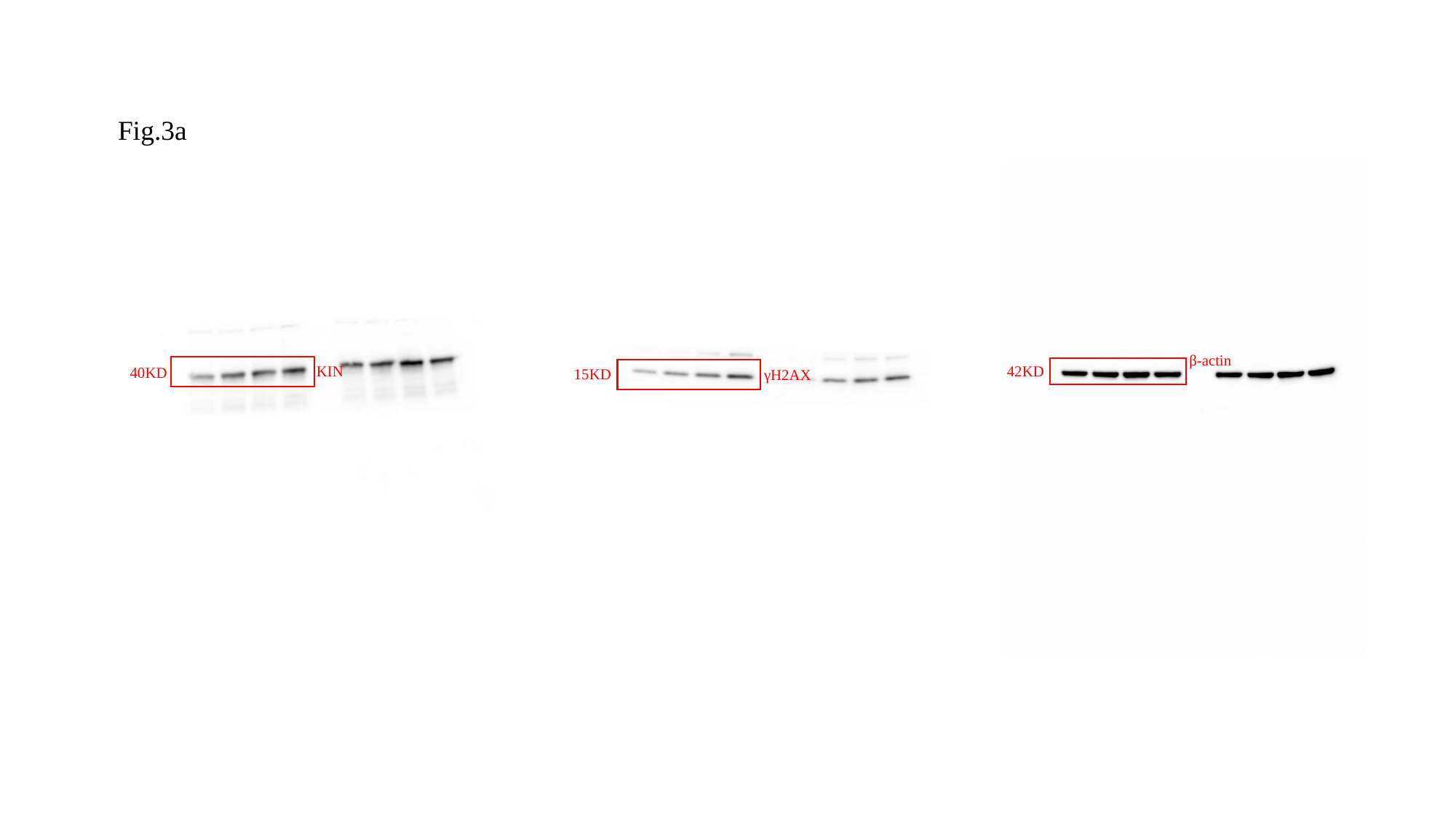

Fig.3a
β-actin
KIN
42KD
40KD
15KD
γH2AX

## Slide 5
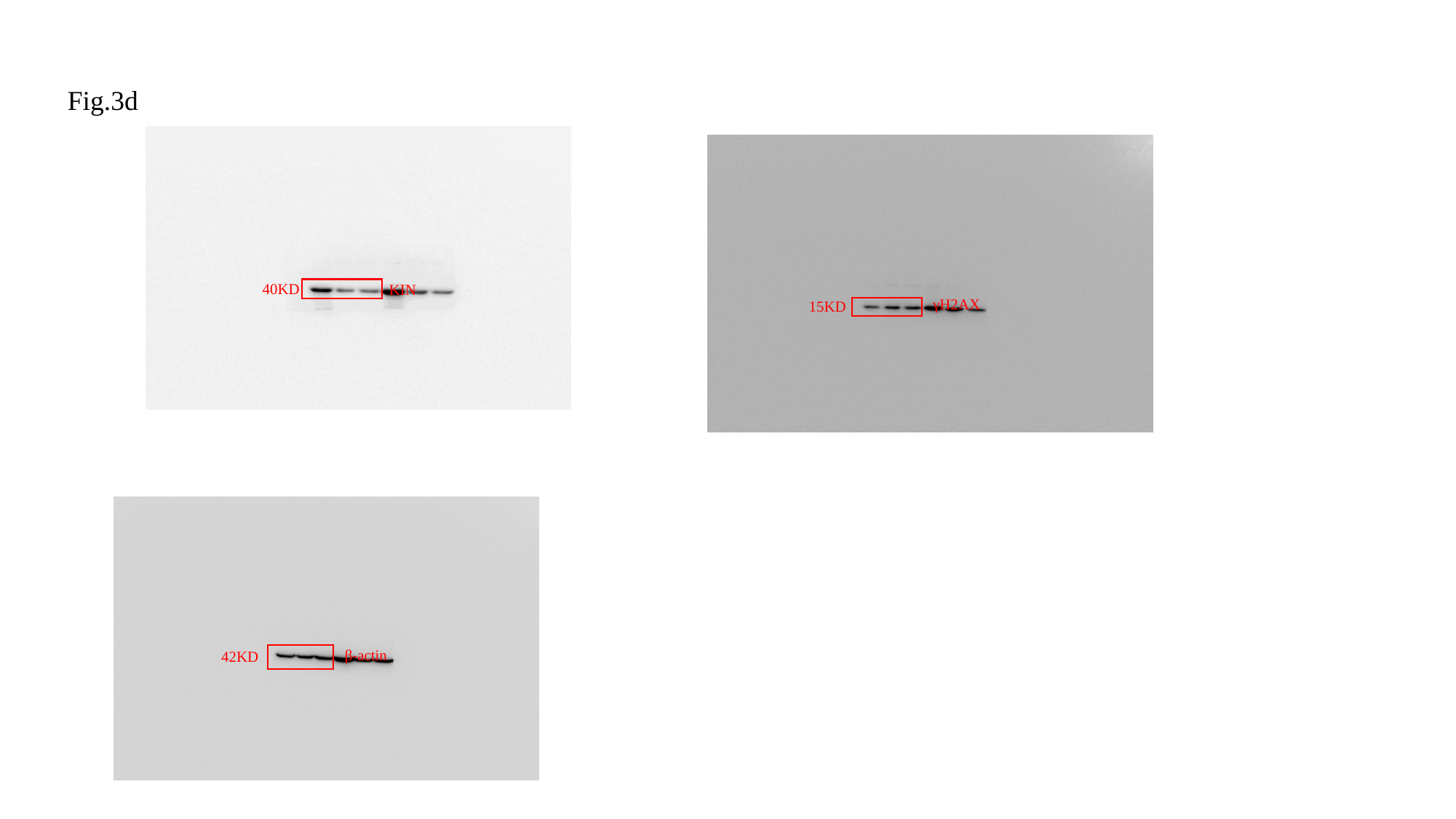

Fig.3d
40KD
KIN
γH2AX
15KD
β-actin
42KD

## Slide 6
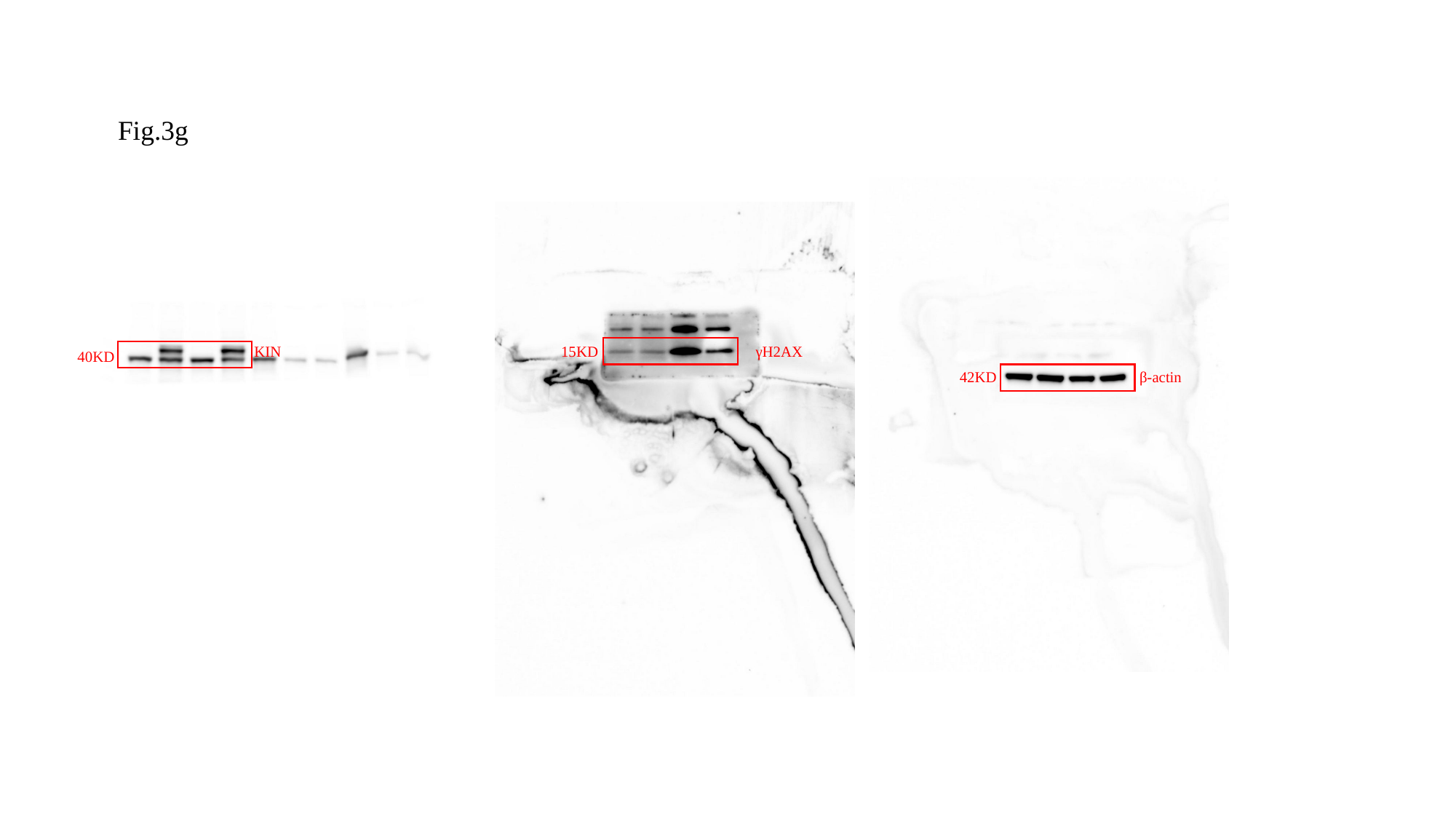

Fig.3g
15KD
KIN
γH2AX
40KD
42KD
β-actin

## Slide 7
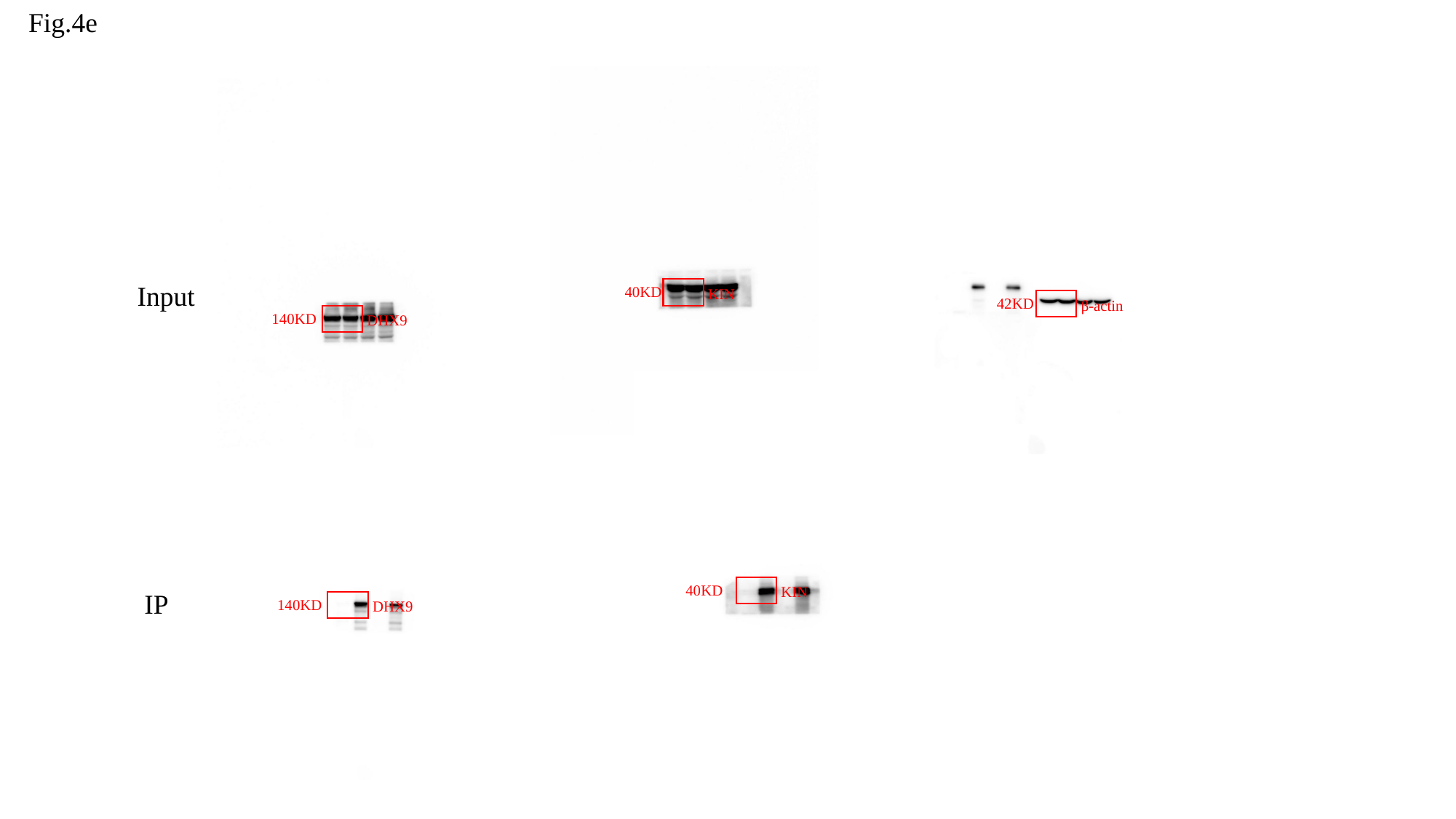

Fig.4e
Input
40KD
KIN
42KD
β-actin
140KD
DHX9
40KD
KIN
IP
140KD
DHX9

## Slide 8
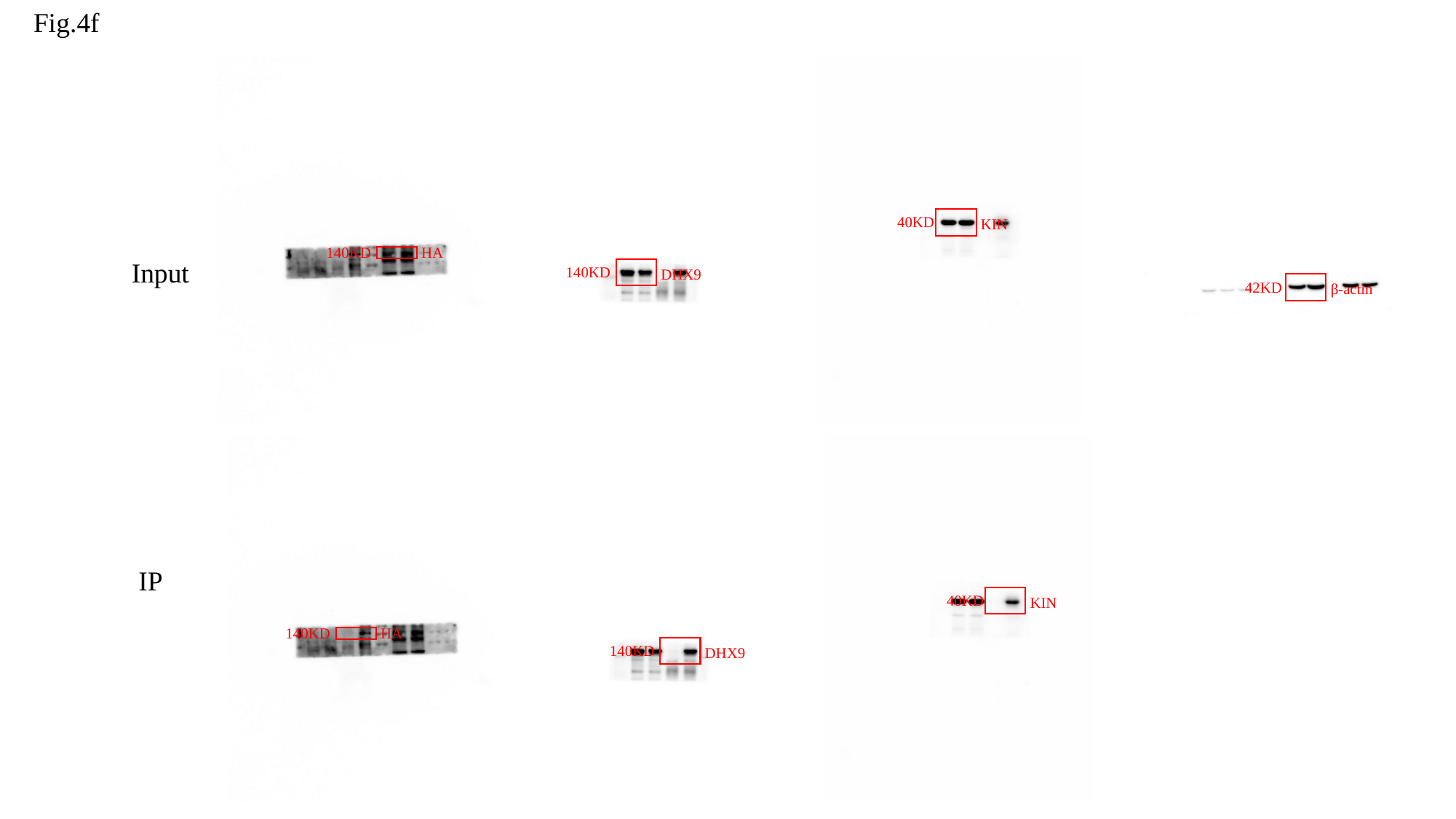

Fig.4f
40KD
KIN
140KD
HA
Input
140KD
DHX9
42KD
β-actin
IP
40KD
KIN
140KD
HA
140KD
DHX9

## Slide 9
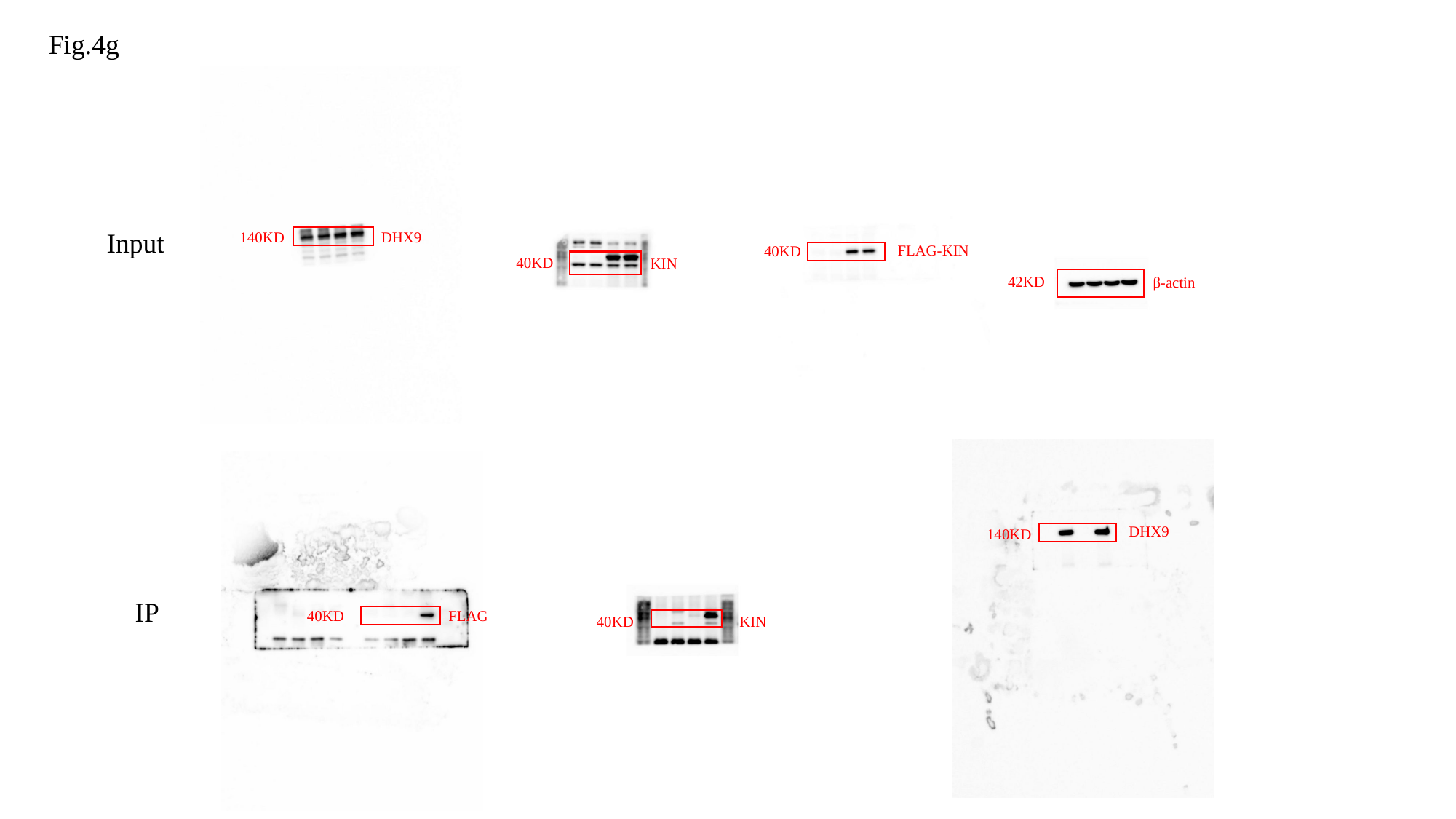

Fig.4g
Input
140KD
DHX9
FLAG-KIN
40KD
40KD
KIN
42KD
β-actin
DHX9
140KD
IP
40KD
FLAG
KIN
40KD

## Slide 10
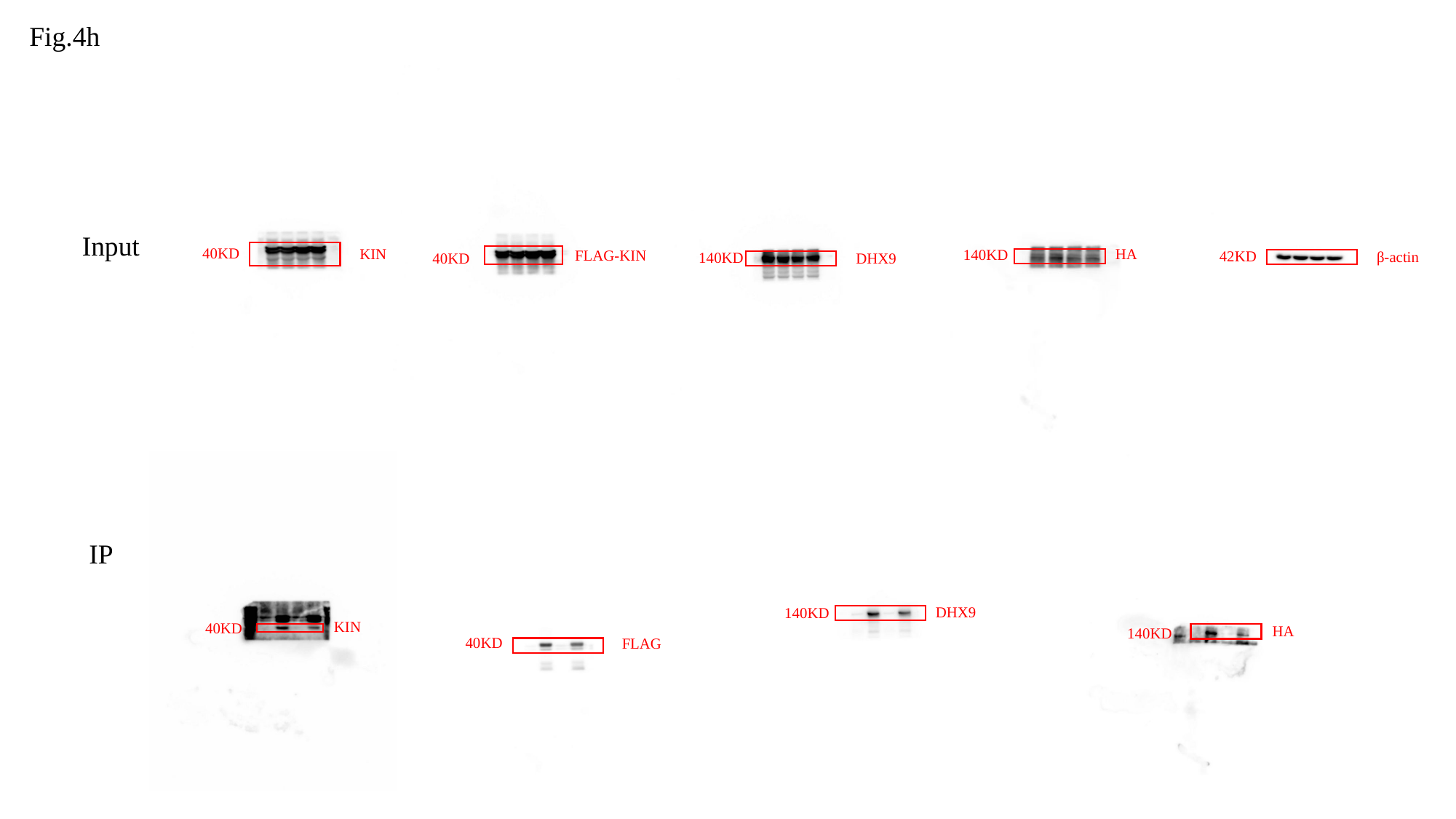

Fig.4h
Input
40KD
KIN
HA
140KD
FLAG-KIN
42KD
β-actin
140KD
DHX9
40KD
IP
DHX9
140KD
KIN
40KD
HA
140KD
40KD
FLAG

## Slide 11
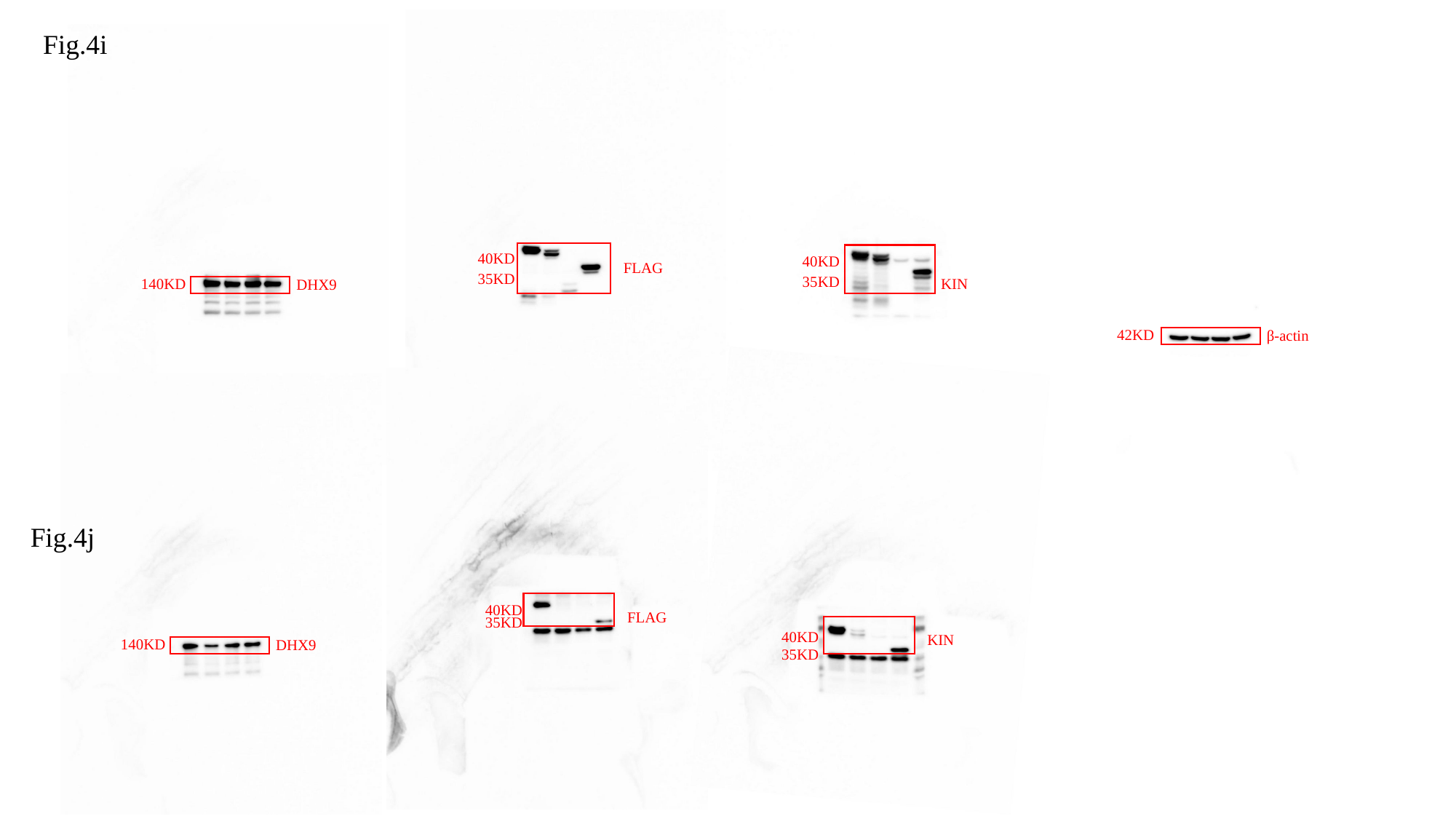

Fig.4i
40KD
40KD
FLAG
35KD
35KD
140KD
KIN
DHX9
42KD
β-actin
Fig.4j
40KD
FLAG
35KD
40KD
KIN
140KD
DHX9
35KD

## Slide 12
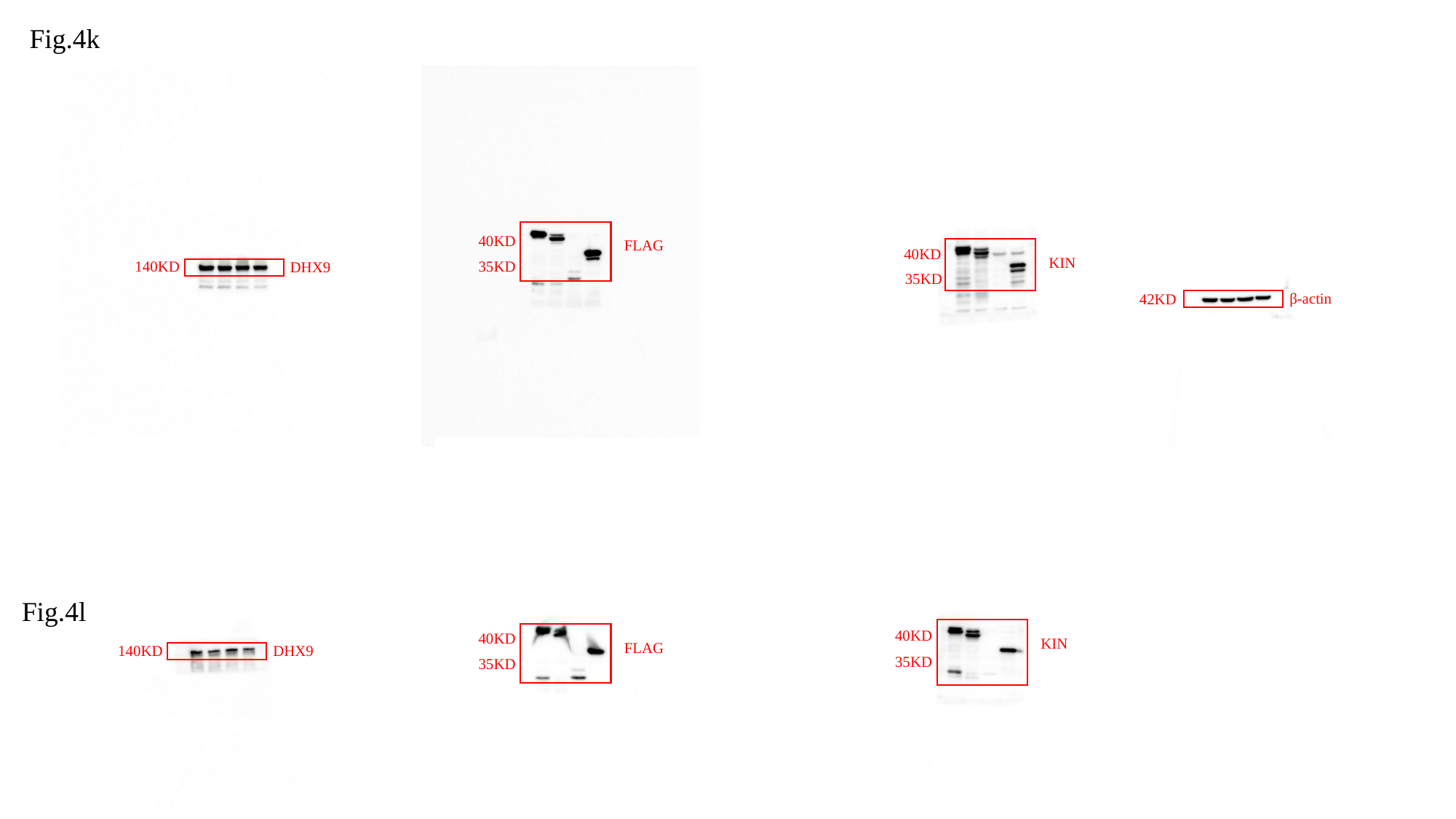

Fig.4k
40KD
FLAG
40KD
KIN
140KD
35KD
DHX9
35KD
β-actin
42KD
Fig.4l
40KD
40KD
KIN
FLAG
140KD
DHX9
35KD
35KD

## Slide 13
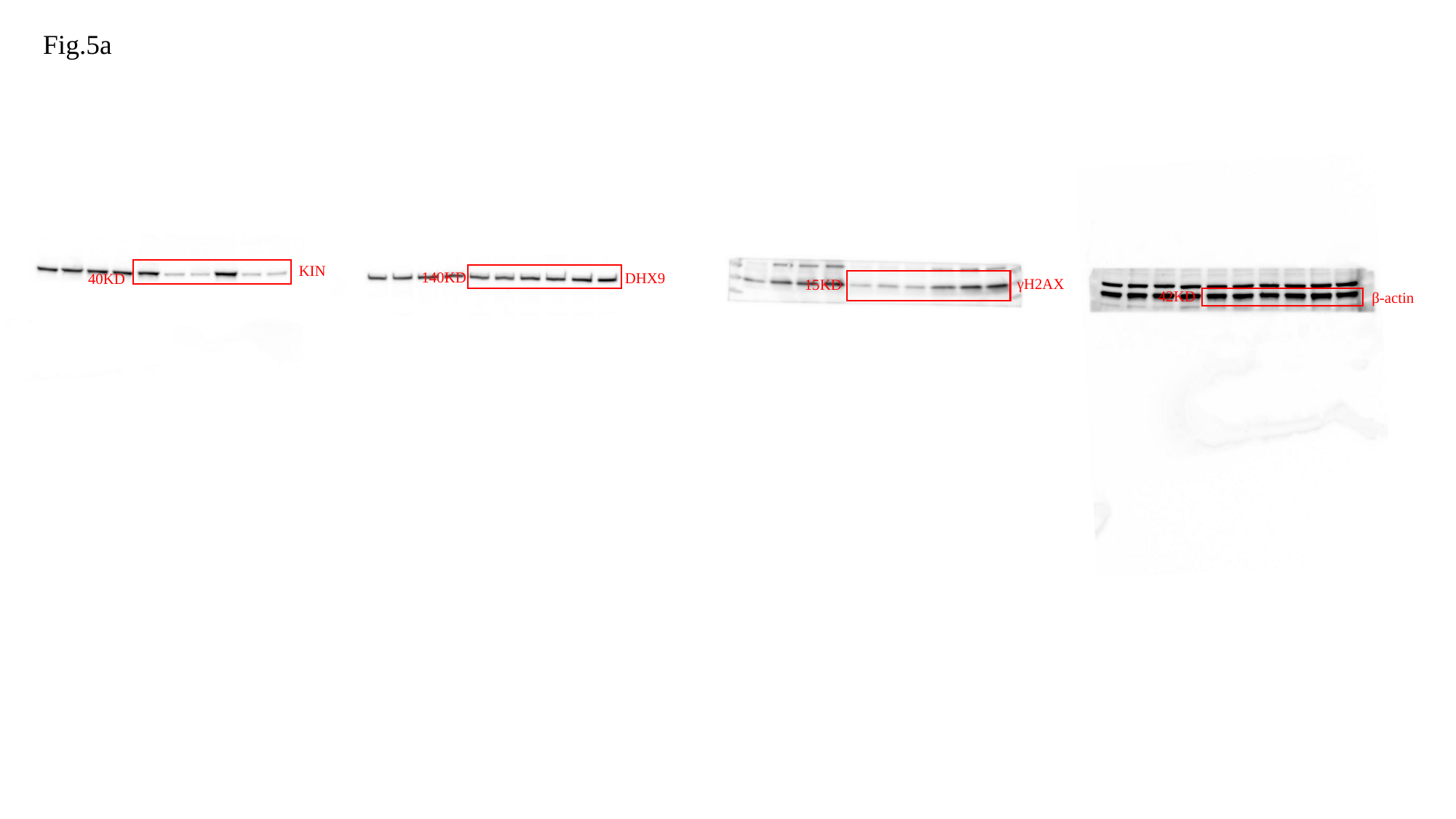

Fig.5a
KIN
140KD
DHX9
40KD
γH2AX
15KD
42KD
β-actin

## Slide 14
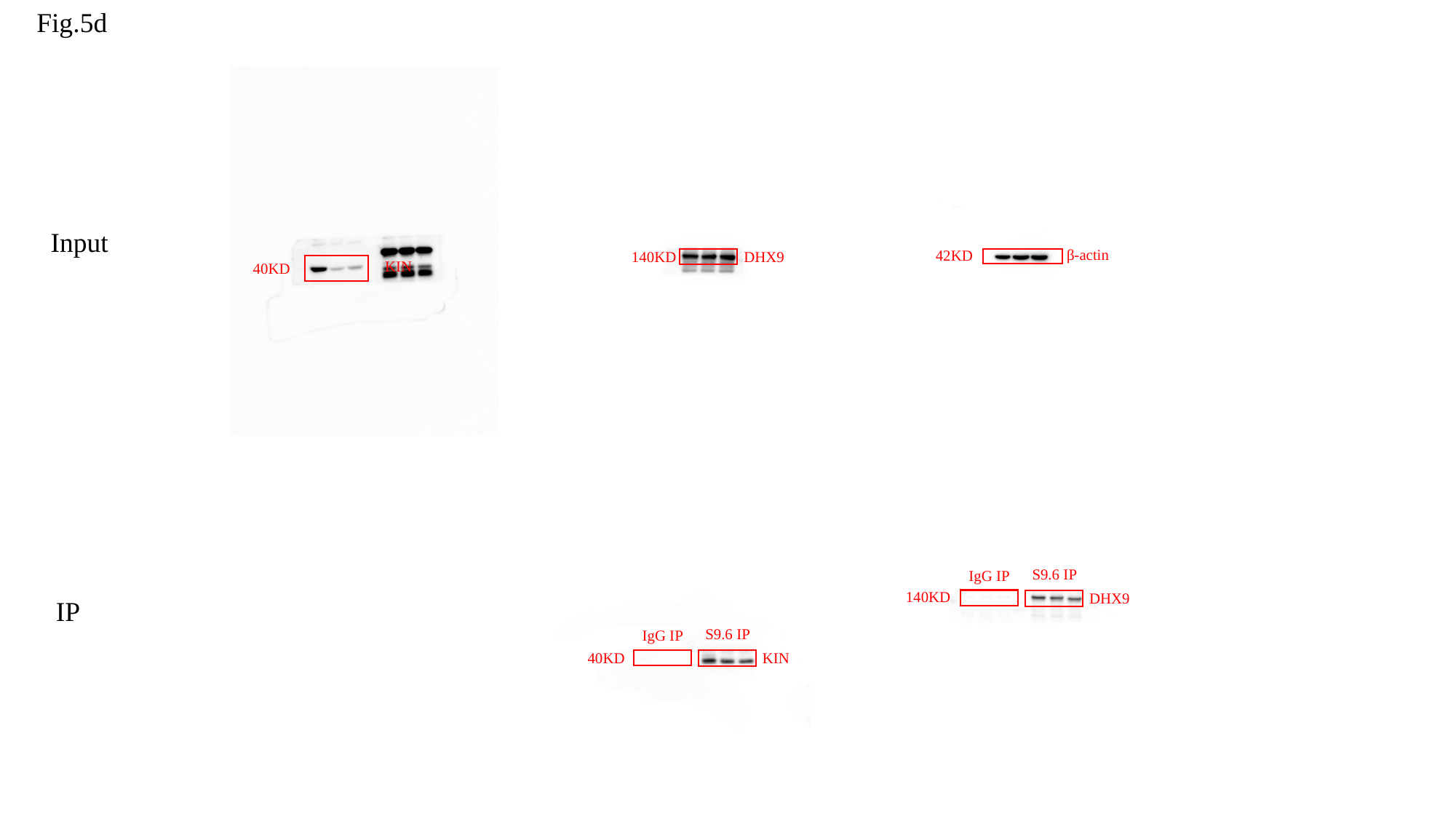

Fig.5d
Input
β-actin
42KD
140KD
DHX9
KIN
40KD
S9.6 IP
IgG IP
140KD
DHX9
IP
S9.6 IP
IgG IP
40KD
KIN

## Slide 15
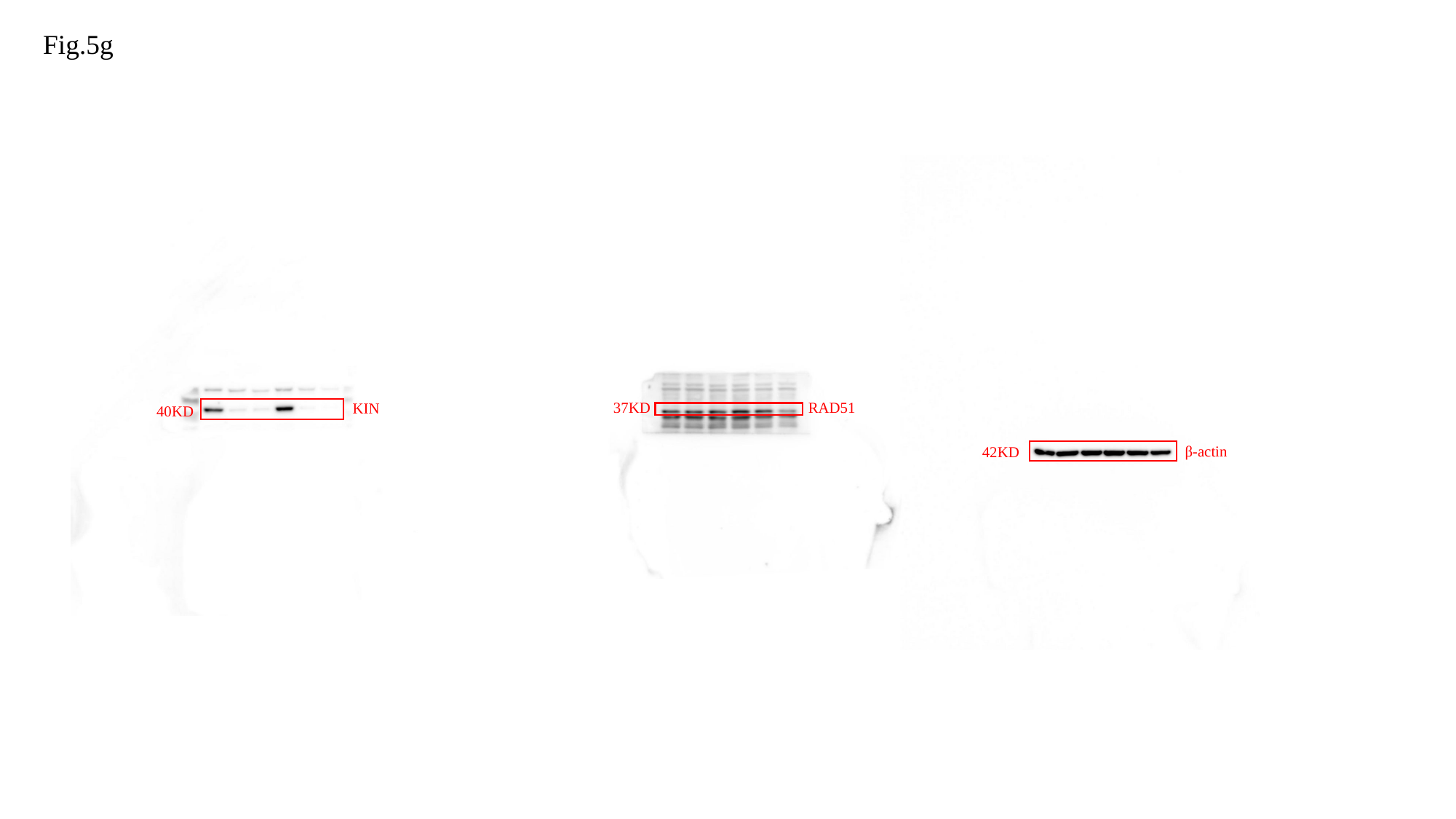

Fig.5g
RAD51
37KD
KIN
40KD
β-actin
42KD

## Slide 16
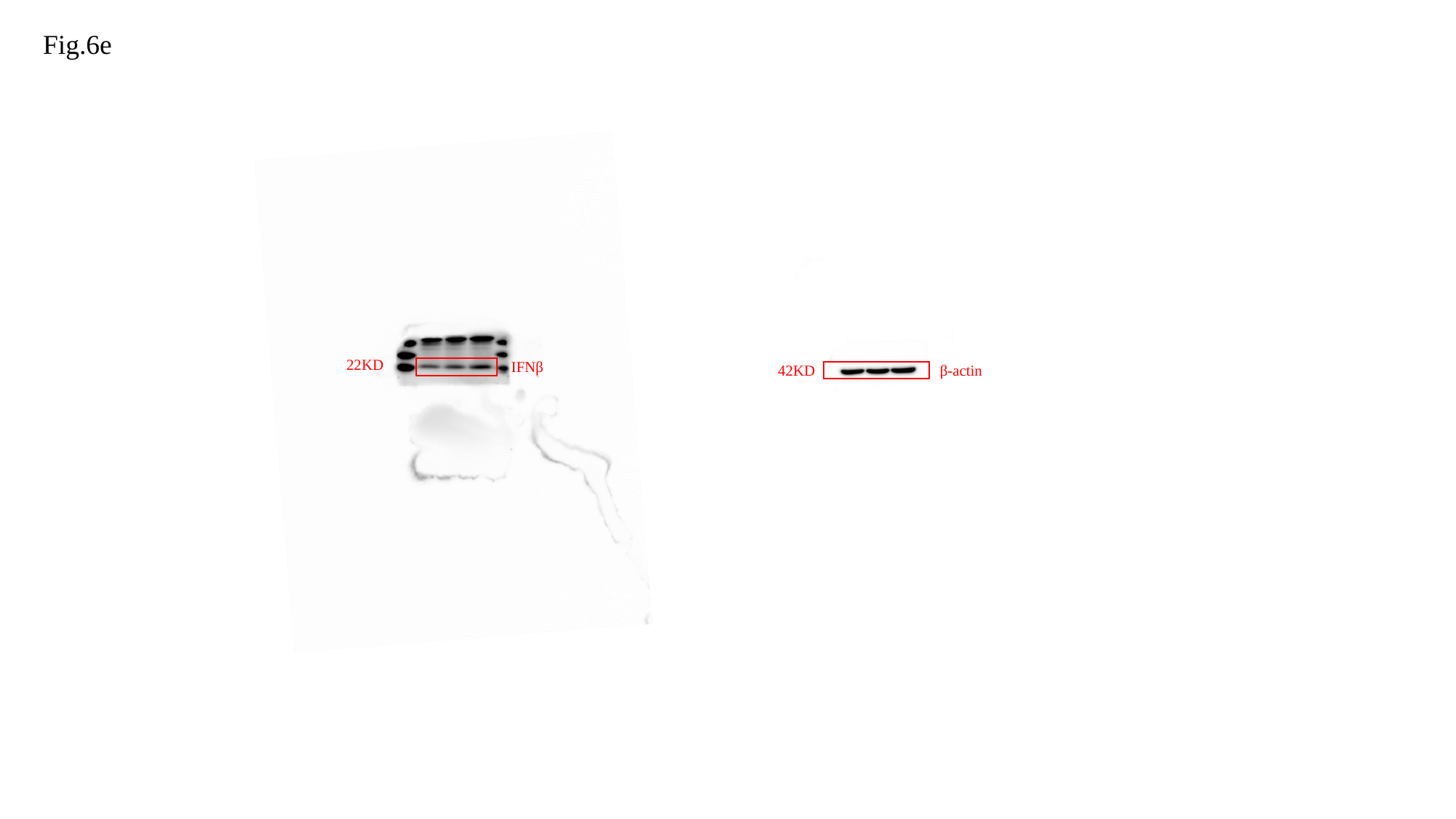

Fig.6e
22KD
IFNβ
β-actin
42KD

## Slide 17
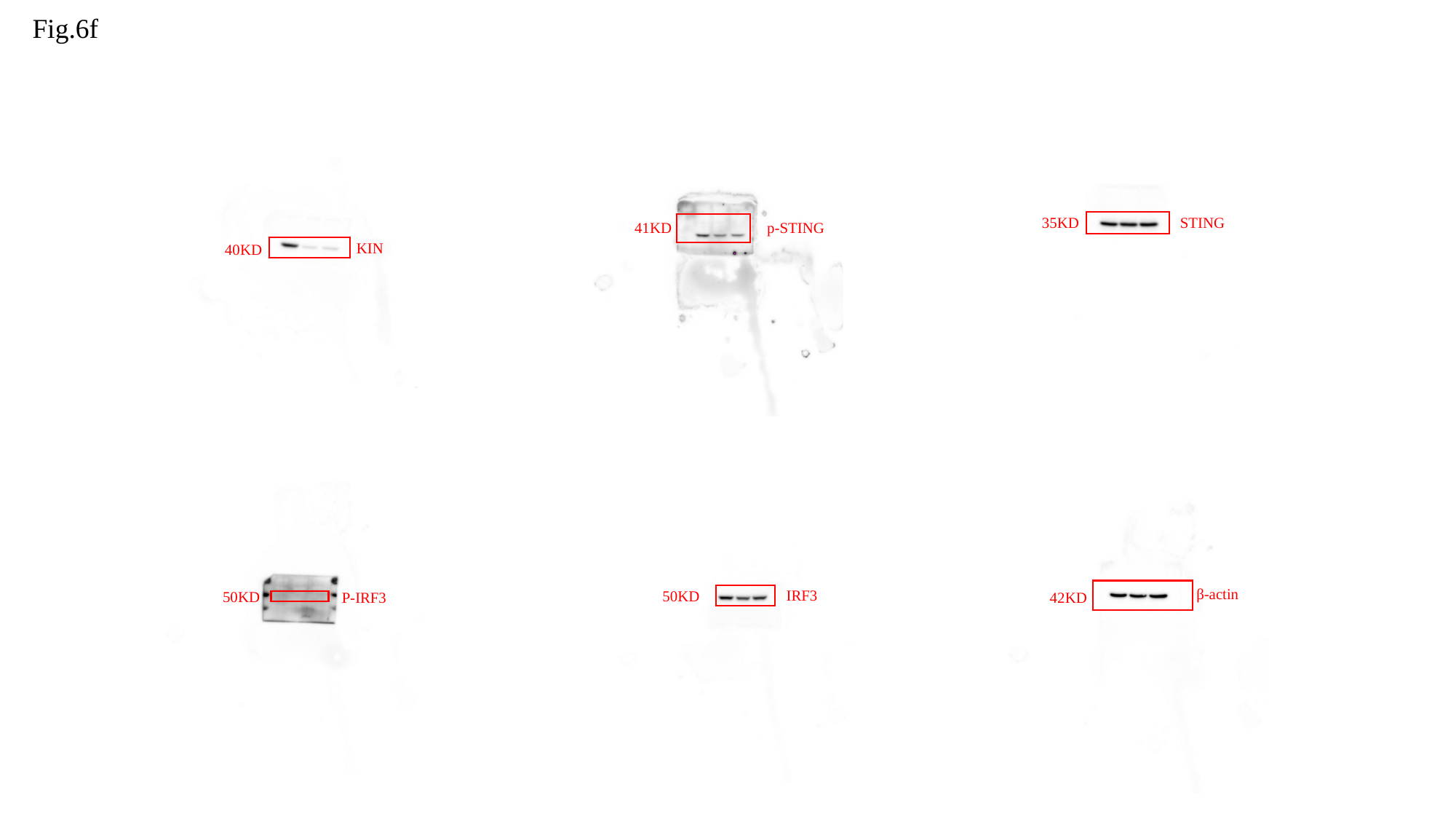

Fig.6f
STING
35KD
41KD
p-STING
KIN
40KD
β-actin
IRF3
50KD
50KD
P-IRF3
42KD

## Slide 18
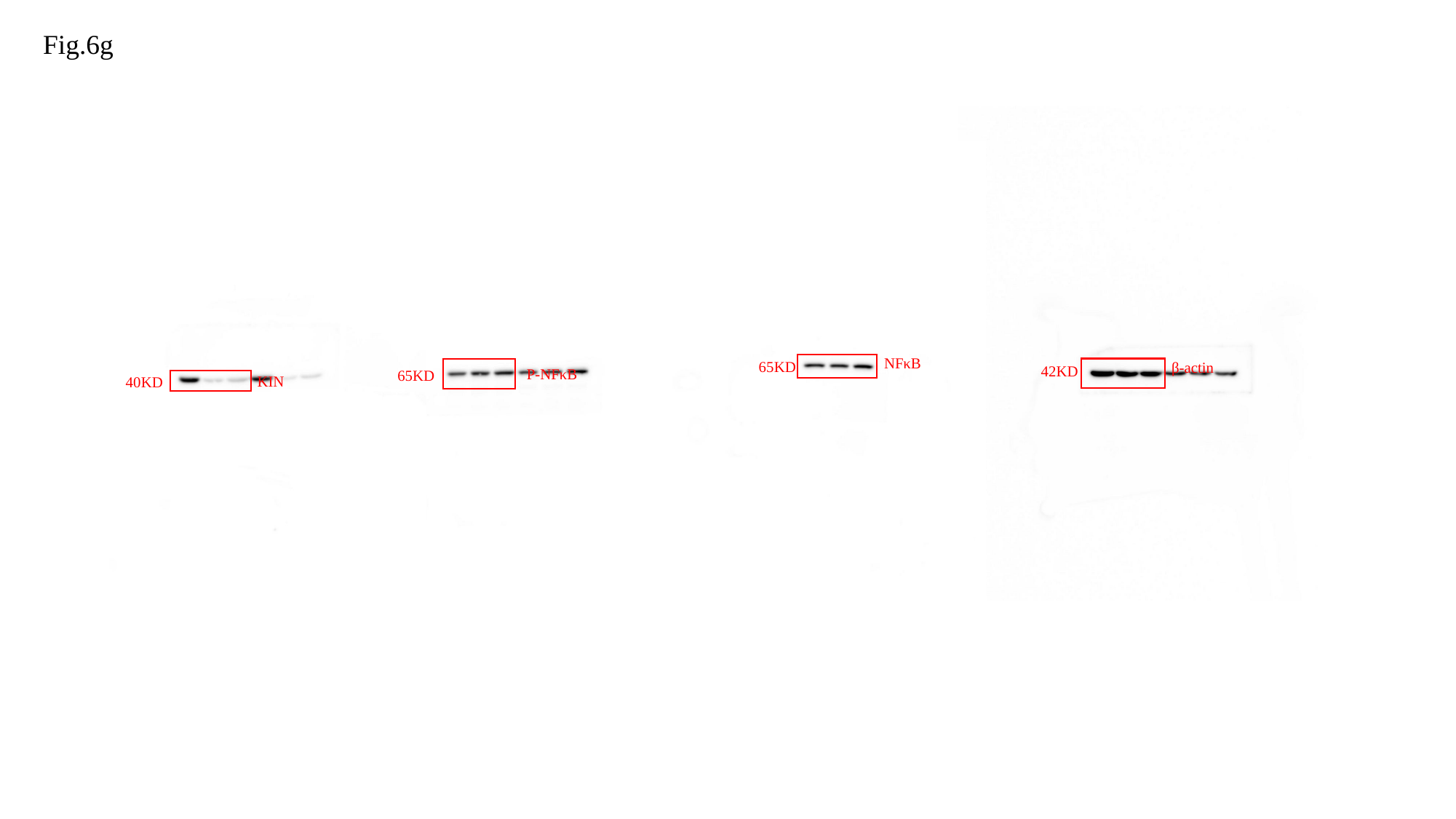

Fig.6g
NFκB
65KD
β-actin
42KD
P-NFκB
65KD
KIN
40KD

## Slide 19
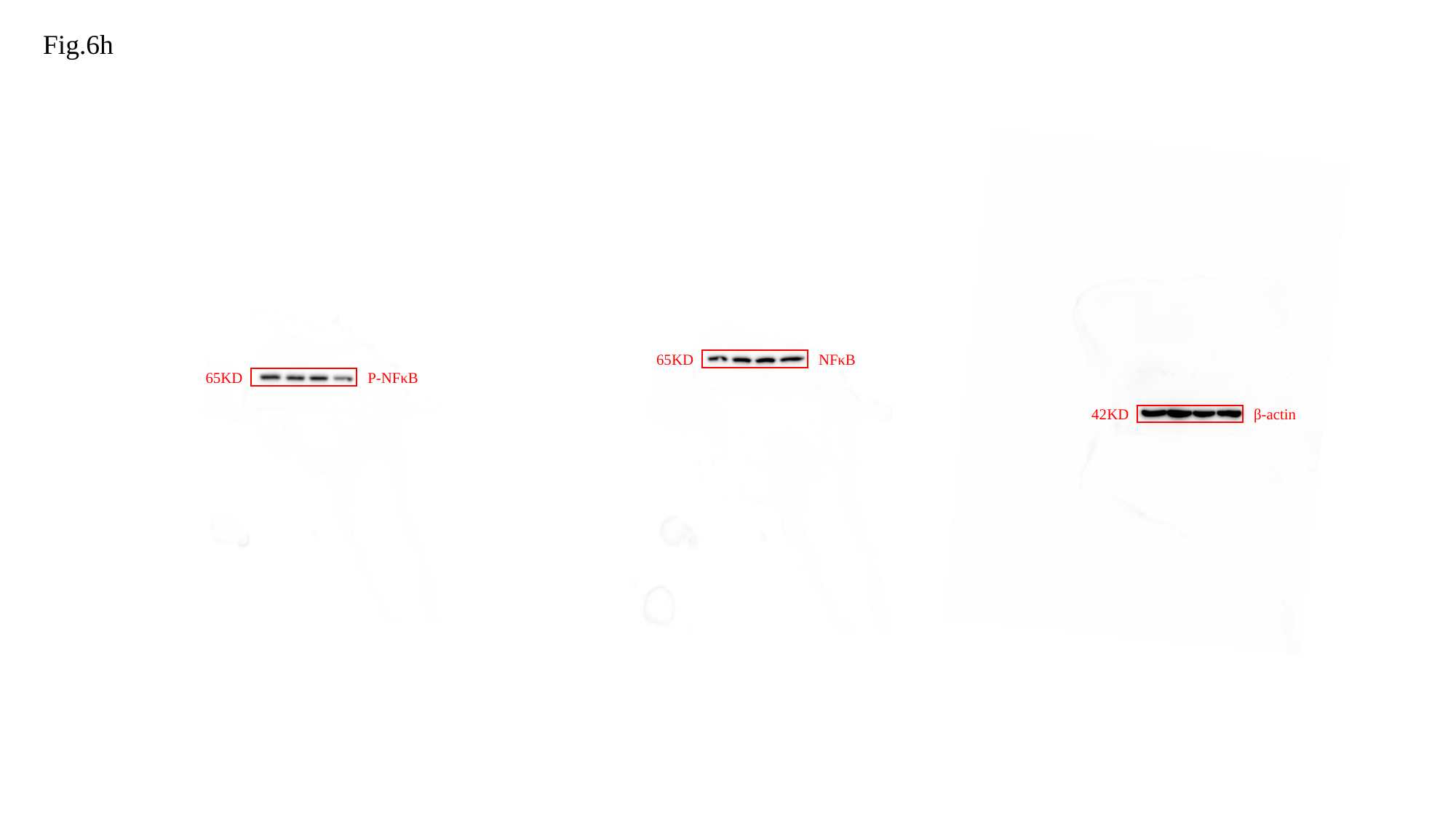

Fig.6h
NFκB
65KD
P-NFκB
65KD
β-actin
42KD

## Slide 20
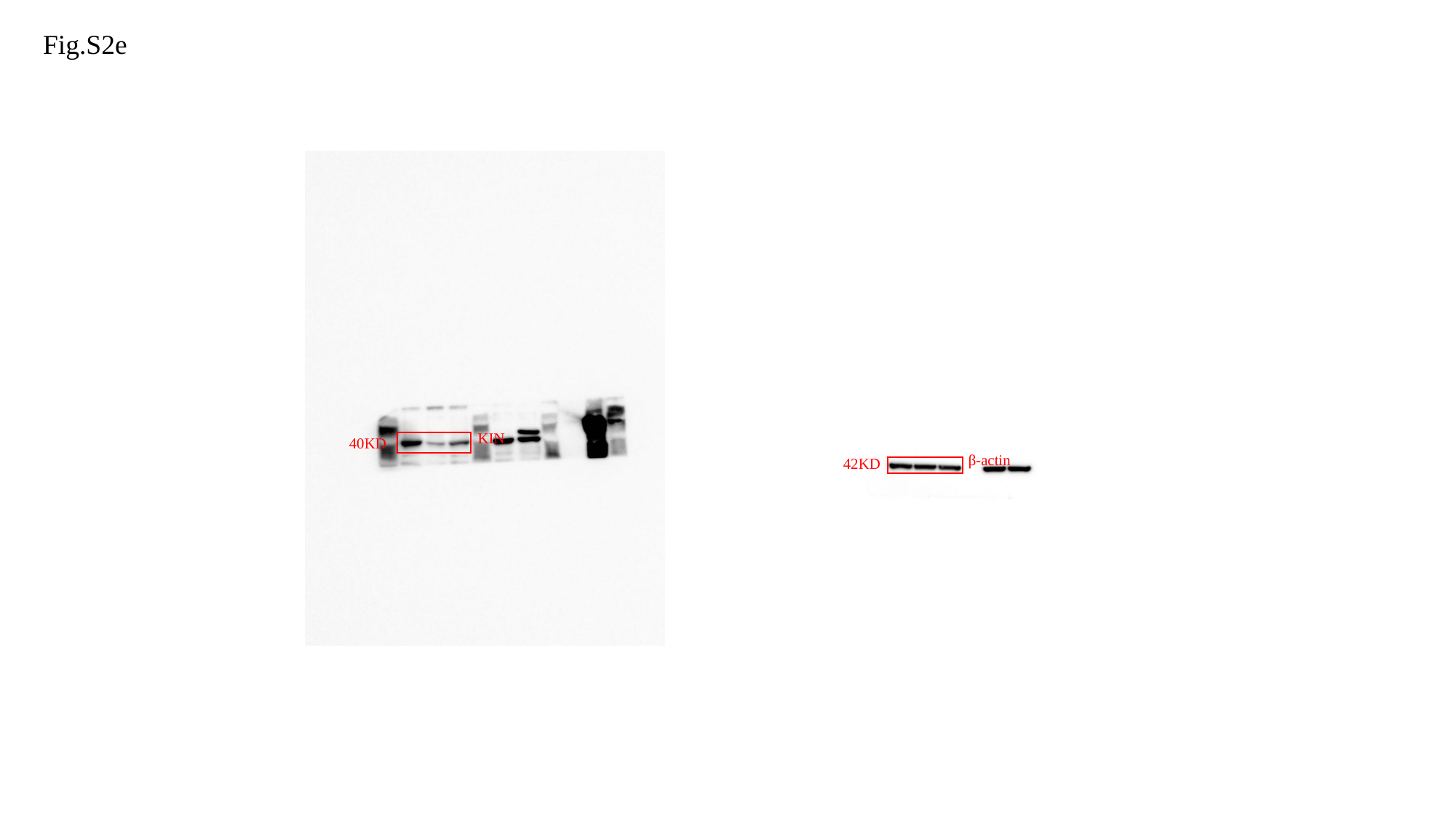

Fig.S2e
KIN
40KD
β-actin
42KD

## Slide 21
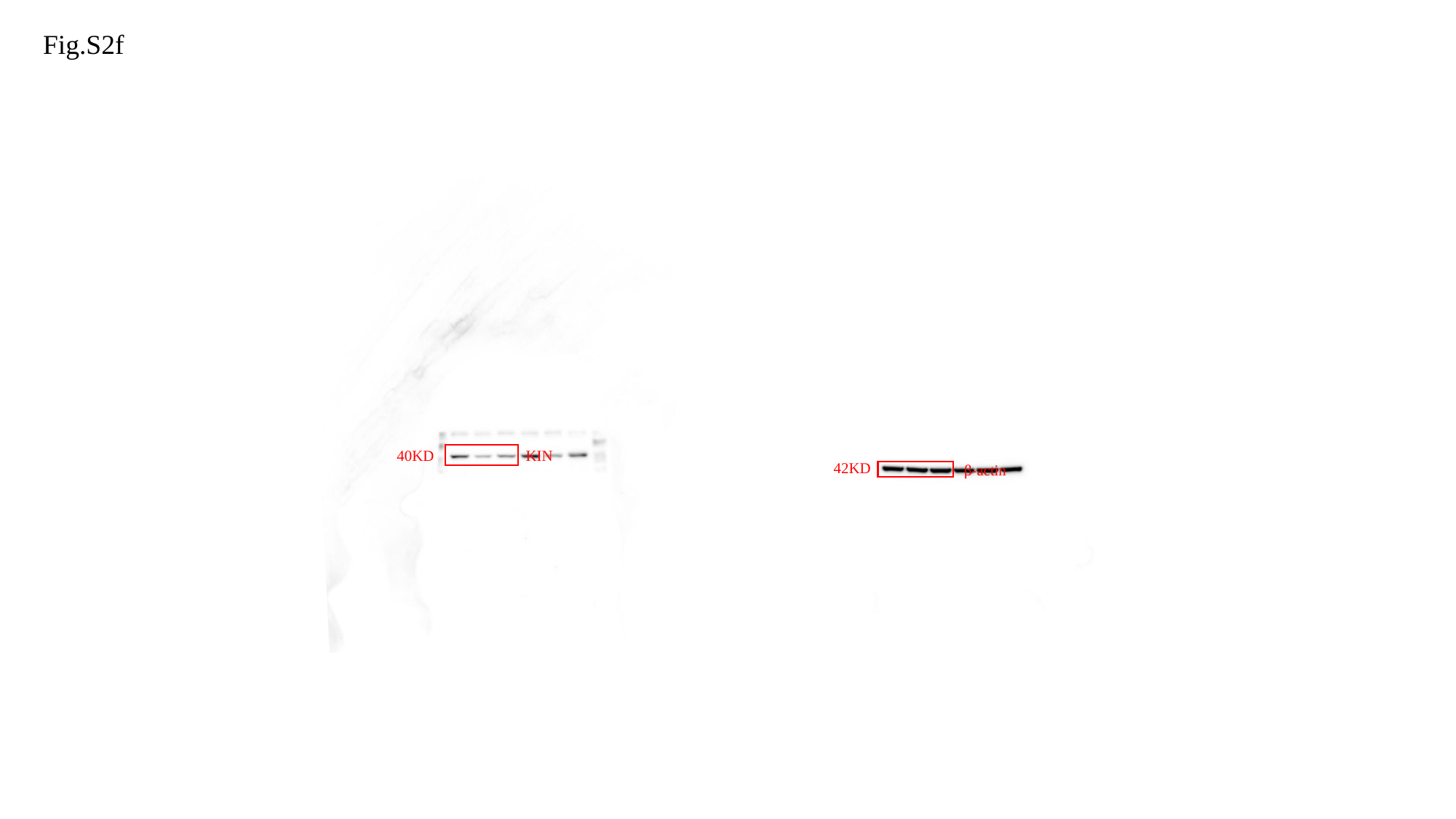

Fig.S2f
40KD
KIN
42KD
β-actin

## Slide 22
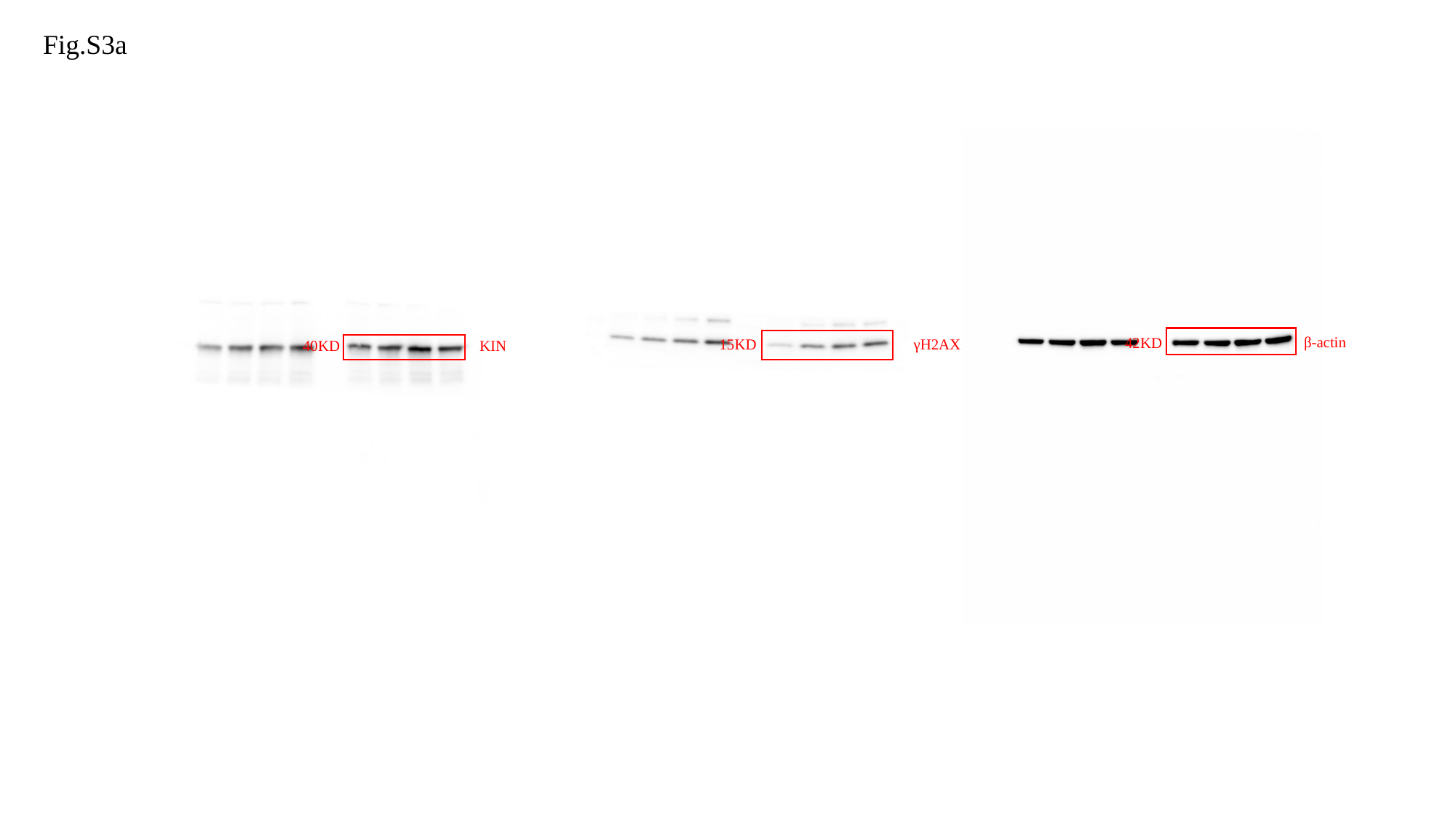

Fig.S3a
β-actin
42KD
15KD
γH2AX
KIN
40KD

## Slide 23
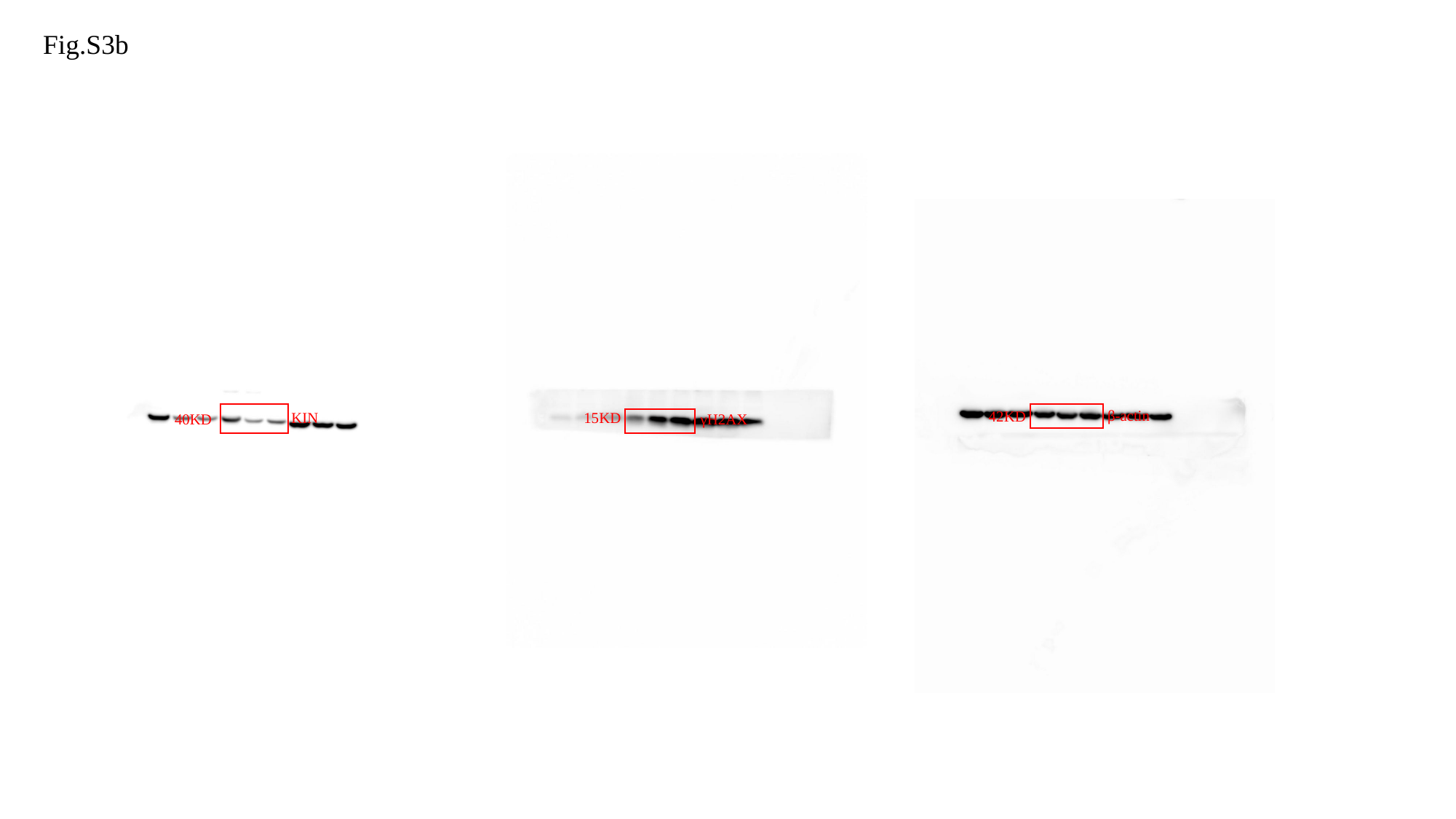

Fig.S3b
β-actin
42KD
KIN
15KD
40KD
γH2AX

## Slide 24
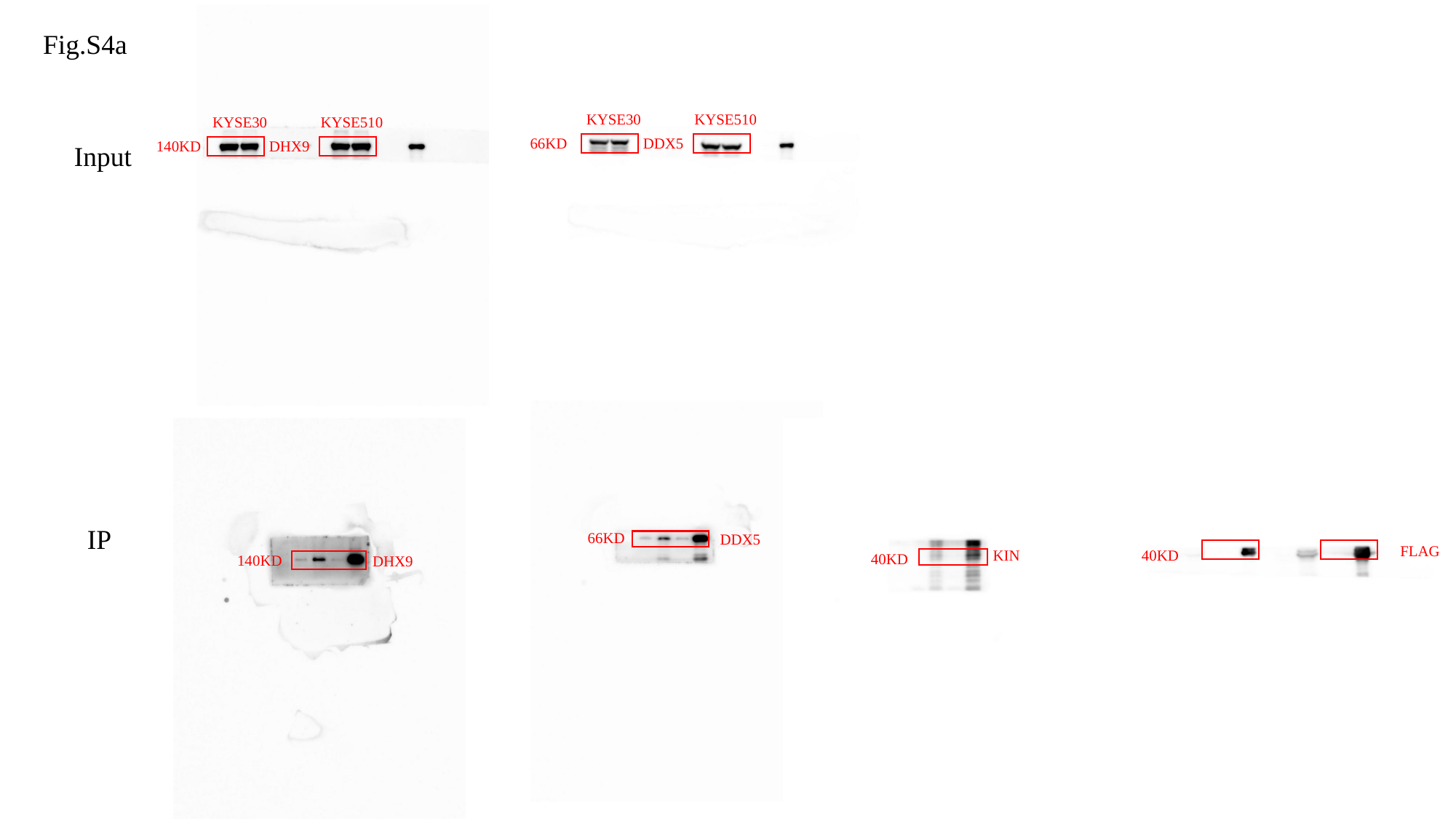

Fig.S4a
KYSE30
KYSE510
KYSE30
KYSE510
DDX5
66KD
DHX9
140KD
Input
IP
66KD
DDX5
FLAG
KIN
40KD
40KD
140KD
DHX9

## Slide 25
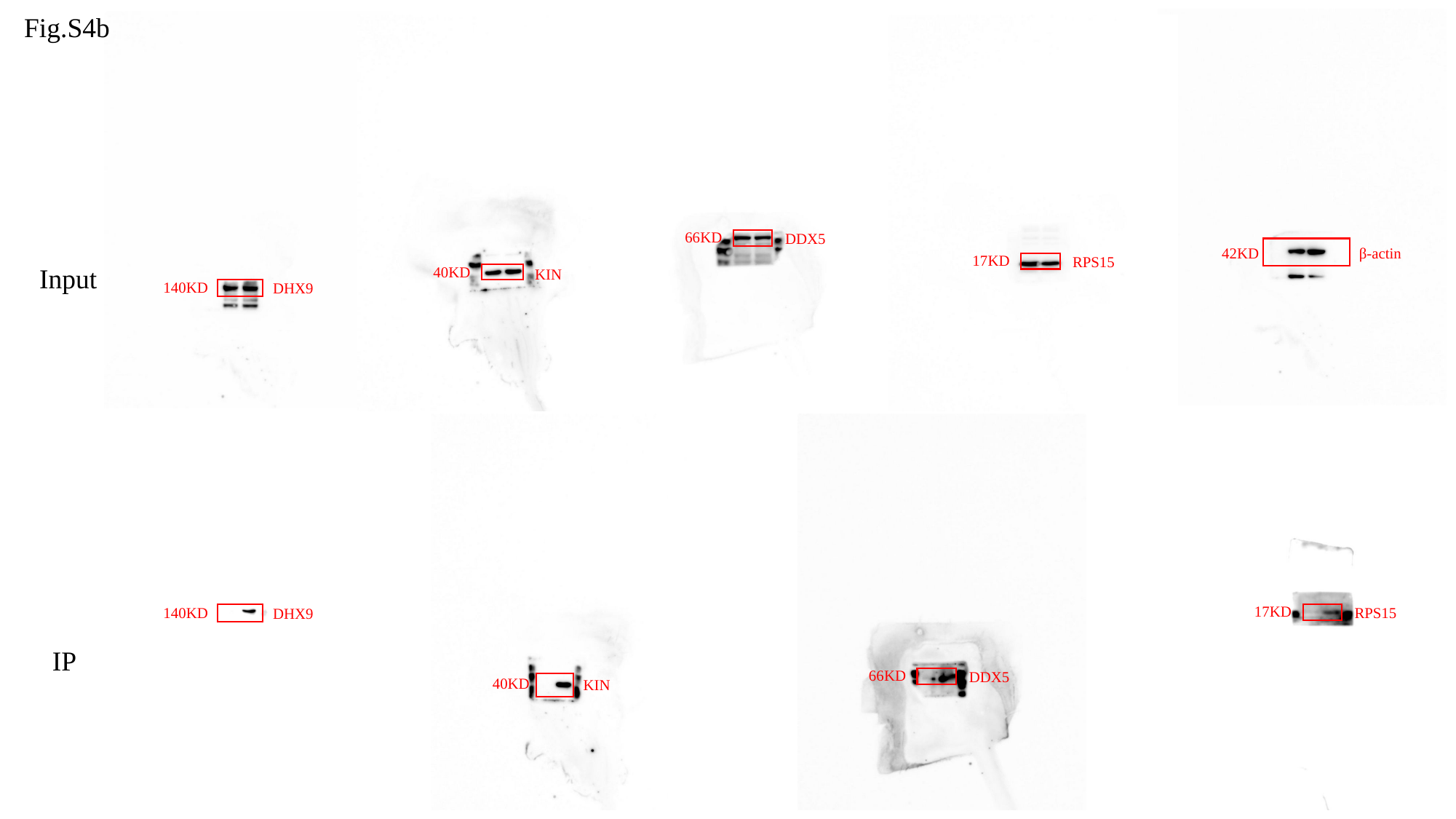

Fig.S4b
66KD
DDX5
42KD
β-actin
17KD
RPS15
Input
40KD
KIN
140KD
DHX9
17KD
140KD
RPS15
DHX9
IP
66KD
DDX5
40KD
KIN

## Slide 26
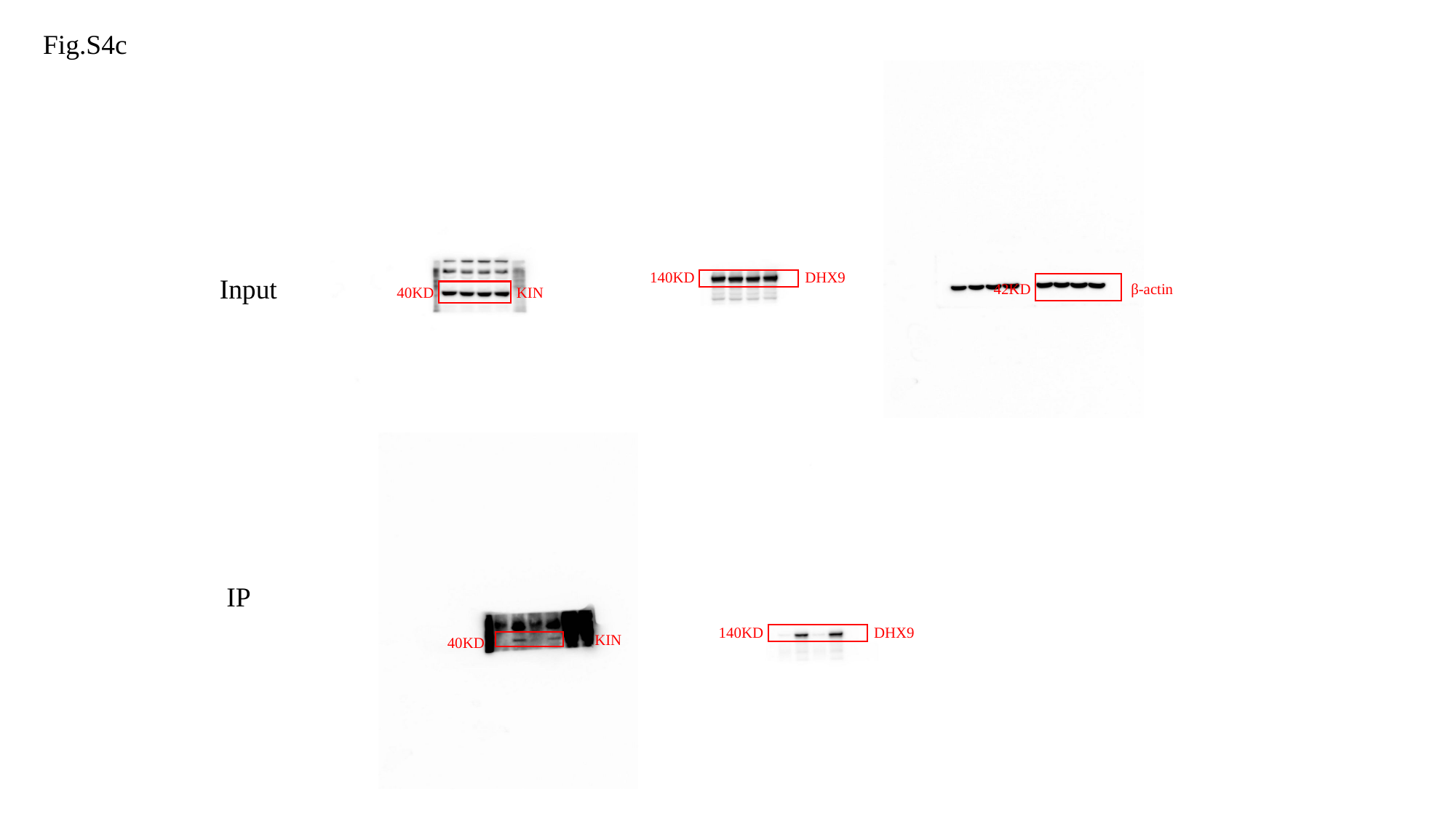

Fig.S4c
140KD
DHX9
Input
42KD
β-actin
40KD
KIN
IP
140KD
DHX9
KIN
40KD

## Slide 27
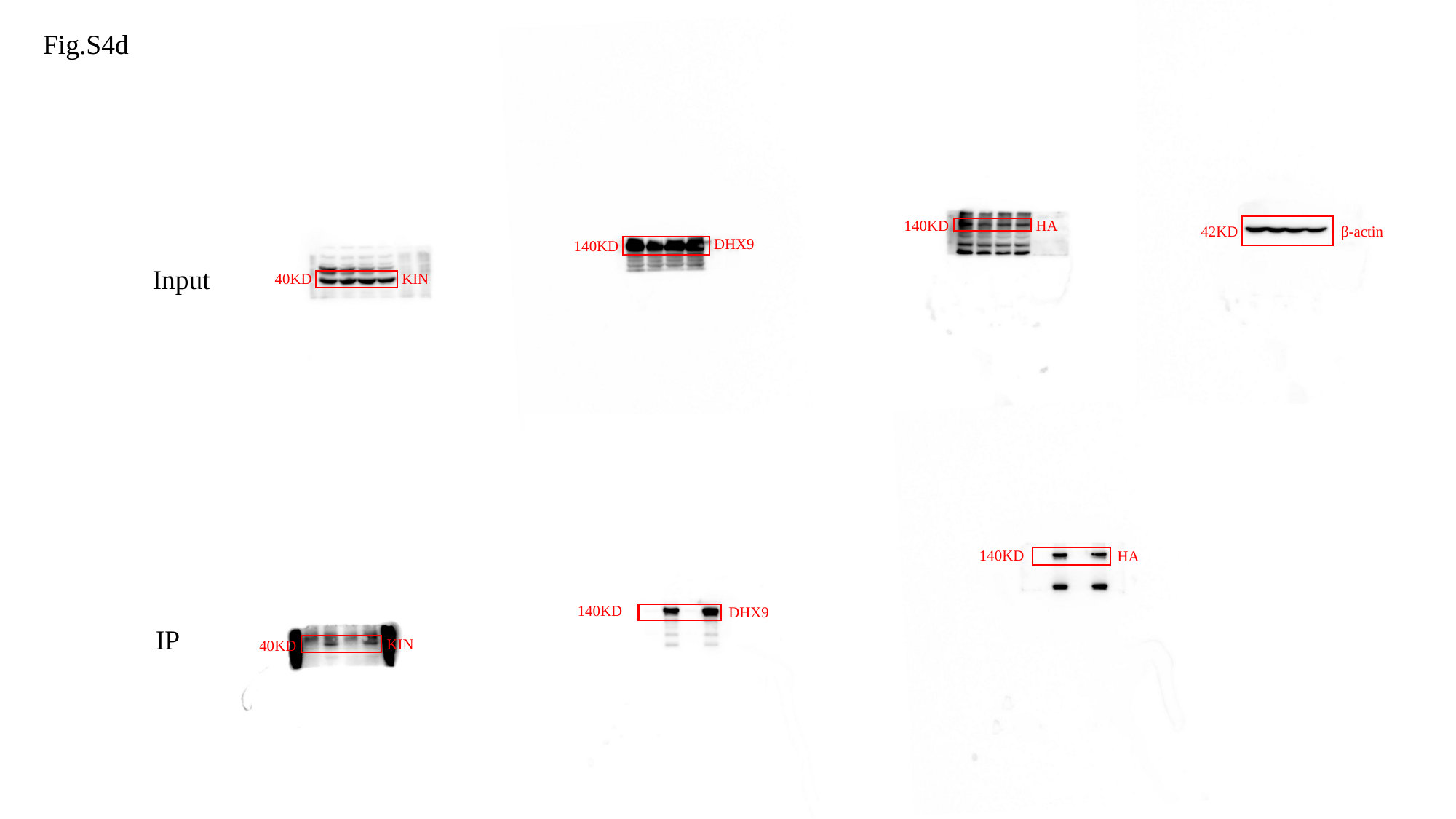

Fig.S4d
HA
140KD
42KD
β-actin
DHX9
140KD
Input
40KD
KIN
140KD
HA
140KD
DHX9
IP
KIN
40KD

## Slide 28
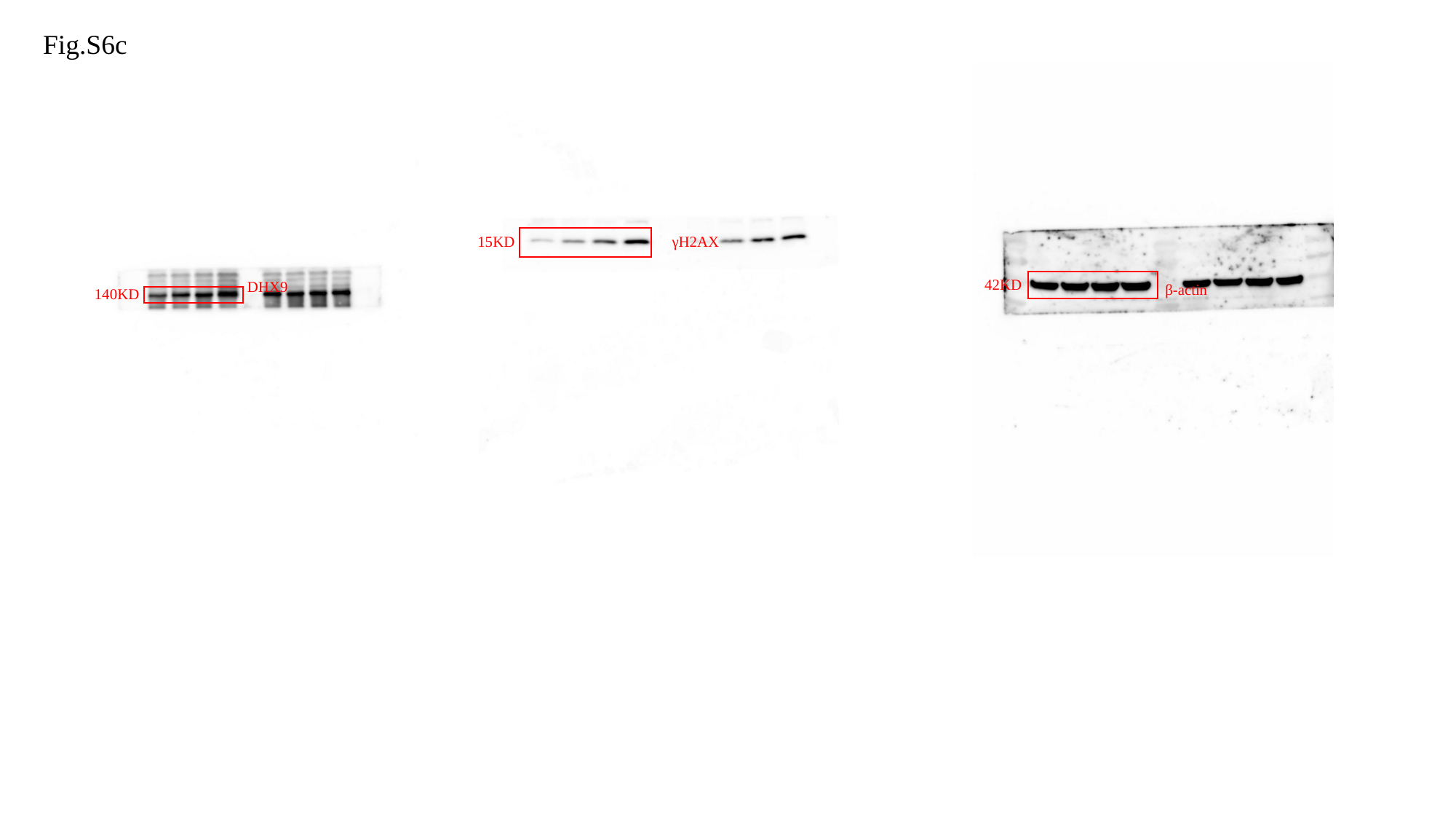

Fig.S6c
15KD
γH2AX
42KD
DHX9
β-actin
140KD

## Slide 29
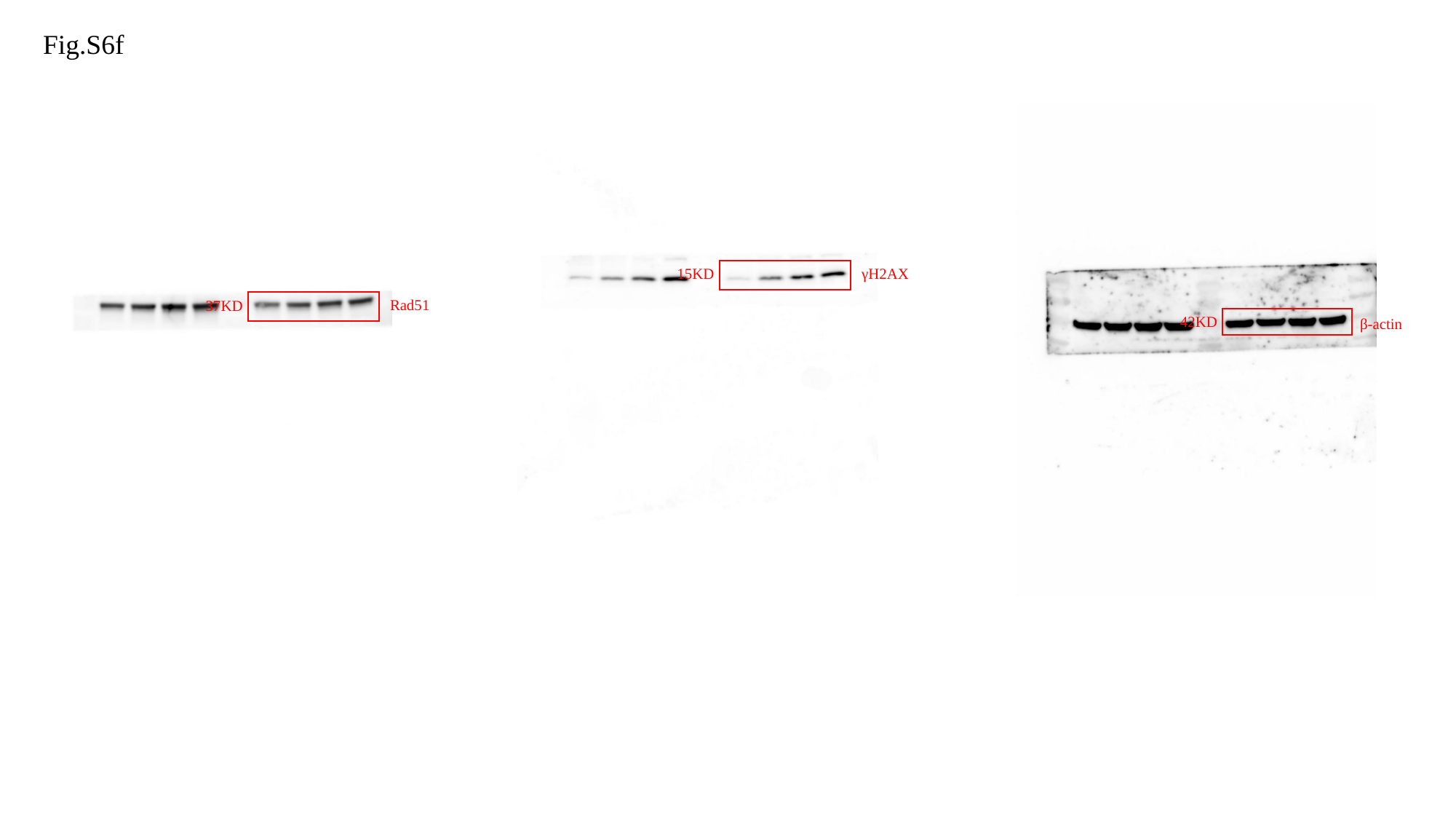

Fig.S6f
γH2AX
15KD
Rad51
37KD
42KD
β-actin

## Slide 30
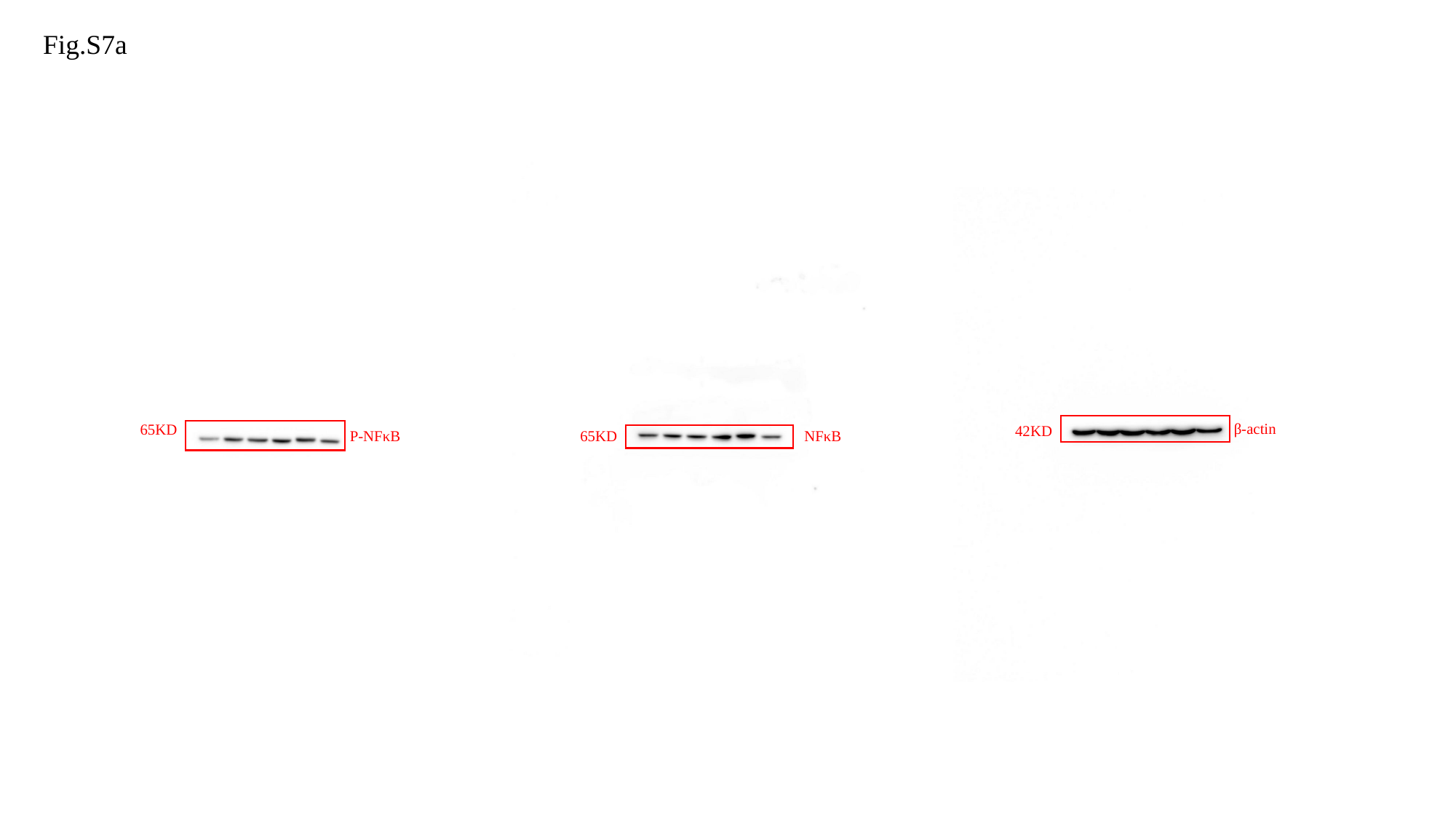

Fig.S7a
β-actin
65KD
42KD
NFκB
P-NFκB
65KD

## Slide 31
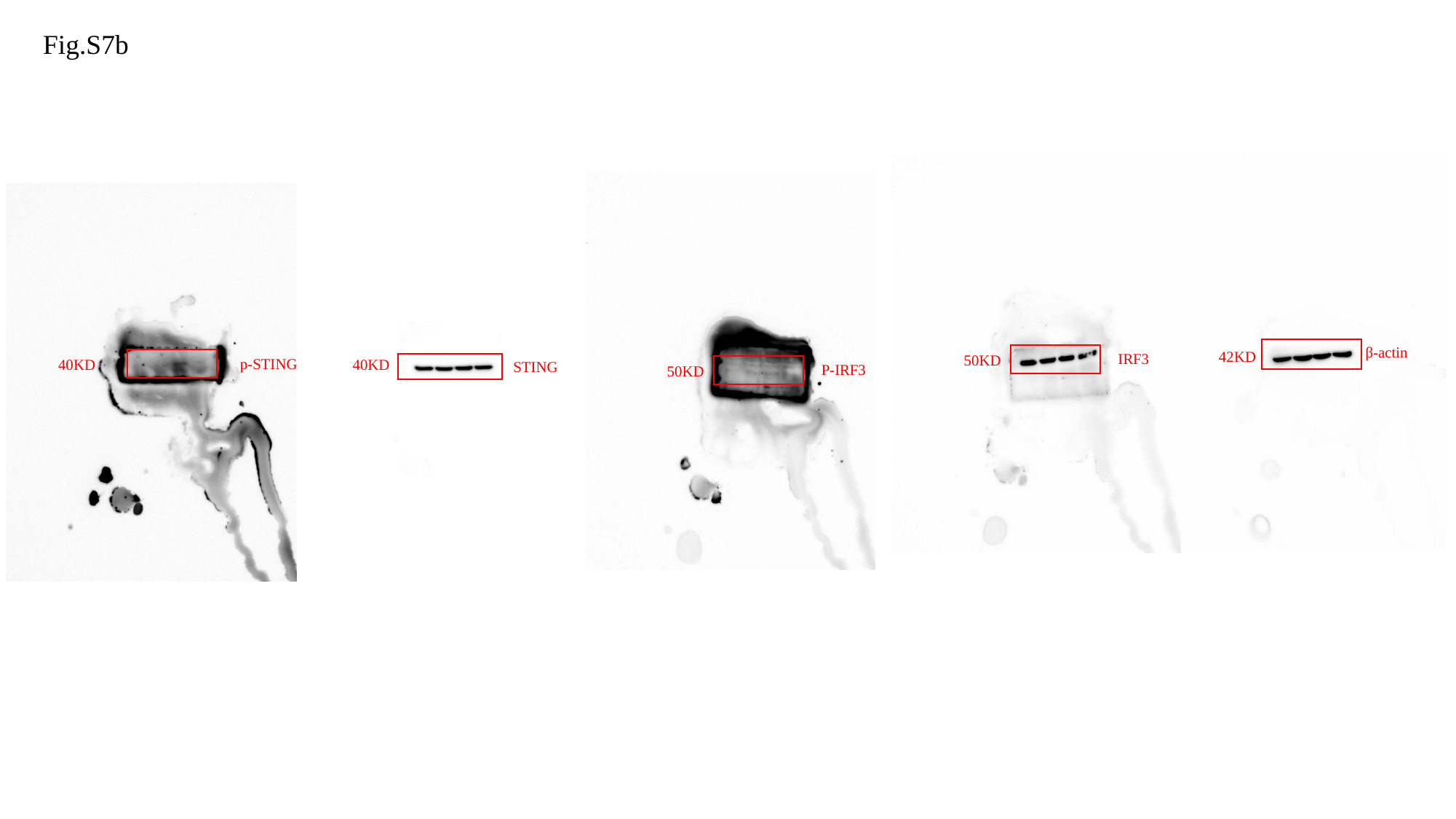

Fig.S7b
β-actin
42KD
IRF3
50KD
p-STING
40KD
40KD
STING
P-IRF3
50KD

## Slide 32
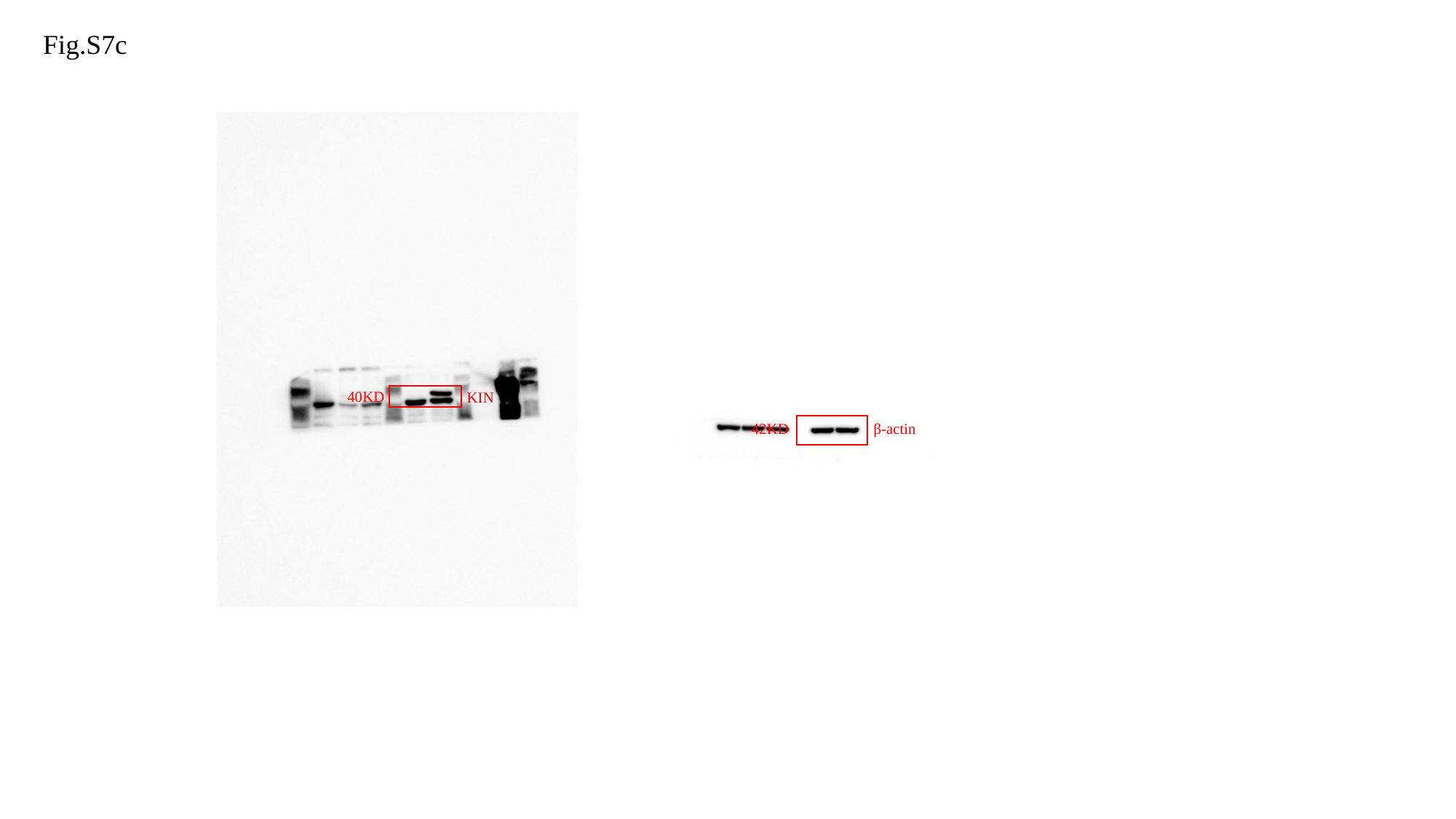

Fig.S7c
40KD
KIN
42KD
β-actin

## Slide 33
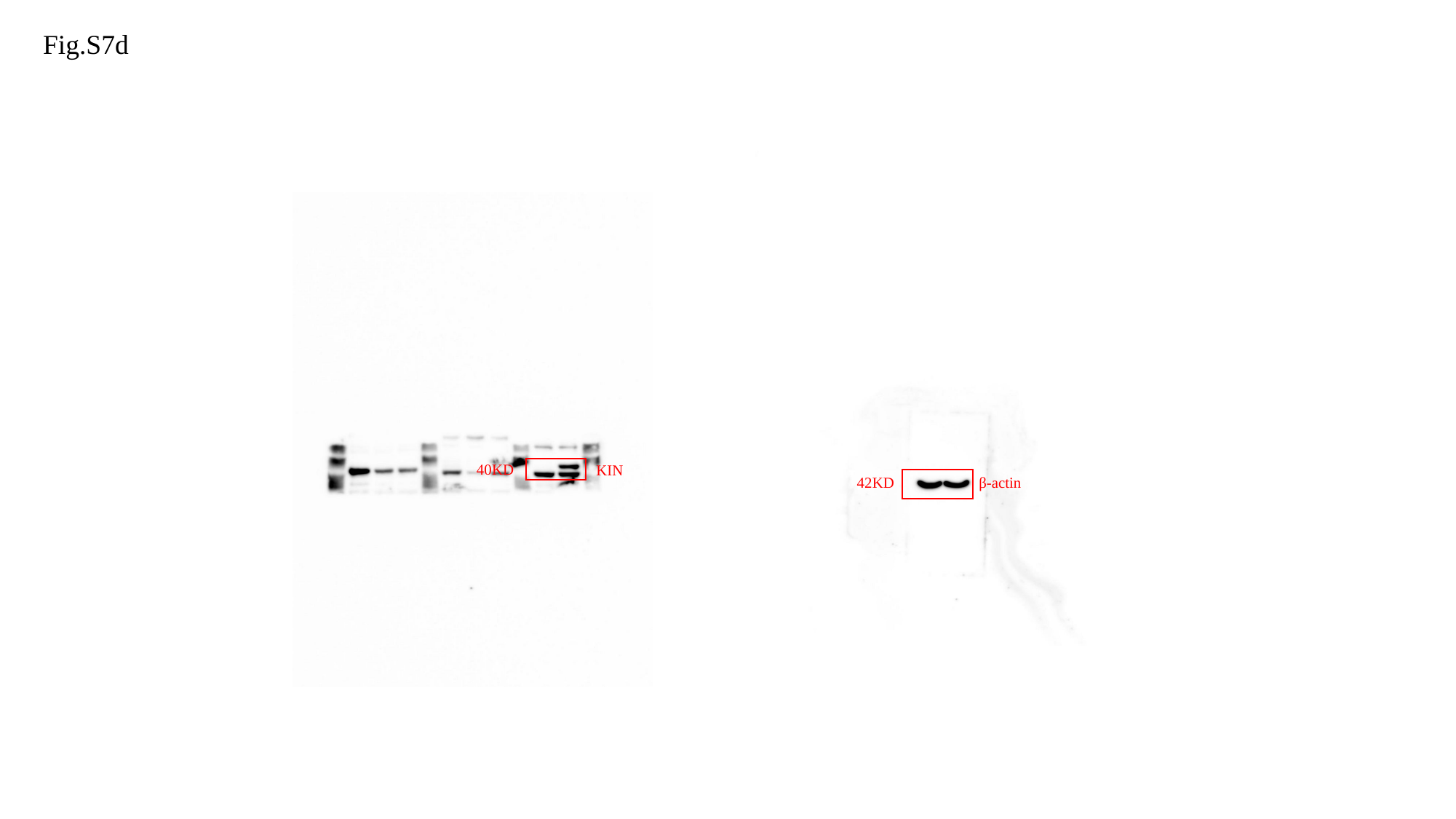

Fig.S7d
40KD
KIN
42KD
β-actin
