# Supplementary material for: DNA/RNA-binding protein KIN17 supports esophageal cancer progression via resolving noncanonical STING activation induced by R-loop
Source: Signal Transduct Target Ther. 2025 Aug 15;10:256. doi: 10.1038/s41392-025-02344-2 (PMC12354822; doi:10.1038/s41392-025-02344-2)
Supplement: Supplementary file 3 — The raw data of colony [file 41392_2025_2344_MOESM3_ESM.pptx]

## Slide 1
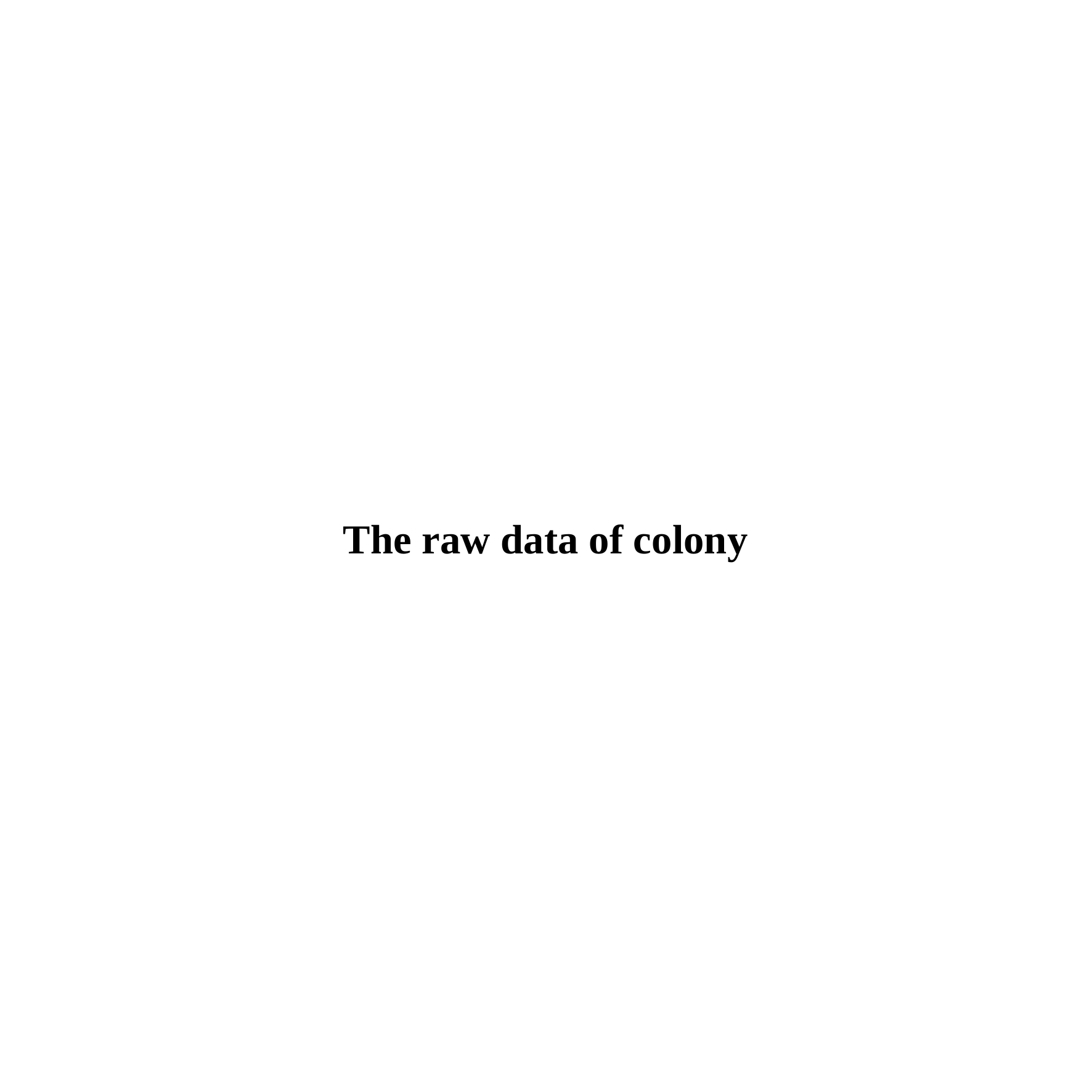

The raw data of colony

## Slide 2
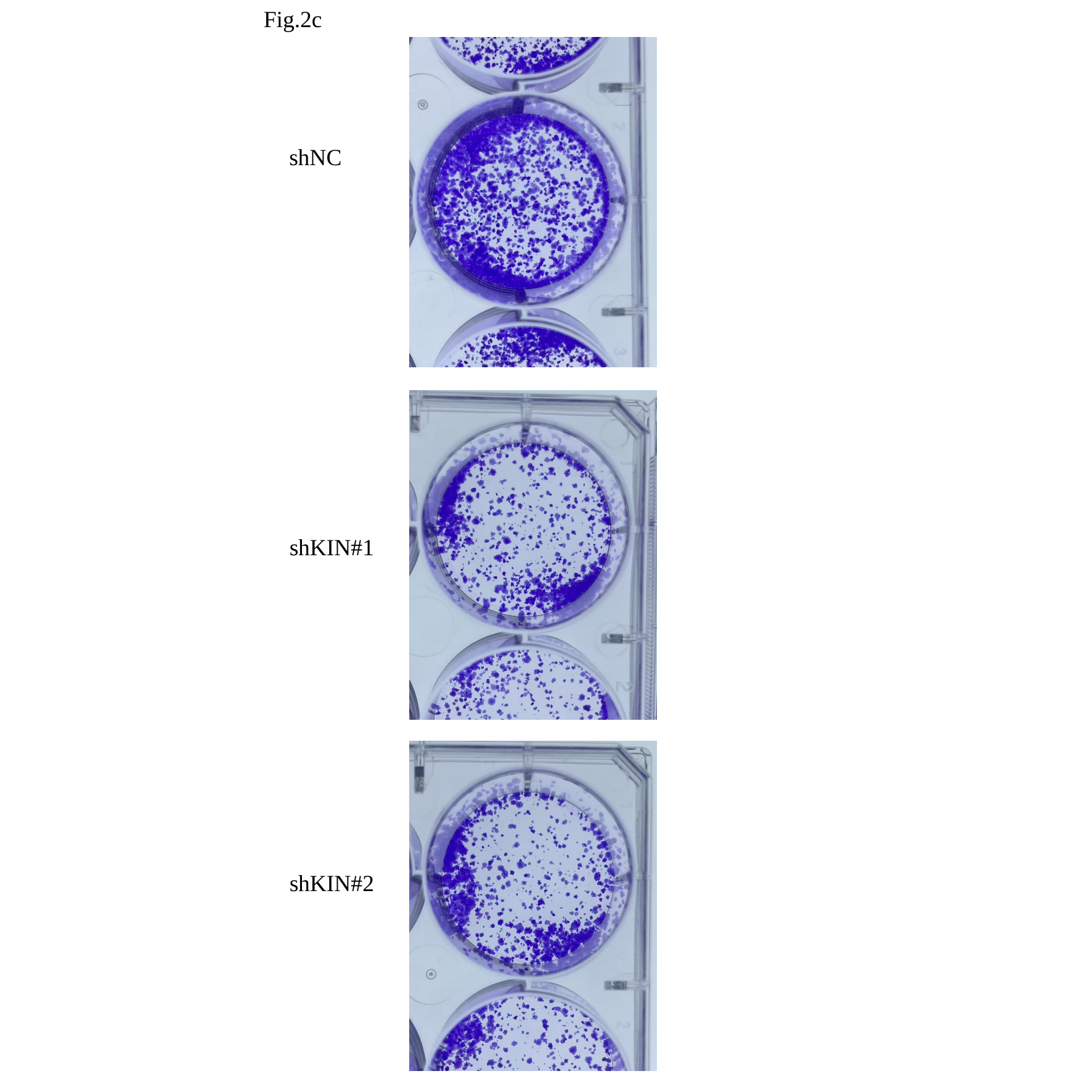

Fig.2c
shNC
shKIN#1
shKIN#2

## Slide 3
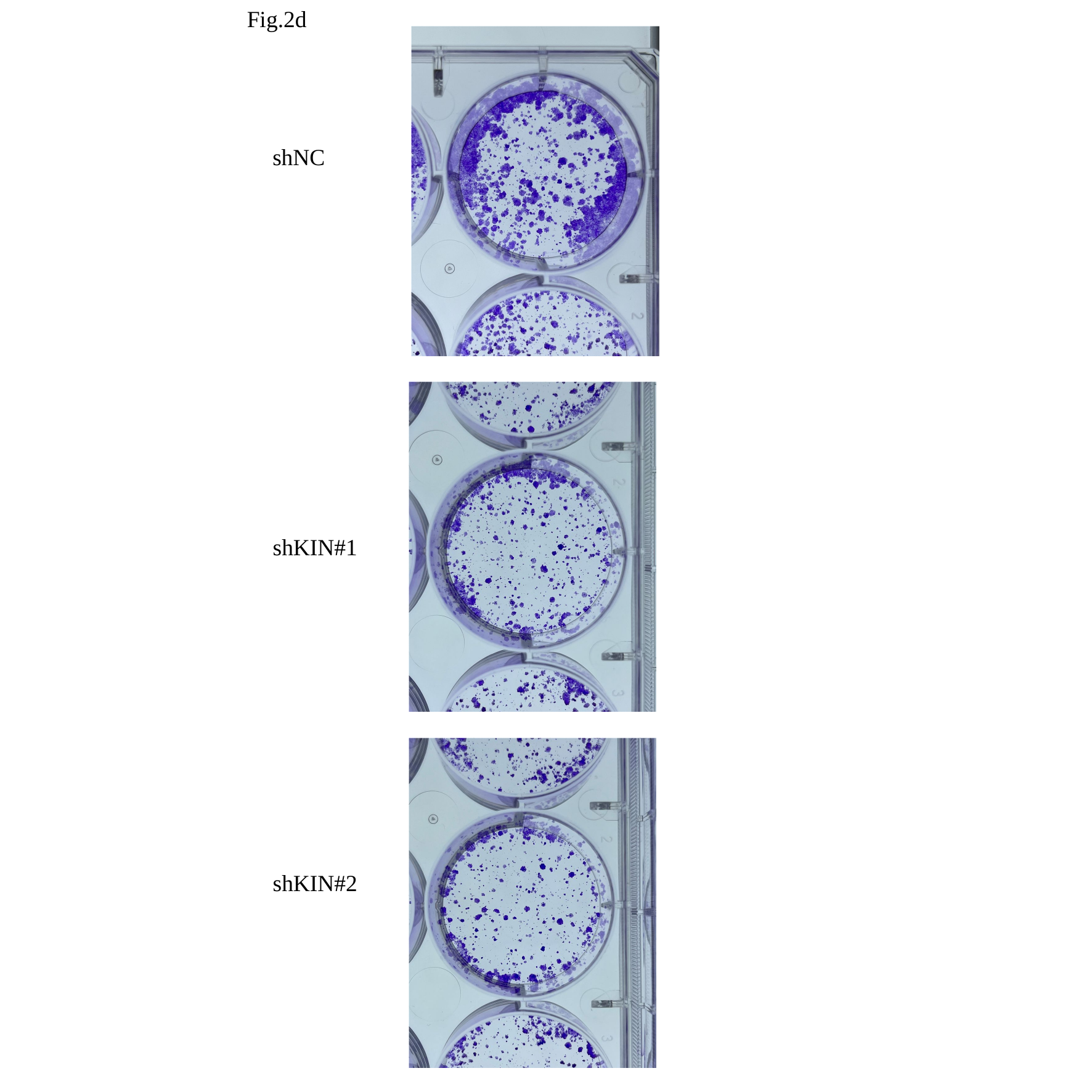

Fig.2d
shNC
shKIN#1
shKIN#2

## Slide 4
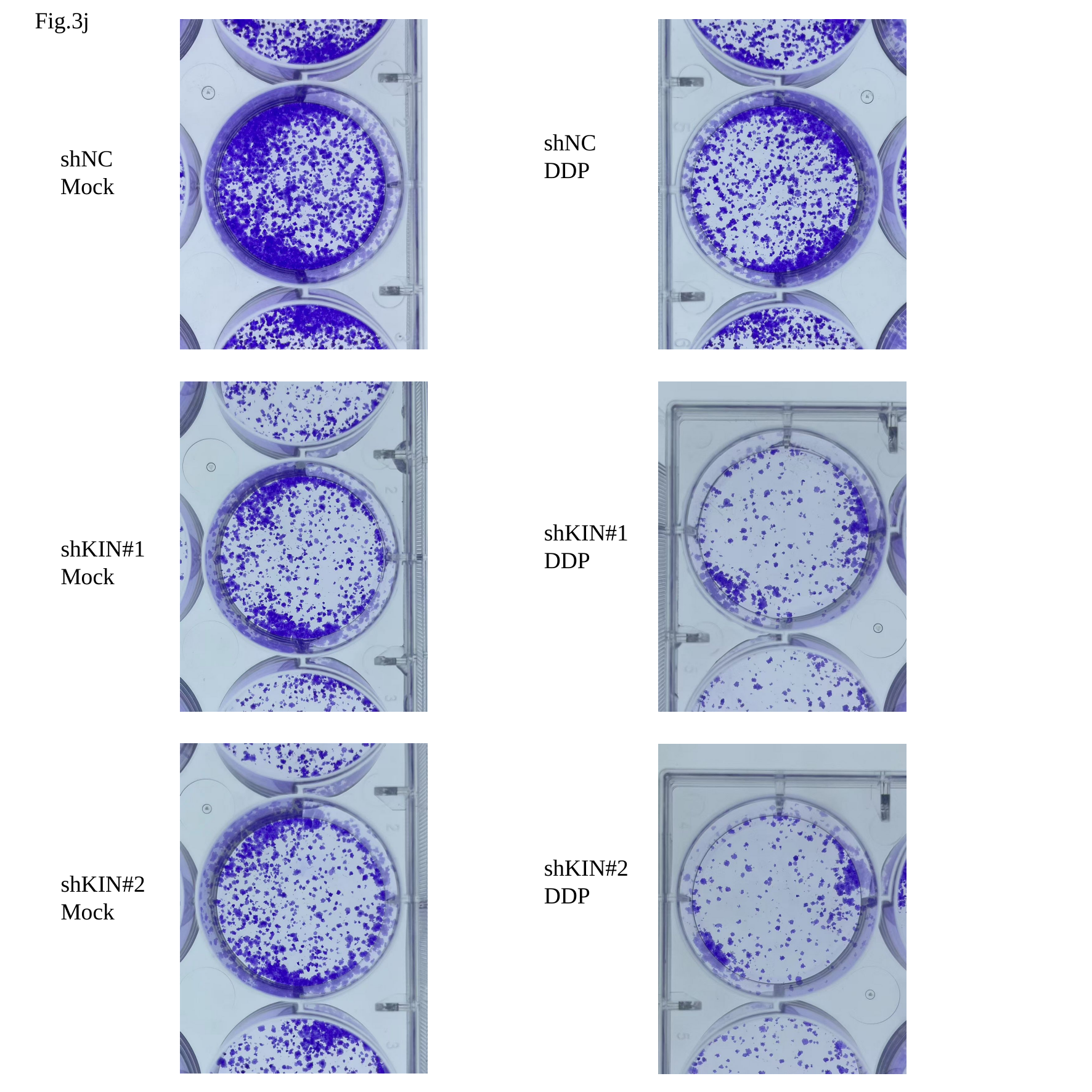

Fig.3j
shNC
DDP
shNC
Mock
shKIN#1
DDP
shKIN#1
Mock
shKIN#2
DDP
shKIN#2
Mock

## Slide 5
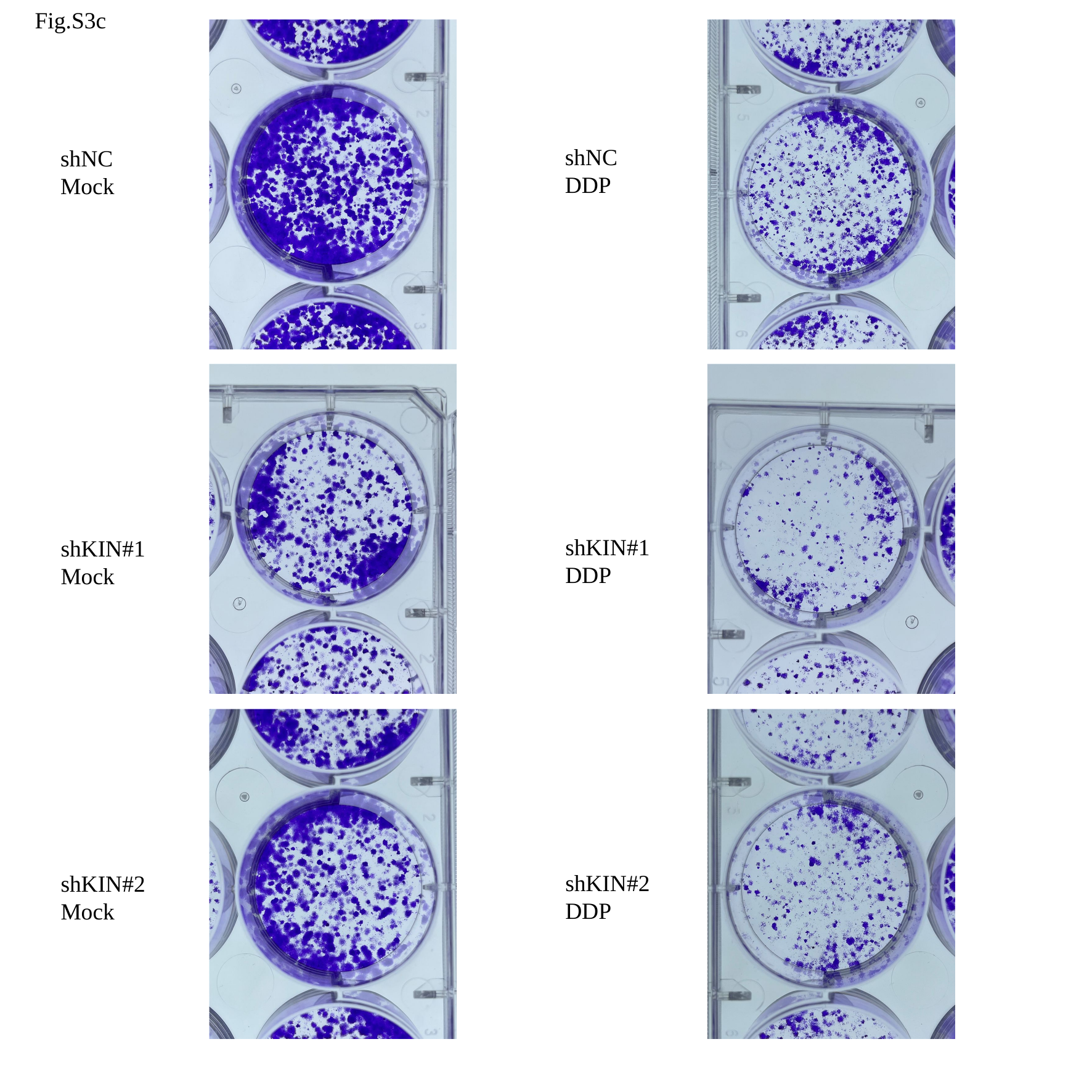

Fig.S3c
shNC
DDP
shNC
Mock
shKIN#1
DDP
shKIN#1
Mock
shKIN#2
DDP
shKIN#2
Mock
